# Climate change and conservation in a warm North American desert: effect in shrubby plants

**DOI:** 10.7717/peerj.6572

**Published:** 2019-03-07

**Authors:** Victoria Sosa, Israel Loera, Diego F. Angulo, Marilyn Vásquez-Cruz, Etelvina Gándara

**Affiliations:** 1 Biología Evolutiva, Instituto de Ecología AC, Xalapa, Veracruz, Mexico; 2 Facultad de Ciencias Biológicas, Benemérita Universidad Autónoma de Puebla, Mexico

**Keywords:** Arid-adapted plants, Chihuahuan desert, Conservation planning, Desert biota, Environmental resistance, Haplotype diversity, Protected natural areas

## Abstract

**Background:**

Deserts are biologically rich habitats with a vast array of animals and plants adapted to xeric conditions, and most deserts are among the planet’s last remaining areas of total wilderness. Among North American deserts, the Chihuahuan Desert has the highest levels of diversity and endemism. To understand the effect of future climate change on plants distributed in this arid land and propose effective conservation planning, we focused on five endemic shrubby species that characterize the Chihuahuan Desert and used an integrative approach.

**Methods:**

Ecological niche-based modeling, spatial genetics and ecological resistance analyses were carried out to identify the effect of global warming on the studied five shrubby species. Key areas that need to be preserved were identified taking into account the existing protected areas within the Chihuahuan Desert.

**Results:**

The extent of future distribution will vary among these species, and on average expansion will occur in the western part of the Chihuahuan Desert. For most species low environmental resistance to gene flow was predicted, while higher future resistance was predicted for one species that would lead to increased population isolation. The highest haplotype diversity was identified in three hotspots. Based on future suitability of habitat and in the haplotype diversity we suggest preserving two hotspots of genetic diversity in the Sierra Madre Oriental, located in areas without protection. The third hotspot was detected in the well preserved Tehuacán-Cuicatlán Man and Biosphere Reserve.

**Conclusion:**

Global climate change will have an effect in arid adapted plants, favoring expansion in the western of the Chihuahuan Desert however negatively affecting others with high ecological resistance disrupting gene flow. Two hotspots of genetic diversity in the Sierra Madre Oriental should be protected.

## Introduction

The effect of future climate change on organisms has been studied from multiple perspectives, in diverse biomes, ecosystems, farming landscapes and for many taxa and functional groups of organisms. The main effects of climate change are associated with demographic threats, opportunities for expansion, and positive or negative implications with respect to the size and position of species ranges, as well as the genetic variability of populations, disequilibrium in biotic interactions and a variable capacity of adaptation in natural and agricultural systems (Harris et al., 2006; Kelly & Goulden, 2008; Williams et al., 2010; Thornton et al., 2014; Anadón, Sala & Maestre, 2014; García et al., 2014; Corlett & Westcott, 2013; Skogen, Helland & Kaltenborn, 2018).

Understanding how future climate variation influences ecological and evolutionary processes in organisms is crucial to conservation decision-making. Furthermore, estimating genetic diversity among populations and their spatial distribution is valuable for determining their degree of vulnerability (Sgrò, Lowe & Hoffmann, 2011); areas with high genetic diversity should have priority for conservation owing to is importance for building resiliency in populations (Forester, DeChaine & Bunn, 2013). Often, genetic variability has already decreased where vulnerability to changes in climate is highest, and will deteriorate further precisely in the areas where diversity may be most needed for future persistence (Franks & Hoffmann, 2012). Complementary approaches to the assessment of genetic variation include methods that identify the ecological resistance of populations given that data on dispersal and gene flow are essential for conserving species in fragmented landscapes (McRae & Beier, 2007). It has been demonstrated that gene flow rates in plants vary enormously depending on the species and populations involved (Ellstrand, 2014); moreover, the effect of gene flow might still be significant at distance of thousands of meters acting as a connecting force for local selection of populations (Ellstrand, 2014). Thus, identifying genetic polymorphisms leading to population differentiation depending on genetic flow in populations and their connectivity in future climate scenarios will be practical for setting conservation planning (Ellstrand & Elam, 1993). An additional instrument for estimating the effect of future climate conditions on species is ecological niche-based modeling, which can provide useful ways to integrate future climate scenarios into conservation (Wiens, Stralberg & Jongsomjit, 2009; Forester, DeChaine & Bunn, 2013). Ecological niche and gene flow are causally interrelated with the potential for one to impact the other, thus studying both simultaneously should provide stronger predictions of future species distributions.

**Figure 1:**
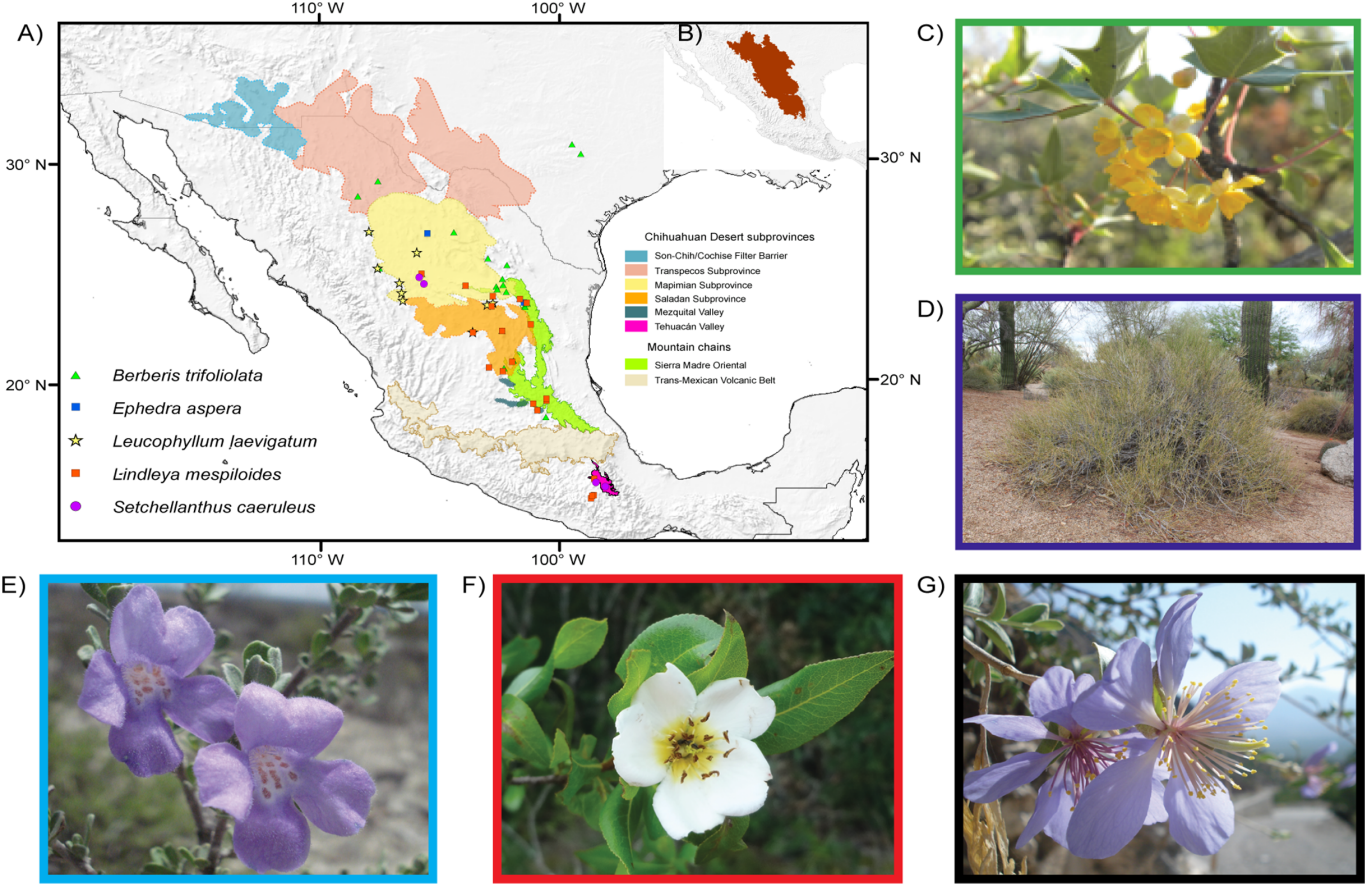
Distribution and images of the studied species. Limits of the Chihuahuan Desert and distribution map and images of the five plant species studied. Main biogeographic areas are indicated in addition to occurrence localities. Areas follow Shreve (1942) and Morafka (1977). (A) Chihuahuan Desert subprovinces and distribution of studied species. (B) Delimitation of the Chihuahuan Desert. (C) *Berberis trifoliolata* (Berberidaceae). (D) *Ephedra aspera* (Ephedraceae). (E) *Setchellanthus caeruleus* (Setchellanthaceae). (F) *Leucophyllum laevigatum* (Scrophulariaceae). (G) *Lindleya mespiloides* (Rosaceae). Images taken by Diego F. Angulo, Israel Loera, Victoria Sosa, Etelvina Gándara and Marilyn Vásquez-Cruz, respectively.

Just like any habitat, deserts will be affected by climate change. These biomes are biologically rich habitats with a vast array of animals and plants adapted to xeric conditions, and several deserts are among the Planet’s last remaining areas of total wilderness (Ward, 2009; Cooke, Warren & Goudie, 2013). Although the individual deserts of North America and northern Mexico are very small compared with the vast deserts of the Sahara, Arabia, Australia and Asia, the origin and evolution of their biota have led to a unique, high degree of elevated diversity (Riddle & Hafner, 2006; Wilson & Pitts, 2010). The most diverse of North American Deserts is the warm Chihuahuan Desert whose limits to the north are in the mountains of Arizona-New Mexico, and include western Texas, the Mexican Plateau to Hidalgo and Querétaro and a southern relict area, the Tehuacán Valley (Fig. 1, Henrickson & Johnston, 1986; Wilson & Pitts, 2010). The insularity of the Chihuahuan Desert has produced an area rich in endemic plant species; of the approximately 3,500 species about one third are endemic (Villarreal-Quintanilla et al., 2017).

There are few protected areas located within boundaries of the Chihuahuan Desert. The largest are the four biosphere reserves—Barranca de Metztitlán, la Michilía, Mapimí, and Tehuacán-Cuicatlán—and there are smaller protected areas such as Cuatrociénegas (National Commission of Natural Protected Areas, CONANP, www.gob.mx/conanp). The little research that there is on the effect of future climate change on the flora of the Chihuahuan Desert has focused on plants such as the cacti (Aragón-Gastélum et al., 2014; Shryock, Esque & Hughes, 2014; Carrillo-Angeles et al., 2016) or on ecosystems such as grasslands that are frequently dominated by *Larrea* and *Prosopis* (Huenneke et al., 2002; Báez et al., 2013; Bell et al., 2014).

Here, we focus on the shrubby plants of the Chihuahuan Desert and using prospective ecological niche-based modeling (ENM) in five endemic species, aim to estimate whether species ranges will vary or remain the same and to identify the areas that are more likely to remain stable. Additionally, by acquiring molecular data we estimate genetic diversity of populations of these species and determine how it is spatially distributed to determine the degree of vulnerability, taking into account the ecological connectivity of the populations. The goal is to provide guidelines for conservation decision-making based on these data and the existing protected areas.

Using these five indicator endemic shrubby plants we aim to: (1) determine how genetic diversity is spatially distributed in the Chihuahuan Desert; (2) establish the effect of global climate change on their distribution based on predictions of future climate scenarios for 2070; (3) understand how habitat ecological resistance among species might be affected by future climate change; (4) identify the most vulnerable or resilient populations and evaluate if they are located within protected areas; (5) make recommendations for conservation decision-making.

## Materials and Methods

**Field study permissions**. We obtained collecting permits to conduct this work from the Secretaría de Medio Ambiente y Recursos Naturales, Instituto Nacional de Ecología, Dirección General de Vida Silvestre (permit number: Registro de Colección Científica VER-FLO-228-09-09).

**Study species**. We selected five endemic species to the Chihuahuan Desert (Fig. 1). The first is the Mormon tea gymnosperm *Ephedra aspera* Engelm. ex S. Watson, a wind-pollinated species with rugged yellowish stems, dry seed cones and large seeds dispersed by small mammals (Hollander & Wall, 2009). The four other species are angiosperms. *Berberis trifolilata* Moric. (transferred to *Alloberberis* by Chih-Chieh & Kuo-Fang, 2017), known as algerita or agarito, is a small to large shrub with gray or blue foliage and leaflets with sharp spines, its yellow flowers are clustered and fragrant (Angulo, Sosa & García-Franco, 2014). *Leucophyllum laevigatum* Standl. known as Chihuahuan Sage or blue Texas ranger, is one of the most showy shrubs in the desert and produces exuberant bright violet to purple small flowers that attract butterflies and bees; its plants are cultivated in nurseries and used as ornamentals in Texas and Arizona (Henrickson & Flyr, 1985). The rose species *Lindleya mespiloides* Kunth is the only species of a monotypic genus endemic to the Chihuahuan Desert, with a shrubby or tree-like life form with thick leaves, conspicuous white flowers and dry, capsular fruits (Henrickson, 2012). *Setchellanthus caeruleus* Brandegee is the sole species of Setchellanthaceae, a family endemic to the Chihuahuan Desert, and is receiving much interest because it produces glucosinolates and belongs to the group of mustard plants. Its shrubs or small trees have corky branches and showy lavender-violet flowers; populations are few and far between and have been collected only in the north of the desert and in the Tehuacán Valley (Iltis, 1999).

**Sampling and DNA sequencing**. We collected samples from populations of *E. aspera* and *Leucophyllum laevigatum* in their entire range. Protocols for extraction, amplification and sequencing including the DNA markers with their corresponding primers are the same utilized in previous research (Gándara & Sosa, 2014; Loera, Ickert-Bond & Sosa, 2017). For the three remaining species we used previously published sequences (*B. trifoliolata*: Angulo et al., 2017, *Lindleya mespiloides*: Vásquez-Cruz & Sosa, 2016; *S. caeruleus*: Hernández-Hernández, Colorado & Sosa, 2013) (Fig. 1; see Table 1 for localities). The plastid markers sequenced were *trnF trnL, rpl32-trnL, trnH-psbA* and *psbI-psbK*. GenBank accessions representing all haplotypes in the populations of each species are listed in Table S1.

**Table 1:** Studied populations with their georeferences and genetic diversity and haplotypes.

		*Berberis trifoliolata*			*Ephedra aspera*					*Leucophyllum laevigatum*				*Lindleya mespiloides*				*Setchellanthus caeruleus*			
Sample location	Code	Lat	Long	*h*	Lat	Long	*h*	π	π	Lat	Long	*h*	π	Lat	Long	*h*	π	Lat	Long	*h*	π
México, Acatitlán, Qro	ACT	–	–	–	21.2	−99.21	0.25	0.001	–	–	–	–	–	–	–	–	–	–	–	–	–
Mexico, Arteaga, Coah	ART	25.40	−100.80	0.5	–	–	–	–	0.0004	–	–	0	0	–	–	–	–	–	–	–	–
Mexico, borderline Coah-Zac	FCZ	24.98	−101.18	0	24.98	−101.18	0.56	0.0034	0	–	–	0.47	0.00024	24.68	−101.40	1	0.00457	–	–	–	–
Mexico, Cerro El Potosí, NL	CEP	24.89	−100.19	0.47	−100.18	24.88	0.77	0.0054	0.0008	–	–	–	–	–	–	–	–	–	–	–	–
Mexico, Chih	CHIH	28.60	−106.12	0.39	–	–	–	–	0.0003	–	–	–	–	–	–	–	–	–	–	–	–
Mexico, Cuatro Cienegas, Coah	CC	27.30	−102.61	0.69	–	–	–	–	0.001	–	–	0.66	0.00034	–	–	–	–	–	–	–	–
México, Galeana, NL	GAL	–	–	–	–	–	–	–	–	24.75	−100.04	0.4	0.0002	–	–	–	–	–	–	–	–
Mexico, Guadalcazar, SLP	GUAD	22.65	−100.43	0.17	22.61	−100.47	0.8	0.0038	0.0001	–	–	–	–	–	–	–	–	–	–	–	–
Mexico, Ixmiquilpan, Hgo	IXM	20.61	−99.24	0.21	–	–	–	–	0.0003	–	–	–	–	–	–	–	–	–	–	–	–
México, Jicotlán, Oax	JIC	–	–	–	17.78	−97.48	0.53	0.0022	–	–	–	–	–	–	–	–	–	–	–	–	–
México, La Angostura, Coah	ANG	25.34	−101.05	0	–	–	–	–	0	–	–	–	–	–	–	–	–	–	–	–	–
Mexico, La Gavia, Coah	LG	26.35	−101.36	0.89	–	–	–	–	0.001	–	–	–	–	–	–	–	–	–	–	–	–
México, La Lagunita, Qro	LAG	–	–	–	21.27	−99.21	0.77	0.0039	–	–	–	–	–	–	–	–	–	–	–	–	–
Mexico, La Zarca, Dgo	ZAR	–	–	–	–	–	–	–	–	–	–	–	–	25.46	−104.60	0	0	–	–	–	–
México, Laguna Seca, SLP	LSE	–	–	–	22.27	−100.81	0.25	0.0005	–	–	–	–	–	–	–	–	–	–	–	–	–
Mexico, Lerdo, Dgo	LER	–	–	–	–	–	–	–	–	–	–	–	–	–	–	–	–	25.43	−103.7	0	0
México, Maconí, Qro	MAC	–	–	–	20.85	−99.55	0.9	0.0037	–	–	–	–	–	–	–	–	–	–	–	–	–
Mexico, Mapimí, Dgo	MAP	–	–	–	–	–	–	–	–	–	–	–	–	26.57	−103.97	0.66	0.00115	25.67	−103.87	0.25	0.00027
Mexico, Matehuala SLP	MAT	25.14	−100.69	0.68	–	–	–	–	0.002	–	–	–	–	–	–	–	–	–	–	–	–
Mexico, Ojinaga, Chih	OJIN	29.15	−105.39	0.4	–	–	–	–	0.0007	–	–	–	–	–	–	–	–	–	–	–	–
Mexico, Ojuelas, Dgo	OJU	25.80	−103.78	0.4	25.79	−103.79	1	0.0083	0.0003	–	–	–	–	–	–	–	–	–	–	–	–
Mexico, Pablillo, NL	PAB	24.61	−100.00	0	–	–	–	–	0	–	–	–	–	–	–	–	–	–	–	–	–
Mexico, Parral, Chih	PARRAL	27.32	−105.72	0.6	–	–	–	–	0.0007	–	–	0	0	27.32	−105.72	0.4	0.00138	–	–	–	–
Mexico, Parras, Coah	PARR	25.36	−102.18	0.91	25.36	−102.17	0.6	0.0037	0.002	–	–	0	0	–	–	–	–	–	–	–	–
México, Peña Miller, Qro	MIL	–	–	–	21.09	−99.69	0.58	0.0023	–	–	–	–	–	–	–	–	–	–	–	–	–
México, Ramos Arizpe, Coah	RAZ	25.61	−100.83	0.5	–	–	–	–	0.0004	–	–	–	–	–	–	–	–	–	–	–	–
Mexico, Rancho Jaguey, Coah	RJ	25.23	−101.02	0	–	–	–	–	0	–	–	–	–	–	–	–	–	–	–	–	–
Mexico, Real de Catorce SLP	RC	23.74	−100.85	0.81	23.73	−100.84	0.73	0.0018	0.002	–	–	0	0	–	–	–	–	–	–	–	–
Mexico, Rocamontes, Dgo	ROCA	24.74	−101.18	0	24.62	−101.23	0.6	0.0037	0	–	–	0	0	24.74	−101.18	0.8	0.0016	–	–	–	–
Mexico, Rodeo, Dgo	ROD	–	–	–	–	–	–	–	–	–	–	–	–	25.11	−104.53	0.07	0.00057	–	–	–	–
Mexico, San Juan del Rio, Dgo	ATO	–	–	–	–	–	–	–	–	–	–	–	–	24.84	−104.48	0.82	0.0000001	–	–	–	–
Mexico, San Pedro Iturbide, NL	SPI	24.72	−99.91	0.33	24.74	−99.93	0.47	0.0019	0.0002	–	–	–	–	–	–	–	–	–	–	–	–
Mexico, Santa María del Oro, Dgo	StaMO	25.99	−105.33	0	–	–	–	–	0	–	–	–	–	26.00	−105.40	0.7	0.00092	–	–	–	–
México, Santa Teresa, SLP	TER	–	–	–	22.4	−101.32	0.5	0.001	–	–	–	–	–	–	–	–	–	–	–	–	–
Mexico, Sierra de San Miguel, NL	SSM	26.11	−100.66	0.4	–	–	–	–	0.001	–	–	–	–	–	–	–	–	–	–	–	–
México, Sierra Mojada, Coah	MOJ	–	–	–	–	–	–	–	–	27.26	−103.58	0	0	–	–	–	–	–	–	–	–
México, Tamazulapam, Oax	TAM	–	–	–	17.66	−97.58	0.34	0.0007	–	–	–	–	–	–	–	–	–	–	–	–	–
Mexico, Teotitlán, Oax	TEO	–	–	–	–	–	–	–	–	–	–	–	–	–	–	–	–	18.09	−97.06	1	0.00815
México, Teotongo, Oax	TT	–	–	–	17.76	−97.54	0.75	0.0026	–	–	–	–	–	–	–	–	–	–	–	–	–
Mexico, Trópico de Cáncer, Zac	TCA	–	–	–	23.67	−101.91	0.71	0.0049	–	–	–	–	–	23.68	−101.92	0	0	–	–	–	–
Mexico, Ventura, SLP	VEN	22.38	−100.77	0.17	–	–	–	–	0.0001	–	–	–	–	–	–	–	–	–	–	–	–
Mexico, Zapotitlán de Salinas Pue	TEH	–	–	–	18.37	−97.47	0.83	0.0044	–	–	–	–	–	–	–	–	–	18.31	−97.47	0.75	0.00163
Mexico, Zaragoza, NL	ZAG	–	–	–	23.97	−99.79	0.93	0.00045	–	–	–	–	–	–	–	–	–	–	–	–	–
US Austin, Texas	AUST	30.14	−97.96	0	–	–	–	–	0	–	–	–	–	–	–	–	–	–	–	–	–
US Purola, Texas	PUR	30.49	−98.2	0.85	–	–	–	–	0.001	–	–	–	–	–	–	–	–	–	–	–	–

**Note:**

Populations studied, indicating population abbreviation, number of plants, geographic region, latitude and longitude where they were collected, as well as molecular diversity indices and their respective haplotypes. San Luis Potosi (SLP), Coah (Coahuila), Zacatecas (Zac), Nuevo Leon (NL), Durango (Dgo), Chihuahua (Chih), Hidalgo (Hgo), Haplotype diversity (*h*), nucleotide diversity (π).

**Genetic analyses**. Parameters of population diversity, such as haplotype diversity (*h*) and nucleotide diversity (π), were estimated for each population using DnaSP (Librado & Rozas, 2009). Haplotype genetic diversity (*h*) was interpolated for each species using a distance weighted interpolation in ArcView v. 3.2 (ESRI, Redlands, CA, USA). In order to visualize the genetic diversity on a map, we plotted this interpolation over a hill shade map obtained from the digital elevation model Hydro1K. We then averaged the genetic diversity of all species to identify the areas that share the highest genetic diversity. Genetic differentiation among populations of the studied species was estimated by pairwise *Fst* with an analysis of molecular variance with ARLEQUIN ver. 3.5.1.2 (Excoffier & Lischer, 2010) with 1,000 simulations performed to test significance of covariance components and fixation indices.

**Ecological niche-based modeling**. The geographical coordinates of the species utilized in this study were collected in the field and complemented with information consulted in the following herbaria: ARIZ, ENCB, MEXU, TEX and XAL as well as from the Global Biodiversity Information Facility GIBIF (www.gbif.org) to carry out Ecological Niche Modeling (ENM). They comprised the entire distribution of species (the number of records used in each species is given in Table S2). *S. caeruleus* is a relict taxon recorded in only two localities with a few individuals, and we considered all occurrences. We used the biogeographic sub-regions of the Chihuahuan Desert proposed by Morafka (1977, 1989) based on climate and distribution data for flora and fauna: Sonora-Cochise, Transpecos, Mapimian, Saladan and Rio Pánuco; we also included the Tehuacán Valley proposed by Shreve (1942) as a satellite area of the Chihuahuan Desert (Fig. 1). Considering a pixel of one km^2^, ENM was estimated for each species. Environmental inputs were based on the 19 climate variables from the WorldClim data base version 1.4 (Hijmans et al., 2005) at a 2.5 min resolution. These variables represent global precipitation and temperature conditions for the years 1960–1990.

Prior to estimating ENM’s we performed a paired Pearson correlation analysis based on the extracted environmental data of all the occurrence points. Then we selected a set of uncorrelated variables (Pearson correlation coefficients below 0.7) as environmental inputs. The latter resulted in a set of eight bioclimatic variables (i.e., BIO2 = Mean Diurnal Range (Mean of monthly (max temp—min temp), BIO4 = Temperature Seasonality (standard deviation *100), BIO5 = Max Temperature of Warmest Month, BIO6 = Min Temperature of Coldest Month, BIO9 = Mean Temperature of Driest Quarter, BIO12 = Annual Precipitation, BIO14 = Precipitation of Driest Month, BIO15 = Precipitation Seasonality (Coefficient of Variation). In addition, a principal component analysis (PCA) analysis was conducted to corroborate the most significant climate variables (see results in Fig. S1) using the statistical software R with the package *raster* (Thuiller et al., 2016). Species distribution modeling (SDM) were estimated using the maximum entropy algorithm (MaxEnt) implemented in the R package *biomod2* (Thuiller et al., 2016). For each species, occurrence data was divided using 70% of the records for training the models and 30% for testing them. A total of 10 replicates per each species were used and from these, geographic predictions and performance was averaged per species. Model performance was estimated for the projections in the climatic scenario for the present using the statistical metrics relative operating characteristic (ROC), TSS (true skill statistic) and Kappa. Values for ROC range from zero to one, with values greater than 0.5 meaning the model predicts testing points better than a random expectation. TSS and Kappa values range from −1 to 1 where values close to 0 indicate a prediction not different than random whereas positive values indicate predictions better than random. To integrate most of the variation observed in the bioclimatic variables, we alternatively performed a multivariate analysis. A PCA was performed using 19 bioclimatic variables of all the climatic scenarios used in this study and reduce the observed variation on a set of uncorrelated PC axes. Then, we used the PC scores of these axes to generate raster layers for each climate scenario as environmental inputs to estimate SDMs and perform geographic projections. The first six PC axes explained more than 95% of the observed variation and were used to estimate SDMs. Loading contributions for each of the PC axes used are showed in Table S4. PCA analyses were conducted in the statistical software R using the package *raster* (Thuiller et al., 2016).

To predict whether suitability conditions of each species might potentially change in the future we projected the estimated ENMs to future climate scenarios (i.e., for the year 2070). We used the “Representative Concentration Pathways” (RCPs) RCP 2.6 and 8.5 climate scenarios that represent the most optimistic and pessimistic scenarios, respectively. The RCPs are coherent with an ample range of probable changes in future human greenhouse gas emissions with the objective or representing their atmospheric concentrations. RCP 2.6 expects that global annual greenhouse gas emissions will reach the highest point between 2010 and 2020, and that they will descend substantially thereafter while the RCP 8.5 expects that emissions continue to rise during the 21st century (Meinshausen et al., 2011). In addition, two different general circulation models (GCMs) for each of these scenarios were used to obtain the geographic predictions. These GCMs were CCSM4 and MIROC-ESM. All future environmental layers were based on the WorldClim database version 1.4 (Hijmans et al., 2005). We averaged the suitability values of the two different climate simulations for each RCP scenario to perform geographic projections under future scenarios.

**Evaluation of protected areas and genetic diversity**. To identify whether the areas with the highest genetic diversity lie within protected areas we also overlaid protected areas on the distribution maps of the sampled populations. Protected area shape files from Mexico were downloaded from the National Commission of Protected Natural Areas (CONANP, www.gob.mx/conanp) and the world database on protected areas (https://www.iucn.org/theme/protected-areas). Protected areas in Texas were obtained from the Texas Parks and Wildlife Department (tpwd.texas.gov). Table 2 includes the protected areas within the Chihuahuan Desert.

**Table 2:** Preserved natural areas in the Chihuahuan Desert with extension and type.

Code	Name	Designation	Designation type	Area
0	Pico de Orizaba	National Park	National	0
1	Cofre de Perote	National Park	National	0
2	Gogorrón	National Park	National	0
3	Cumbres de Monterrey	National Park	National	1,773.96
4	Cuatrociénegas	Flora and Fauna Protection Area	National	843.47
5	Maderas del Carmen	Flora and Fauna Protection Area	National	2,083.81
6	Los Mármoles	National Park	National	231.5
7	Sierra Gorda	Biosphere Reserve	National	3,835.67
8	Cerro El Potosí	Area Subject to Ecological Conservation	National	9.8938
9	Cuenca Alimentadora del Distrito Nacional de Riego Don Martín	Natural Resources Protection Area	National	0
10	Huiricuta y la Ruta Histórica Cultural del Pueblo Huichol	Not reported	National	1,400
11	Ocampo	Flora and Fauna Protection Area	National	3,442.38
12	Real de Guadalcázar	Not reported	National	2,570
13	Serranía de Zapalinamé	Area Subject to Ecological Conservation	National	257.6868
14	Sierra y Cañón de Jimulco	Natural protected area and ecological reserve	National	604.5826
15	Zona de Restauración Ecológica del Lobo Mexicano San Joaquín de Soto	Certified	National	0
16	Tehuacán-Cuicatlán	Biosphere Reserve	National	4,901.87
17	Barranca de Metztitlán	Biosphere Reserve	National	960.43
18	Mapimí	Biosphere Reserve	National	3,423.88
19	Sierra de Alvarez	Flora and Fauna Protection Area	National	169
20	Río Sabinas	Ramsar Site, Wetland of International Importance	International	6,031.23
21	Laguna de Santiaguillo	Ramsar Site, Wetland of International Importance	International	240.16
22	Big Bend National Park	UNESCO-MAB Biosphere Reserve	International	2,832.47
23	Black Gap	State Wildlife Management Area	National	0
24	Mason Mountain	State Wildlife Management Area	National	0
25	Old Tunnel	State Wildlife Management Area	National	0
26	Honey Creek	State Natural Area	National	0

**Current and future environmental resistance to gene flow**. We estimated ecological resistance among populations using the habitat suitability raster file derived from ecological niche modeling as a conductance matrix in Circuitscape 4.0.5 (McRae & Beier, 2007). Circuitscape considers the landscape to be an electrical circuit in which populations serve as sources or sinks of electrical current, while environmental features either inhibit or assist the flow of that current by providing high or low resistance to the circuit(s) connecting the populations (McRae & Beier, 2007). Environmental resistance was averaged for each species considering current and future climate scenarios and plotted using boxplots.

## Results

### Genetic diversity and its spatial distribution

**Figure 2:**
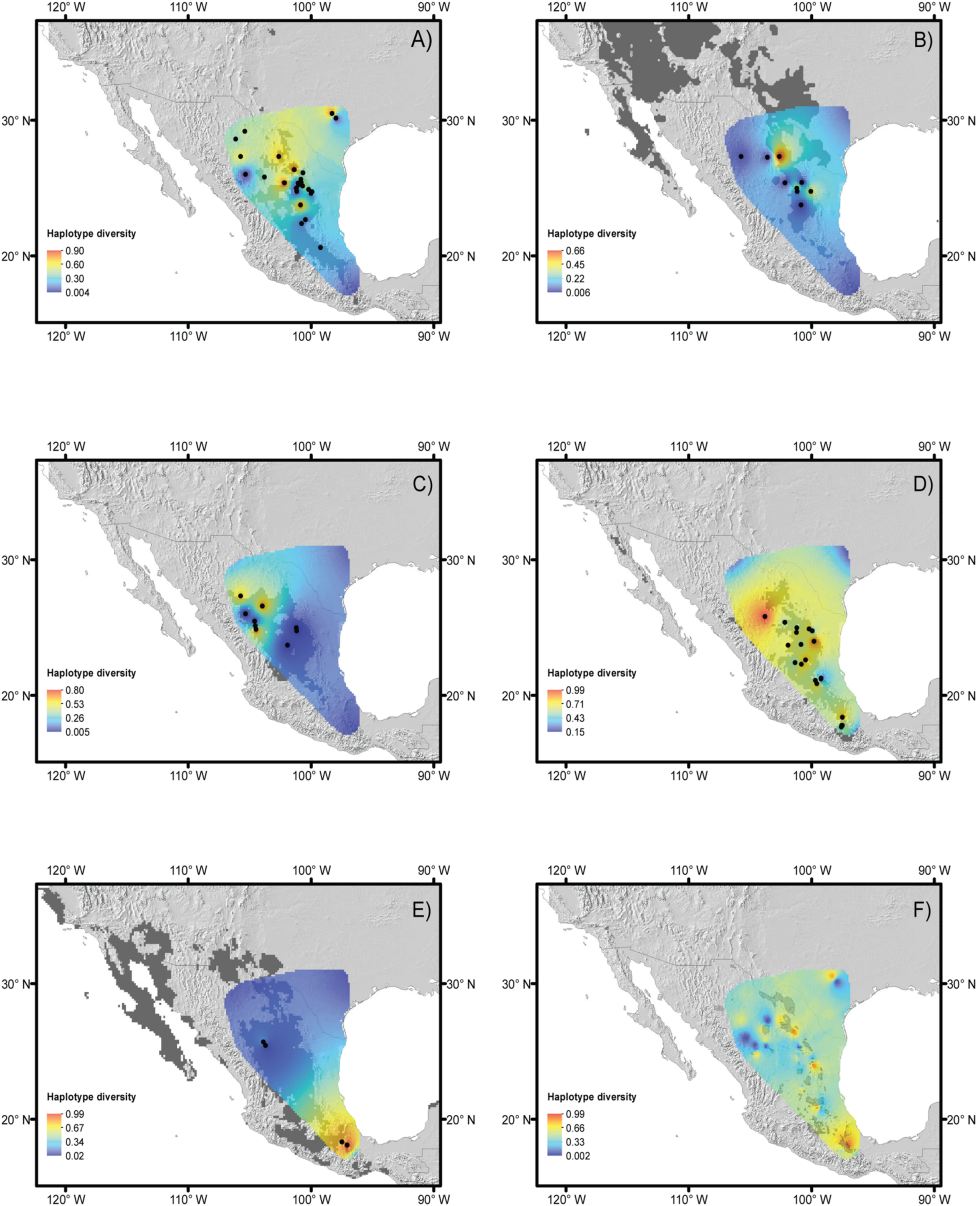
Haplotype diversity of the studied species. Landscape distributions of chloroplast haplotype polymorphism in the five species studied. (A) *Berberis trifoliolata*, (B) *Ephedra aspera*, (C) *Leucophyllum laevigatum*, (D) *Lindleya mespiloides*, (E) *Setchellanthus caeruleus* and (F) the average of all species. Dark gray shaded areas represent suitable areas for the distribution of each species based on the projection of ecological niche models onto future climate scenarios. Black dots represent species populations. Light gray shaded areas in (F) indicate protected natural areas.

Haplotype and nucleotide diversity for every population of each species is included in Table 1 and displayed in Figs. 2A–2F. The *B. trifoliolata* population with the highest haplotype diversity (*h*) was located in the Mapimiam subprovince in La Gavia and Parras (Fig. 2A); in *Leucophyllum laevigatum* it was in the Saladan and Mapimiam subprovinces (Fig. 2C); *S. caeruleus* in the Tehuacán Valley (Fig. 2E); *E. aspera* in the Mapimian subprovince; and *Lindleya mespiloides* in the Saladan subprovince (Figs. 2B and 2D). The nucleotide diversity π of *B. trifoliolata* coincides with the same localities mentioned above and in the Texas population; for *Leucophyllum laevigatum* nucleotide diversity was highest in Parral and Rocamontes Durango; *S. caeruleus* in Tehuacán, *E. aspera* in Galeana; and *Lindleya mespiloides* in Hojuelas, Durango (Fig. 2D). Among the populations of all of the species, *L. laevigatus* and *S. caeruleus* populations had the highest haplotype diversity. For *E. aspera, B. trifoliata*, *Leucophyllum laevigatum* and *Lindleya mespiloides* the highest haplotype diversity occurred in the Mapimian subprovince (Figs. 2A–2D), and for *S. caeruleus* in Tehuacán (Fig. 2E). Genetic landscape analysis revealed high haplotype diversity for all species in the northwestern areas of the Chihuahuan Desert with the exception of *S. caeruleus* for which haplotype diversity was high in the Tehuacán Valley (Fig. 2F). Average haplotype diversity was high along the mountains in localities in Texas, the Sierra Madre Oriental and the Tehuacán Valley (Fig. 2F). Table 3 includes *Fst* estimations for tudied species with their corresponding *P*-values, indicating that *S. caeruleus* has the highest values with the highest degree of differentiation among populations (Table S3 includes *Fst* pairwise estimations for populations of every species).

**Table 3:** Fixation index *Fst* for populations of the five studied species. Number of populations, and variance components among populations and within populations is indicated.

Source of variation		*Berberis trifoliata*	*Ephedra compacta*	*Leucophyllum laevigatus*	*Lindleya mespiloides*	*Setchellanthus caurelus*
Sample populations		20	9	9	20	4
Among populations	d.f	19	8	8	19	3
	Sum of squares	101.485	62.021	70.591	207.293	35.833
	Variance components	1.80299 Va	1.95550 Va	1.73775 Va	1.33925 Va	2.20357 Va
	Percentaje of variation	55.05	94.80	64.63	64.59	84.57
Within populations	d.f	24	27	32	133	17
	Sum of squares	35.33	2.895	30.433	97.661	6.833
	Variance components	1.47222 Vb	0.10723 Vb	0.95104 Vb	0.73429 Vb	0.40196 Vb
	Percentaje of variation	44.95	5.20	35.37	35.41	15.43
Total	d.f	43	35	40	152	20
	Sum of squares	136.818	64.917	101.024	304.954	42.667
	Variance components	3.27521	2.06273	2.68879	2.07354	2.60553
	Percentaje of variation	20	42.667	2.60553		
Fixation index (*Fst*)		0.55049***	0.94801***	0.64629***	0.64587***	0.84573***

**Note:**

*** Significant values at *P* < 0.000, significance tests (1,000 permutations).

### Ecological niche modeling

**Figure 3:**
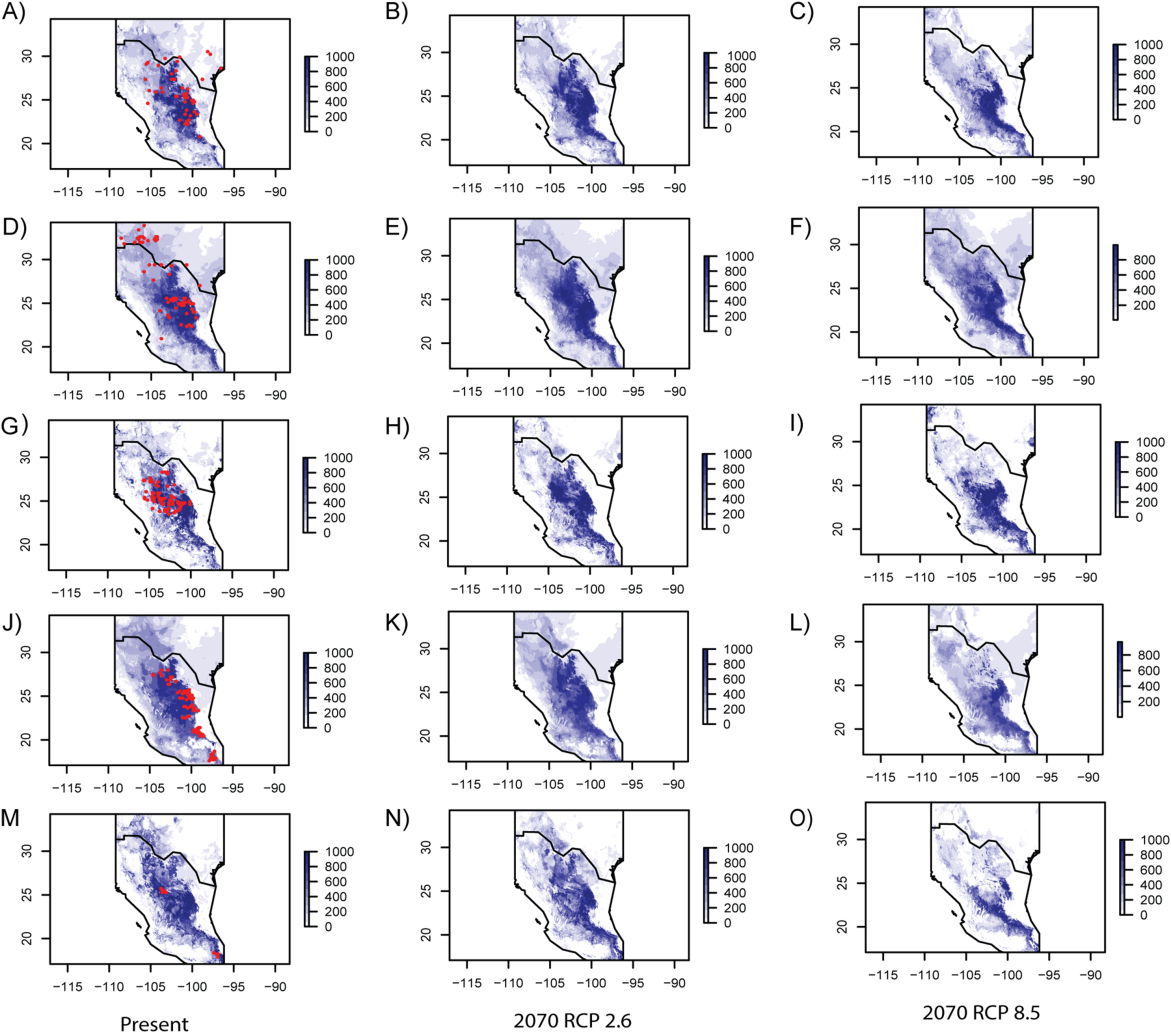
Ecological niche models for every studied species. Geographic projections of inferred ecological niche models from the species studied for present and future RCP 2.6 and RCP 8.5 climate scenarios. (A–C) *Berberis trifoliolata*. (C–E) *Ephedra aspera*. (G–I) *Leucophyllum laevigatum*. (J–L) *Lindleya mespiloides*. (M–O) *Setchellanthus caeruleus*.

Model performance evaluated with the ROC is summarized in Table 4, showing similar values for every studied species. Table 5 includes eigenvalues retrieved by the PCA that were utilized to generate SMDs (individual eigenvalues for every species are presented in Table S4). In general, current ENMs accurately predicted the known distribution of every species (Fig. 3), though the models over-predicted ranges of *E. aspera, Lindleya mespiloides* and *S. caeruleus* (Fig. 3). Future ENM with RCP2.6 and RCP8.5 models predicted extended ranges for all studied species, although RCP8.5 predicted less considerable areas (Fig. 3). Suitable conditions predicted by current and future climate scenarios were identified in the Mexican Plateau, in the areas corresponding to the Mapimian and Saladan subprovinces as well as in the Sierra Madre Oriental. Results with CCSM4 and MIROC-ESM climate scenarios were congruent with RCP2.6 and RCP8.5, respectively (included in Fig. S2).

**Table 4:** Model performance metrics for SDMs using the set of uncorrelated bioclimatic variables. Results are averages of 10 replicates per species using the 30% of occurrence data to test model performance.

Species	Mean Kappa	Mean TSS	Mean ROC
*Berberis trifoliolata*	0.5241	0.5822	0.8429
*Ephedra aspera*	0.4821	0.5575	0.8277
*Leucophyllum laevigatum*	0.4771	0.553	0.8252
*Lindleya mespiloides*	0.5267	0.5973	0.8461
*Setchellanthus caeruleus*	0.4823	0.5622	0.8215

**Table 5:** Percentage of variation explained by the 19 PC axes generated using the 19 bioclimatic variables for all the climatic scenarios used in this study.

PC axes	Eigenvalues	Variation explained	Cumulative variation explained
**PC1**	**2.688096938**	**0.380308692**	**0.380308692**
**PC2**	**2.257095915**	**0.26813063**	**0.648439322**
**PC3**	**1.981416205**	**0.206632115**	**0.855071437**
**PC4**	**1.145265806**	**0.069033356**	**0.924104793**
**PC5**	**0.761084738**	**0.030486841**	**0.954591634**
**PC6**	**0.54490501**	**0.015627446**	**0.97021908**
PC7	0.498986854	0.013104625	0.983323705
PC8	0.328036549	0.005663578	0.988987283
PC9	0.268050342	0.003781631	0.992768913
PC10	0.237464238	0.002967856	0.99573677
PC11	0.16446206	0.001423567	0.997160336
PC12	0.135499196	0.000966317	0.998126654
PC13	0.103072748	0.000559157	0.998685811
PC14	0.101441214	0.000541596	0.999227407
PC15	0.089397327	0.000420625	0.999648032
PC16	0.073675779	0.000285691	0.999933723
PC17	0.032814995	5.67E-05	0.999990398
PC18	0.012624394	8.39E-06	0.999998786
PC19	0.004802651	1.21E-06	1

**Note:**

Rows in bold represent the variables used to generate raster layers to estimate SDMs and perform projection on geographic space.

### Current and future environmental resistance

**Figure 4:**
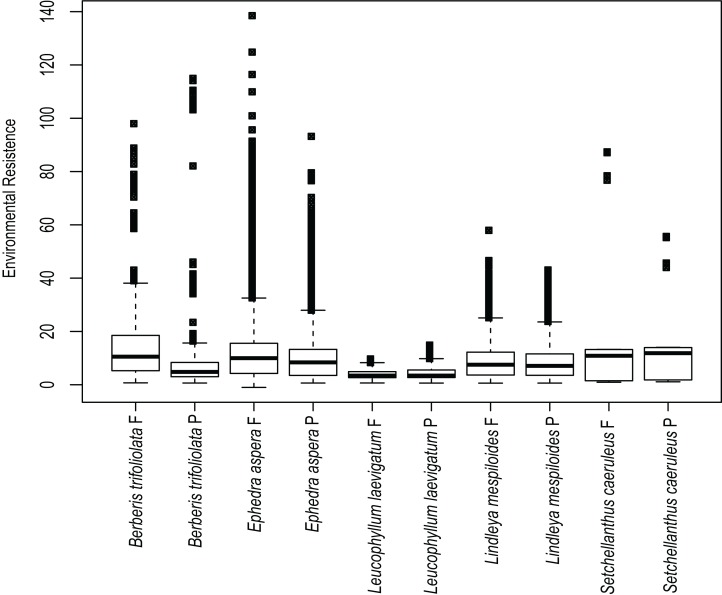
Environmental resistances for every species studied. Environmental resistance of the five studied species calculated in Circuitscape based on the suitability file derived from the ecological niche modeling of present (P) and future (F) climate scenarios, averaged for each taxon and displayed in boxplots.

Estimates of environmental resistance based on the prediction of current ecological niche models are displayed in Fig. 4. The most important temperature and precipitation variables were assigned resistance costs ranging from zero (no resistance to movement) to 100 (strong barrier to movement). *B. trifoliolata* and *E. aspera* had the most elevated changes in environmental resistance under present and future climate scenarios with a predicted increase in environmental resistance that would lead to lower gene flow (Fig. 4). The other species have a similar degree of environmental resistance under current and future climate scenarios (Fig. 4).

### Protected areas and genetic diversity

**Figure 5:**
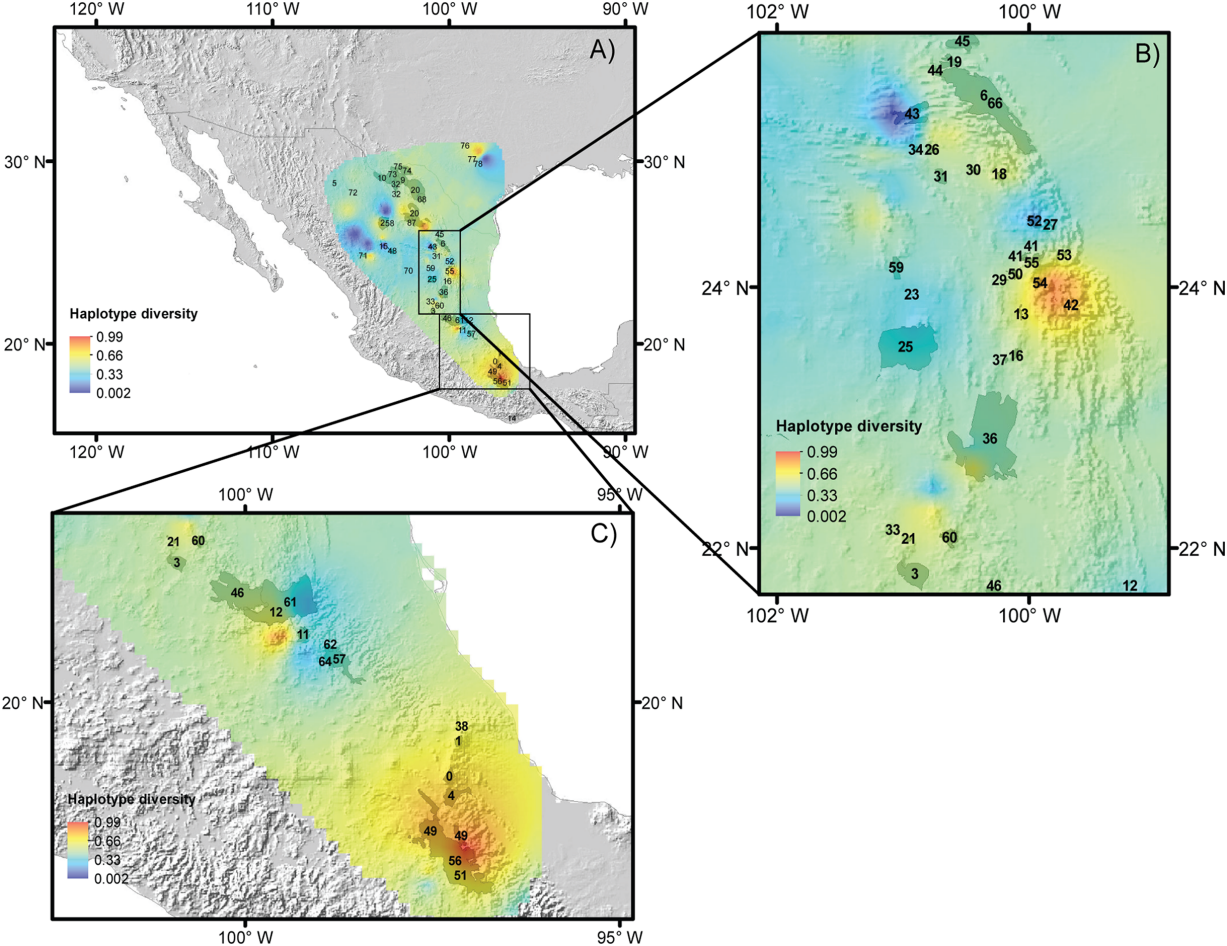
Reserves and haplotype diversity. Haplotype diversity of the five species studied overlaid onto the map of reserves and protected natural areas. Protected natural areas follow the designations of the Mexican Commission of Protected Natural Areas (CONANP, www.gob.mx/conanp) and the Texas State Parks and Wildlife Department (https://tpwd.texas.gov/state-parks/). (A) Map with all protected areas. (B) Detail of areas in the central region of the Chihuahuan Desert. (C) Detail of the southernmost areas of the Chihuahuan Desert. See Table 2 for numbers and the full names of these areas.

For the five species, most of the populations with high genetic diversity are located in unprotected areas, with the exception of the Tehuacán Valley Biosphere Reserve, and the southernmost area of the Chihuahuan Desert (Figs. 5A–5C, protected areas 49, 51 and 56, see Table 2 for information on protected areas, their designation and their surface area). In the central area of the Chihuahuan Desert, populations with high genetic diversity lie between two protected areas (Fig. 5, protected area 11 Los Mármoles and 12 Sierra Gorda). In the central-eastern part of the desert there is a high diversity of populations in an area specifically protected for wild wolves, not for the biota in general (Fig. 5, 55 Protected area for the Mexican wolf Zamora). In summary, two zones in the Chihuahuan Desert have the greatest haplotype diversity: the southern region of the Sierra Madre Oriental (between protected areas 42 Sierra Santa Marta de Abajo and 54 Zona de Restauración Ecológica del Lobo Mexicano Potrero de Zamora, Fig. 5B), and the Tehuacán Valley (around protected areas 49 and 56, which correspond to the Tehuacán-Cuicatlán Biosphere Reserve, see Fig. 5C).

## Discussion

### Genetic diversity and its spatial distribution

In plants, higher genetic diversity has been frequently found in zones that had a stable climate during glacial periods and for which post-glacial migration was identified (Faye et al., 2016; Wolfe et al., 2016), and in contrast, unstable regions are expected to represent recently colonized areas and thus exhibit lower genetic diversity (Abellán & Svenning, 2014; Caze et al., 2016; Ornelas, Licona-Vera & Vásquez-Aguilar, 2018). Higher genetic diversity has been found as well in environments that fluctuate in time or space, in which different genotypes can be favored at different times or locations and shifting selection can support higher genetic variation in fitness, even when stabilizing selection is acting to reduce genetic variation (Nadeau, Urban & Bridle, 2017). In particular for the Chihuahuan Desert southern areas such like the Mezquital and the Tehuacán Valleys, have been proposed as areas of refugia for the biota of this warm desert because they were isolated during periods of glacial-interglacial cycles, with semi-arid climates and it has been discovered that a number of plants from these areas have high genetic diversity (Loera, Ickert-Bond & Sosa, 2017).

Results here coincide with these findings: (1) high nucleotide diversity was found for populations of *Lindleya mespiloides* and *S. caeruleus* in the southernmost limits of the Chihuahuan Desert areas that have been proposed as refugia; (2) with the exception of *S. caeruleus* the other species studied had high nucleotide diversity in several areas of the Mexican Plateau that had a stable climate during glacial periods, and moreover this species has the highest population differentiation; (3) the average of high haplotype diversity values for the five species along the Sierra Madre Oriental mountain range coincided with those expected for fluctuating environments.

In addition, results here agree with the indicators of genetic variation identified for various plant species distributed in the Chihuahuan Desert, such as *Fouquieria shrevei* (Aguirre-Liguori, Scheinvar & Eguiarte, 2014), *Agave striata* (Trejo, Alvarado-Cárdenas & Scheinvar, 2016), and *A. lechuguilla* (Scheinvar, Gámez & Castellanos-Morales, 2017), in which high genetic diversity was identified in the north coinciding with findings for *E. aspera* and *Leucophyllum laevigatum*. Likewise for *Hunnemannia fumariifolia*, high genetic variation was associated with areas in the vicinity of the Sierra Madre Oriental (Sosa, Ruiz-Sanchez & Rodríguez-Gómez, 2009), coinciding with results for *B. trifoliata* and *Leucophyllum laevigatum*.

### Ecological niche modeling and ecological resistance

Forecast showed that global warming would favor an expansion mostly in the western range for the five studied species, under the two models RCP2.6 and RCP8.5 (Fig. 3). RCP 2.6 and RCP 8.5 are sets of predictions that exclusively consider the components of radiative forcing, forecasting how energy is transmitted between grids using the laws of thermodynamics, allowing to estimate dozens of environmental variables (Moss et al., 2010). The RCP 2.6 scenario is the best case for limiting anthropogenic climate change in which global CO_2_ emission peak by 2020 and decline around 2080. The RCP 8.5 scenario is the worst because it considers that emission continue to increase rapidly and by 2,100 will stabilize. This scenario is highly energy intensive continuing to grow until the century reaching approximately three times current levels. Even though the latter model is based on higher emissions projecting more elevated future temperature, the ecological modeling did not predict marked differences in range of the studied species.

Results show that ecological resistance for the majority of species is similar in present and future conditions with the exception of *B. trifoliolata* and *E. aspera* where the ecological resistance will increase in future conditions. Ecological resistance can reduce functional connectivity and gene flow between populations (McRae & Beier, 2007; Spear et al., 2010; Peterman et al., 2014) and might affect genetic structure. Likewise, lower resistance leads to higher dispersal for individuals (Cushman et al., 2006). Thus the rest of species might be affected in connectivity among populations in future scenarios of climate change.

### Protected areas, genetic diversity and ecological resistance

On one hand, designing biodiversity conservation strategies should maximize genetic diversity and preserve adaptive potential (Polfus et al., 2016). On the other hand, identifying and protecting climate-change refugia is a good approach for conservation, particularly in areas of complex topography (Brito et al., 2016; Corlett & Westcott, 2013). Furthermore, mountain ranges constitute important centers of diversification in arid regions and act as species pumps into surrounding areas, in addition to being important reservoirs of cryptic diversity, even of common and widespread species (García-Porta et al., 2017).

Thus, we propose the preservation of two areas with high haplotype diversity, identified by ecological niche modeling in the Mexican Plateau and on the slopes of the Sierra Madre Oriental mountain range that will have suitable climate conditions in the future (these areas are indicated in Fig. 5). Despite the apparently large number of reserves in the Mexican system of natural areas that are located in the Chihuahuan Desert, only four large biosphere reserves are well managed: Barranca de Meztitlán, Mapimí, Sierra Gorda and Tehuacán-Cuicatlán. The others fall under a different designation, and some of them are small and not actively protected.

## Conclusions

In summary, this study shows that even shrubby species adapted to arid conditions will be affected by climate change; the five species will have suitable climate conditions for expanding their distribution to the west of the Chihuahuan Desert according to the future climate scenarios. However, for species with low ecological resistance, climate change can have the effect of allowing gene flow among populations. Based on suitability of habitat and haplotype diversity, we suggest preserving two hotspots of genetic diversity in the Sierra Madre Oriental, located in areas without protection.

Innovative conservation measures are being currently proposed such like moveable and temporary reserves as well as targeted gene flow (Reside, Butt & Adams, 2018). Resilient species to climate change are crucial for understanding genomics of climate change, assessing genes involved with the response to their future environment in view of climate change (Rellstab et al., 2016). We suggest these measures of moveable reserves and genomics of climate change should be implemented in the Chihuahuan Desert plants.

Understanding the effect of climate change in plants of the Chihuahuan Desert is crucial: (1) dry tropical areas are extremely understudied compared to the humid biomes (Muenchow et al., 2018); (2) species in deserts are preserved evidence of humid past. In particular the woody elements of the warm Chihuahuan Desert formed part of the Madro-Tertiary Geoglora, a fossil flora from the southwestern United States and North of Mexico so desert species are outstanding repositories of past climate changes; (3) the most decisive climate variables in deserts are related to temperature and precipitation, understanding future changes in both gives insights whether plants will be capable of adaptation to changes in these climate variables.

## Supplemental Information

10.7717/peerj.6572/supp-1 Supplemental Information 1 GenBank accessions and newly generated sequences. Table S1. List of haplotypes and GenBank accession for populations studied. The newly generated sequences for *Ephedra compacta* and *Leucophyllum laevigatum* are fully included.

10.7717/peerj.6572/supp-2 Supplemental Information 2 Georeferences utilized for ecological niche modeling.

10.7717/peerj.6572/supp-3 Supplemental Information 3 Table S3. Fst estimations for every studied species. Population numbers correspond to localities included in Table 1.

10.7717/peerj.6572/supp-4 Supplemental Information 4 Loadings of single bioclimatic variables for the first six PC axes.

10.7717/peerj.6572/supp-5 Supplemental Information 5 PCA graphs of bioclimate variables for studied species. PCA graphs displaying contribution of climate variables for niche space for every species studied. a) *Berberis trifoliolata*. b) *Ephedra compacta*. c) *Leucophyllum laevigatum*. d) *Lindleya mespiloides*. d) *Setchellanthus caeruleus*.

10.7717/peerj.6572/supp-6 Supplemental Information 6 CCSM4 AND MIROC-ESM niche models. Geographic projections of inferred ecological niche models from the species studied for present and future GCMs climate scenarios CCSM4 and MIROC-ESM.

## Additional Information and Declarations

### Field Study Permissions

The following information was supplied relating to field study approvals (i.e., approving body and any reference numbers): We obtained collecting permits to conduct this work from the Secretaría de Medio Ambiente y Recursos Naturales, Instituto Nacional de Ecología, Dirección General de Vida Silvestre (permit number: Registro de Colección Científica VER-FLO-228-09-09 SEMARNAT).

### Data Availability

The following information was supplied regarding data availability: GenBank accession numbers and newly generated sequences are available in Table S1, and the georeferences are included in the Supplementary Material.
