# Supplementary material for: Climate change and conservation in a warm North American desert: effect in shrubby plants
Source: PeerJ. 2019 Mar 7;7:e6572. doi: 10.7717/peerj.6572 (PMC6409089; doi:10.7717/peerj.6572)
Supplement: Supplemental Information 1 — Table S1. List of haplotypes and GenBank accession for populations studied. The newly generated sequences for Ephedra compacta and Leucophyllum laevigatum are fully included. [file peerj-07-6572-s001.pdf]

Table S1. List of haplotypes and GenBank accession for populations studied. The newly generated sequences for *Ephedra compacta* and *Leucophyllum laevigatum* are fully included.

	Berberis trifoliolata			***Setchellanthus caeruleus***				***Lindleya mespiloides***		
	Haplotype	*rpl32-trnL*	*trnH-psbA*	Haplotype	*psbA-trnH*	*trnF-trnL*	*psbI-psbK*	Haplotype	*psbA-trnH*	*trnH-rpl2*
México, Acatitlán, Qro (ACT)								H4, H8	KX467735<br>KX467739	KX467769
Mexico, Arteaga, Coah (ART)	H8, H13, H14	KY397705<br>KY397689<br>KY397680	KY397750<br>KY397734<br>KY397725							
Mexico, borderline Coah-Zac (FCZ)	H14, H15	KY397680<br>KY397717	KY397725<br>KY397761					H1, H3	KX467732<br>KX467734	KX467768<br>KX467769
Mexico, Cerro El Potosí, NL (CEP)	H16, H36, H40	KY397679<br>KY397684<br>KY397762	KY397724<br>KY397729<br>KY397730					H1, H2, H5, H7	KX467732<br>KX467733<br>KX467736<br>KX467738	KX467768<br>KX467770
Mexico, Chih (CHIH)	H2, H27, H31, H32	KY397708<br>KY397695<br>KY397715<br>KY397716	KY397762<br>KY397740<br>KY397759<br>KY397716							
Mexico, Cuatro Cienegas, Coah (CC)	H2, H21, H22, H23, H24, H25, H26, H27	KY397708<br>KY397690<br>KY397713<br>KY397712<br>KY397710<br>KY397711<br>KY397714<br>KY397695	KY397762<br>KY397735<br>KY397757<br>KY397756<br>KY397754<br>KY397755<br>KY397758<br>KY397740							
México, Galeana, NL (GAL)										
Mexico, Guadalcazar, SLP (GUAD)	H34	KY397702	KY397747					H1, H26, H27, H28	KX467732<br>KX467757<br>KX467758<br>KX467759	KX467772

Mexico, Ixmiquilpan, Hgo (IXM)	H1, H2	KY397707 KY397708	KY397752 KY397762							
México, Jicotlán, Oax (JIC)								H11, H12	KX467742 KX467743	KX467767 KX467772
México, La Angostura, Coah (ANG)	H2, H13, H14	KY397708 KY397689 KY397680	KY397762 KY397734 KY397725							
Mexico, La Gavia, Coah (LG)	H3, H4, H5, H13, H21	KY397687 KY397686 KY397688 KY397689 KY397690	KY397732 KY397731 KY397733 KY397734 KY397735							
México, La Lagunita, Qro (LAG)								H8, H20, H21, H22	KX467739 KX467751 KX467752 KX467753	KX467769
Mexico, La Zarca, Dgo (ZAR)										
México, Laguna Seca, SLP (LSE)								H11, H14	KX467742 KX467745	KX467767
Mexico, Lerdo, Dgo (LER)				H9, H10	KC778733 KC778734 KC778735 KC778736	KC778769 KC778770 KC778771 KC778772	KC778749 KC778750 KC778751 KC778752			
México, Maconí, Qro (MAC)								H3, H8, H22, H23	KX467734 KX467739 KX467753 KX467754	KX467771
Mexico, Mapimí, Dgo (MAP)				H8	KC778733 KC778734 KC778735 KC778736	KC778769 KC778770 KC778771 KC778772	KC867712 KC867713 KC867714 KC867715			
Mexico, Matehuala SLP (MAT)	H4, H6, H13, H20	KY397686 KY397704 KY397689 KY397703	KY397731 KY397749 KY397734 KY397748							

Mexico, Ojinaga, Chih (OJIN)	H27, H28, H30	KY397695<br>KY397698<br>KY397699	KY397740<br>KY397743<br>KY397744							
Mexico, Ojuelas, Dgo (OJU)	H27, H29	KY397695<br>KY397697	KY397740<br>KY397742					H4, H5, H6	KX467735<br>KX467736<br>KX467737	KX467768<br>KX467770
Mexico, Pablillo, NL (PAB)	H37	KY397718	KY397762							
Mexico, Parral, Chih (PARRAL)	H27, H33, H35	KY397695<br>KY397696<br>KY397694	KY397740<br>KY397741<br>KY397739							
Mexico, Parras, Coah (PARR)	H14, H16, H17, H18, H19	KY397680<br>KY397679<br>KY397682<br>KY397681<br>KY397683	KY397725<br>KY397724<br>KY397727<br>KY397726<br>KY397728					H1, H2	KX467732<br>KX467733	KX467767
México, Peña Miller, Qro (MIL)								H22, H24, H25	KX467753<br>KX467755<br>KX467756	KX467769
México, Ramos Arizpe, Coah (RAZ)	H12, H13	KY397691<br>KY397689	KY397736<br>KY397734							
Mexico, Rancho Jaguey, Coah (RJ)	H14	KY397680	KY397725							
Mexico, Real de Catorce SLP (RC)	H2, H4, H41, H42, H43, H44, H45	KY397708<br>KY397686<br>KY397721<br>KY397720<br>KY397719<br>KY397723<br>KY397722	KY397762<br>KY397731<br>KY397765<br>KY397764<br>KY397763<br>KY397767<br>KY397766					H11, H29, H30	KX467742<br>KX467760<br>KX467761	KX467769
Mexico, Rocamontes, Dgo (ROCA)	H7, H13	KY397709<br>KY397689	KY397753<br>KY397734					H1, H2, H31	KX467732<br>KX467733<br>KX467762	KX467768
Mexico, Rodeo, Dgo (ROD)										

Mexico, San Juan del Rio, Dgo (ATO)										
Mexico, San Pedro Iturbide, NL (SPI)	H38, H39	KY397693<br>KY397692	KY397738<br>KY397737					H2, H7	KX467733<br>KX467738	KX467768<br>KX467770
Mexico, Santa María del Oro, Dgo (StaMO)	H27	KY397695	KY397740							
México, Santa Teresa, SLP (TER)								H11	KX467742	KX467767<br>KX467769
Mexico, Sierra de San Miguel, NL (SSM)	H14, H16	KY397680<br>KY397679	KY397725<br>KY397724							
México, Sierra Mojada, Coah (MOJ)										
México, Tamazulapam, Oax (TAM)								H11, H13, H14	KX467742<br>KX467744<br>KX467745	KX467772
Mexico, Teotitlán, Oax (TEO)				H3, H7	KC778722<br>KC778723<br>KC778724<br>KC778725<br>KC778726	KC778758<br>KC778759<br>KC778760<br>KC778761<br>KC778762	KC778738<br>KC778739<br>KC778740<br>KC778741<br>KC778742<br>KC778743			
México, Teotongo, Oax (TT)								H4, H11, H12, H15	KX467735<br>KX467742<br>KX467743<br>KX467746	KX467767<br>KX467771<br>KX467772
Mexico, Trópico de Cáncer, Zac (TCA)								H7	KX467738	KX467770
Mexico, Ventura, SLP (VEN)	H13, H34	KY397689<br>KY397702	KY397734<br>KY397747							

Mexico, Zapotitlán de Salinas Pue (TEH)				H1 H2, H3, H4, H5, H6	KC778727 KC778728 KC778729 KC778730	KC778763 KC778764 KC778765 KC778766 KC778767	KC778744 KC778745 KC778746 KC778747 KC778748	H16, H17, H18, H19	KX467747 KX467748 KX467749 KX467750	KX467767 KX467771 KX467772
México, Zaragoza, NL (ZAG)								H8, H9, H10	KX467739 KX467740 KX467741	KX467769 KX467772
U.S. Austin, Texas (AUST)	H9, H13	KY397706 KY397689	KY397751 KY397734							
U.S. Purola, Texas (PUR)	H2, H10, H11, H13	KY397708 KY397701 KY397700 KY397691 KY397689	KY397762 KY397746 KY397745 KY397736 KY397734							

Sequences

> *Ephedra compacta* 1 (matK). Mexico, Arteaga, Coah (ART). Sierra_Art_1
ATTCTTTCTTTTGAAAGTACTATTTGGTTTATTCGGTTCAAGCTAGATTGTACTATGTGTTATTTTATTTCTAAAAAGAAAGGTTTAG
GAGGTTTTTTCTTGAAAAAAAATGAAAACATACGTTGGCAACAACATTTTTTATATCCCCTCTTATTCCATAACGATTTCTATGTTA
TAGATCCGAATCTGTTATTCAACTCAAGCCCTTCTTTCGAAAAAATAGAAAAATTACCTAATAGTTTCCGTTTTTTGAATGTAAAAC
GTTCAATTAAGCTATTACATCAACAAAACTATCTTGTGTATAATACAAGAAGTTCTTGTTTTAATTTCATTGGAAAAACTTTTTTTTT
CTGTTTTGATATTTTTGTTTCCACGTGGTGGAAACACTTTTTTGGTTTCAAAGTAACTTTCAAAGAAATAAATCAACAGGTCAATTTT
CAATCAGTCTTTTCTCTATTTCTTTTTTTGGAAGAAGTCTTTATGTTTTCTCTTTCTTTTTCTAATATAAGAATACCCTCTTCGATTCA
TTCAGAGCTGTTAATTAGACGTTTCAAATTTTCGATTCAAGATGTTTCTTTTTTACATTTTTTAAGTTTTATACTTTTTTCAAAGCAAT
TTAAGTTTGTGAATAATTCTATTATTTTTCCAAAAGGAAGTGTGATTTTCTGTTTCTTATTAGGGAATATTCTTCTTTCTATTTTCGAA
GATTTTTTCACTCTTCGATGGAAAAGTTGTTTTCATGAAAAATCATTGTCTTATGGTCTTTTTTCAGAACAAAAGCATTTTCAACAA
AAATGGAATTTTTTAACCCGAAAACCGAAAAAAAAAGACACGATAATATTGCTAAAGGATTTTTTTTTTCACTATATAAGATATGG
GGAAAAATTGATTTTGCTGGGAACTACTATTCTAGTCAAAAAATGTGAATTTTTTTTCTTAAATTTTTGGCAAACTTATCTTTTTGTT
TTATCGGAACCCTCTAGTTTTTTTTTGAAACAAATTTCCAGTCAAAATATGTTTTTTCTAGCTTATTACTTAGAATATCCAACAAACT
CTTTTTTACTCCGACTAAATATCTTAGATTATTTTCTATCTACTGATTTTGTTAGCAGGGAATTAAATTCAAAACTTAGCGCTGTTTT
TGTTATTCAATTTTTATCAAAAGAAGGATTATGTGATATAATGGGTAACCCGAAGAGTAAATTAGCATGGCTTAGTTTTACCGACA
ATTCTATTCTTGATAAATATGA
> *Ephedra compacta* 2 (matK). Mexico, Arteaga, Coah (ART). Sierra_Art_2
ATTCTTTCTTTTGAAAGTACTATTTGGTTTATTCGGTTCAAGCTAGATTGTACTATGTGTTATTTTATTTCTAAAAAGAAAGGTTTAG
GAGGTTTTTTCTTGAAAAAAAATGAAAACATACGTTGGCAACAACATTTTTTATATCCCCTCTTATTCCATAACGATTTCTATGTTA
TAGATCCGAATCTGTTATTCAACTCAAGCCCTTCTTTCGAAAAAATAGAAAAATTACCTAATAGTTTCCGTTTTTTGAATGTAAAAC
GTTCAATTAAGCTATTACATCAACAAAACTATCTTGTGTATAATACAAGAAGTTCTTGTTTTAATTTCATTGGAAAAACTTTTTTTTT
CTGTTTTGATATTTTTGTTTCCACGTGGTGGAAACACTTTTTTGGTTTCAAAGTAACTTTCAAAGAAATAAATCAACAGGTCAATTTT

CAATCAGTCTTTTCTCTATTTCTTTTTTTGGAAGAAGTCTTTATGTTTTCTCTTTCTTTTTCTAATATAAGAATACCCTCTTCGATTCA
TTCAGAGCTGTTAATTAGACGTTTCAAATTTTCGATTCAAGATGTTTCTTTTTTACATTTTTTAAGTTTATACTTTTTTCAAAGCAAT
TTAAGTTTGTGAATAATTCTATTATTTTTCCAAAAGGAAGTGTGATTTTCTGTTTCTTATTAGGGAATATTCTTCTTTCTATTTTCGAA
GATTTTTTCACTCTTCGATGGAAAAGTTGTTTTCATGAAAAATCATTGTCTTATGGTCTTTTTTCAGAACAAAAGCATTTTCAACAA
AAATGGAATTTTTTAACCCGAAAACCGAAAAAAAAAGACACGATAATATTGCTAAAGGATTTTTTTTTCACTATATAAGATATGG
GGAAAAATTGATTTGCTGGGAACTACTATTCTAGTCAAAAAATGTGAATTTTTTTTCTTAAATTTTGGCAAACTTATCTTTTTGTT
TTATCGGAACCCTCTAGTTTTTTTTTGAAACAAATTTCCAGTCAAAATATGTTTTTCTAGCTTATTACTTAGAATATCCAACAAACT
CTTTTTTACTCCGACTAAATATCTTAGATTATTTTCTATCTACTGATTTTGTTAGCAGGGAATTAAATTCAAAACTTAGCGCTGTTTT
TGTTATTCAATTTTTATCAAAAGAAGGATTATGTGATATAATGGGTAACCCGAAGAGTAAATTAGCATGGCTTAGTTTTACCGACA
ATTCTATTCTTGATAAATATGA
> *Ephedra compacta* 4 (matK). Mexico, Arteaga, Coah (ART). Sierra_Art_4
ATTCTTTCTTTTGAAAGTACTATTTGGTTTATTCGGTTCAAGCTAGATTGTACTATGTGTTATTTTATTTCTAAAAAGAAAGGTTTAG
GAGGTTTTTTCTTGAAAAAAAAATGAAAACATACGTTGGCAACAACATTTTTTATATCCCCTCTTATTCCATAACGATTTCTATGTTA
TAGATCCGAATCTGTTATTCAACTCAAGCCCTTCTTTCGAAAAAATAGAAAAATTACCTAATAGTTTCCGTTTTTTGAATGTAAAAC
GTTCAATTAAGCTATTACATCAACAAAACTATCTTGTGTATAATACAAGAAGTTCTTGTTTTAATTTCATTGGAAAAACTTTTTTTTT
CTGTTTTGATATTTTTGTTTCCACGTGGTGGAAACACTTTTTTGGTTTCAAAGTAACTTTCAAAGAAATAAATCAACAGGTCAATTTT
CAATCAGTCTTTTCTCTATTTCTTTTTTTGGAAGAAGTCTTTATGTTTTCTCTTTCTTTTTCTAATATAAGAATACCCTCTTCGATTCA
TTCAGAGCTGTTAATTAGACGTTTCAAATTTTCGATTCAAGATGTTTCTTTTTTACATTTTTTAAGTTTATACTTTTTTCAAAGCAAT
TTAAGTTTGTGAATAATTCTATTATTTTTCCAAAAGGAAGTGTGATTTTCTGTTTCTTATTAGGGAATATTCTTCTTTCTATTTTCGAA
GATTTTTTCACTCTTCGATGGAAAAGTTGTTTTCATGAAAAATCATTGTCTTATGGTCTTTTTTCAGAACAAAAGCATTTTCAACAA
AAATGGAATTTTTTAACCCGAAAACCGAAAAAAAAAGACACGATAATATTGCTAAAGGATTTTTTTTTCACTATATAAGATATGG
GGAAAAATTGATTTGCTGGGAACTACTATTCTAGTCAAAAAATGTGAATTTTTTTTCTTAAATTTTGGCAAACTTATCTTTTTGTT
TTATCGGAACCCTCTAGTTTTTTTTTGAAACAAATTTCCAGTCAAAATATGTTTTTCTAGCTTATTACTTAGAATATCCAACAAACT
CTTTTTTACTCCGACTAAATATCTTAGATTATTTTCTATCTACTGATTTTGTTAGCAGGGAATTAAATTCAAAACTTAGCGCTGTTTT
TGTTATTCAATTTTTATCAAAAGAAGGATTATGTGATATAATGGGTAACCCGAAGAGTAAATTAGCATGGCTTAGTTTTACCGACA
ATTCTATTCTTGATAAATATGA
> *Ephedra compacta* 5 (matK). Mexico, Arteaga, Coah (ART). Sierra_Art_5
ATTCTTTCTTTTGAAAGTACTATTTGGTTTATTCGGTTCAAGCTAGATTGTACTATGTGTTATTTTATTTCTAAAAAGAAAGGTTTAG
GAGGTTTTTTCTTGAAAAAAAAATGAAAACATACGTTGGCAACAACATTTTTTATATCCCCTCTTATTCCATAACGATTTCTATGTTA
TAGATCCGAATCTGTTATTCAACTCAAGCCCTTCTTTCGAAAAAATAGAAAAATTACCTAATAGTTTCCGTTTTTTGAATGTAAAAC
GTTCAATTAAGCTATTACATCAACAAAACTATCTTGTGTATAATACAAGAAGTTCTTGTTTTAATTTCATTGGAAAAACTTTTTTTTT
CTGTTTTGATATTTTTGTTTCCACGTGGTGGAAACACTTTTTTGGTTTCAAAGTAACTTTCAAAGAAATAAATCAACAGGTCAATTTT
CAATCAGTCTTTTCTCTATTTCTTTTTTTGGAAGAAGTCTTTATGTTTTCTCTTTCTTTTTCTAATATAAGAATACCCTCTTCGATTCA
TTCAGAGCTGTTAATTAGACGTTTCAAATTTTCGATTCAAGATGTTTCTTTTTTACATTTTTTAAGTTTATACTTTTTTCAAAGCAAT
TTAAGTTTGTGAATAATTCTATTATTTTTCCAAAAGGAAGTGTGATTTTCTGTTTCTTATTAGGGAATATTCTTCTTTCTATTTTCGAA
GATTTTTTCACTCTTCGATGGAAAAGTTGTTTTCATGAAAAATCATTGTCTTATGGTCTTTTTTCAGAACAAAAGCATTTTCAACAA
AAATGGAATTTTTTAACCCGAAAACCGAAAAAAAAAGACACGATAATATTGCTAAAGGATTTTTTTTTCACTATATAAGATATGG
GGAAAAATTGATTTGCTGGGAACTACTATTCTAGTCAAAAAATGTGAATTTTTTTTCTTAAATTTTGGCAAACTTATCTTTTTGTT
TTATCGGAACCCTCTAGTTTTTTTTTGAAACAAATTTCCAGTCAAAATATGTTTTTCTAGCTTATTACTTAGAATATCCAACAAACT
CTTTTTTACTCCGACTAAATATCTTAGATTATTTTCTATCTACTGATTTTGTTAGCAGGGAATTAAATTCAAAACTTAGCGCTGTTTT
TGTTATTCAATTTTTATCAAAAGAAGGATTATGTGATATAATGGGTAACCCGAAGAGTAAATTAGCATGGCTTAGTTTTACCGACA
ATTCTATTCTTGATAAATATGA

> *Ephedra compacta* 6 (matK). Mexico, Arteaga, Coah (ART). Sierra_Art_6
ATTCTTTCTTTTGAAAGTACTATTTGGTTTATTCGGTTCAAGCTAGATTGTACTATGTGTTATTTATTTCTAAAAAGAAAGGTTTAG
GAGGTTTTTTCTTGAAAAAAAATGAAAACATACGTTGGCAACAACATTTTTTATATCCCCTCTTATTCCATAACGATTTCTATGTTA
TAGATCCGAATCTGTTATTCAACTCAAGCCCTTCTTTCGAAAAAATAGAAAAATTACCTAATAGTTTCCGTTTTTTGAATGTAAAAC
GTTCAATTAAGCTATTACATCAACAAAACTATCTTGTGTATAATACAAGAAGTTCTTGTTTTAATTTCATTGGAAAAACTTTTTTTT
CTGTTTTGATATTTTTGTTTCCACGTGGTGGAAACACTTTTTTGGTTTCAAAGTAACTTTCAAAGAAATAAATCAACAGGTCAATTTT
CAATCAGTCTTTTCTCTATTTCTTTTTTTGGAAGAAGTCTTTATGTTTTCTCTTTCTTTTTCTAATATAAGAATACCCTCTTCGATTCA
TTCAGAGCTGTTAATTAGACGTTTCAAATTTTCGATTCAAGATGTTTCTTTTTTACATTTTTTAAGTTTTATACTTTTTTCAAAGCAAT
TTAAGTTTGTGAATAATTCTATTATTTTTCCAAAAGGAAGTGTGATTTTCTGTTTCTTATTAGGGAATATTCTTCTTTCTATTTTCGAA
GATTTTTTCACTCTTCGATGGAAAAGTTGTTTTCATGAAAAATCATTGTCTTATGGTCTTTTTTCAGAACAAAAGCATTTTCAACAA
AAATGGAATTTTTTAACCCGAAAACCGAAAAAAAAAGACACGATAATATTGCTAAAGGATTTTTTTTTCACTATATAAGATATGG
GGAAAAATTGATTTTGCTGGGAACTACTATTCTAGTCAAAAAATGTGAATTTTTTTTCTTAAATTTTGGCAAACTTATCTTTTTGTT
TTATCGGAACCCTCTAGTTTTTTTTTGAAACAAATTTCCAGTCAAAATATGTTTTTTCTAGCTTATTACTTAGAATATCCAACAAACT
CTTTTTTACTCCGACTAAATATCTTAGATTATTTTCTATCTACTGATTTTGTTAGCAGGGAATTAAATTCAAAACTTAGCGCTGTTTT
TGTTATTCAATTTTTATCAAAAGAAGGATTATGTGATATAATGGGTAACCCGAAGAGTAAATTAGCATGGCTTAGTTTTACCGACA
ATTCTATTCTTGATAAATATGA

> *Ephedra compacta* 2 (matK). Mexico, borderline Coah-Zac (FCZ). Coah_Zac_2
ATTCTTTCTTTTGAAAGTACTATTTGGTTTATTCGGTTCAAGCTAGATTGTACTATGTGTTATTTATTTCTAAAAAGAAAGGTTTAG
GAGGTTTTTTCTTGAAAAAAAATGAAAACATACGTTGGCAACAACATTTTTTATATCCCCTCTTATTCCATAACGATTTCTATGTTA
TAGATCCGAATCTGTTATTCAACTCAAGCCCTTCTTTCGAAAAAATAGAAAAATTACCTAATAGTTTCCGTTTTTTGAATGTAAAAC
GTTCAATTAAGCTATTACATCAACAAAACTATCTTGTGTATAATACAAGAAGTTGTTGTTTTAATTTCATTGGAAAAACTTTTTTTT
CTGTTTTGATATTTTTGTTTCCACGTGGTGGAAACACTTTTTTGGTTTCAAAGTAACTTTCAAAGAAATAAATCAACAGGTCAATTTT
CAATCAGTCTTTTCTCTATTTCTTTTTTTGGAAGAAGTCTTTATGTTTTCTCTTTCTTTTTCTAATATAAGAATACCCTCTTCGATTCA
TTCAGAGCTGTTAATTAGACGTTTCAAATTTTCGATTCAAGATGTTTCTTTTTTACATTTTTTAAGTTTTATACTTTTTTCAAAGCAAT
TTAAGTTTGTGAATAATTCTATTATTTTTCCAAAAGGAAGTGTGGTTTTCTGTTTCTTATTAGGGAATATTCTTCTTTCTATTTTCGAA
GATTTTTTCACTCTTCGATGGAAAAGTTGTTTTCATGAAAAATCATTGTCTTATGGTCTTTTTTCAGAACAAAAGCATTTTCAACAA
AAATGGAATTTTTTAACCCGAAAACCGAAAAAAAAAGACACGATAATATTGCTAAAGGATTTTTTTTTCACTATATAAGATATGG
GGAAAAATTGATTTTGCTGGGAACTACTATTCTAGTCAAAAAATGTGAATTTTTTTTCTTCAATTTTGGCAAACTTATCTTTTTGTT
TTATCGGAACCCTCTAGTTTTTTTTTGAAACAAATTTCCAGTCAAAATATGTTTTTTCTAGCTTATTACTTAGAATATCCAACAAACT
CTTTTTTACTCCGACTAAATATCTTAGATTATTTTCTATCTACTGATTTTGTTAGCAGGGAATTAAATTCAAAACTTAGCGCTGTTTT
TGTTATTCAATTTTTATCAAAAGAAGGATTATGTGATATAATGGGTAACCCGAAGAGTAAATTAGCATGGCTTAGTTTTACCGACA
ATTCTATTCTTGATAAATATGA

> *Ephedra compacta* 3 (matK). Mexico, borderline Coah-Zac (FCZ). Coah_Zac_3
ATTCTTTCTTTTGAAAGTACTATTTGGTTTATTCGGTTCAAGCTAGATTGTACTATGTGTTATTTATTTCTAAAAAGAAAGGTTTAG
GAGGTTTTTTCTTGAAAAAAAATGAAAACATACGTTGGCAACAACATTTTTTATATCCCCTCTTATTCCATAACGATTTCTATGTTA
TAGATCCGAATCTGTTATTCAACTCAAGCCCTTCTTTCGAAAAAATAGAAAAATTACCTAATAGTTTCCGTTTTTTGAATGTAAAAC
GTTCAATTAAGCTATTACATCAACAAAACTATCTTGTGTATAATACAAGAAGTTGTTGTTTTAATTTCATTGGAAAAACTTTTTTTT
CTGTTTTGATATTTTTGTTTCCACGTGGTGGAAACACTTTTTTGGTTTCAAAGTAACTTTCAAAGAAATAAATCAACAGGTCAATTTT
CAATCAGTCTTTTCTCTATTTCTTTTTTTGGAAGAAGTCTTTATGTTTTCTCTTTCTTTTTCTAATATAAGAATACCCTCTTCGATTCA
TTCAGAGCTGTTAATTAGACGTTTCAAATTTTCGATTCAAGATGTTTCTTTTTTACATTTTTTAAGTTTTATACTTTTTTCAAAGCAAT
TTAAGTTTGTGAATAATTCTATTATTTTTCCAAAAGGAAGTGTGGTTTTCTGTTTCTTATTAGGGAATATTCTTCTTTCTATTTTCGAA
GATTTTTTCACTCTTCGATGGAAAAGTTGTTTTCATGAAAAATCATTGTCTTATGGTCTTTTTTCAGAACAAAAGCATTTTCAACAA

AAATGGAATTTTTTAACCCGAAAACCGAAAAAAAAAGACACGATAATATTGCTAAAGGATTTTTTTTTCACTATATAAGATATGG
GGAAAAATTGATTTGCTGGGAACTACTATTCTAGTCAAAAAATGTGAATTTTTTTTCTTCAATTTTGGCAAACTTATCTTTTTGTT
TTATCGGAACCCTCTAGTTTTTTTTTGAAACAAATTTCCAGTCAAAATATGTTTTTTCTAGCTTATTACTTAGAATATCCAACAAACT
CTTTTTTACTCCGACTAAATATCTTAGATTATTTTCTATCTACTGATTTTGTTAGCAGGGAATTAAATTCAAAACTTAGCGCTGTTTT
TGTTATTCAATTTTTATCAAAAGAAGGATTATGTGATATAATGGGTAACCCGAAGAGTAAATTAGCATGGCTTAGTTTTACCGACA
ATTCTATTCTTGATAAATATGA

> ***Ephedra compacta*** **4 (matK). Mexico, borderline Coah-Zac (FCZ).** Coah_Zac_4

ATTCTTTCTTTTGAAAGTACTATTTGGTTTATTCGGTTCAAGCTAGATTGTACTATGTGTTATTTTATTTCTAAAAAGAAAGGTTTAG
GAGGTTTTTTCTTGAAAAAAAAATGAAAACATACGTTGGCAACAACATTTTTTATATCCCCTCTTATTCCATAACGATTTCTATGTTA
TAGATCCGAATCTGTTATTCAACTCAAGCCCTTCTTTCGAAAAAATAGAAAAATTACCTAATAGTTTCCGTTTTTTGAATGTAAAAC
GTTCAATTAAGCTATTACATCAACAAAACTATCTTGTGTATAATACAAGAAGTTGTTGTTTTAATTTCATTGGAAAAACTTTTTTTTT
CTGTTTTGATATTTTTGTTTCCACGTGGTGGAAACACTTTTTTGGTTTCAAAGTAACTTTCAAAGAAATAAATCAACAGGTCAATTTT
CAATCAGTCTTTTCTCTATTTCTTTTTTTGGAAGAAGTCTTTATGTTTTCTCTTTCTTTTTCTAATATAAGAATACCCTCTTCGATTCA
TTCAGAGCTGTTAATTAGACGTTTCAAATTTTCGATTCAAGATGTTTCTTTTTTACATTTTTTAAGTTTTATACTTTTTTCAAAGCAAT
TTAAGTTTGTGAATAATTCTATTATTTTTCCAAAAGGAAGTGTGGTTTTCTGTTTCTTATTAGGGAATATTCTTCTTTCTATTTTCGAA
GATTTTTTCACTCTTCGATGGAAAAGTTGTTTTCATGAAAAATCATTGTCTTATGGTCTTTTTTCAGAACAAAAGCATTTTCAACAA
AAATGGAATTTTTTAACCCGAAAACCGAAAAAAAAAGACACGATAATATTGCTAAAGGATTTTTTTTTCACTATATAAGATATGG
GGAAAAATTGATTTGCTGGGAACTACTATTCTAGTCAAAAAATGTGAATTTTTTTTCTTCAATTTTGGCAAACTTATCTTTTTGTT
TTATCGGAACCCTCTAGTTTTTTTTTGAAACAAATTTCCAGTCAAAATATGTTTTTTCTAGCTTATTACTTAGAATATCCAACAAACT
CTTTTTTACTCCGACTAAATATCTTAGATTATTTTCTATCTACTGATTTTGTTAGCAGGGAATTAAATTCAAAACTTAGCGCTGTTTT
TGTTATTCAATTTTTATCAAAAGAAGGATTATGTGATATAATGGGTAACCCGAAGAGTAAATTAGCATGGCTTAGTTTTACCGACA
ATTCTATTCTTGATAAATATGA

> ***Ephedra compacta*** **5 (matK). Mexico, borderline Coah-Zac (FCZ).** Coah_Zac_5

ATTCTTTCTTTTGAAAGTACTATTTGGTTTATTCGGTTCAAGCTAGATTGTACTATGTGTTATTTTATTTCTAAAAAGAAAGGTTTAG
GAGGTTTTTTCTTGAAAAAAAAATGAAAACATACGTTGGCAACAACATTTTTTATATCCCCTCTTATTCCATAACGATTTCTATGTTA
TAGATCCGAATCTGTTATTCAACTCAAGCCCTTCTTTCGAAAAAATAGAAAAATTACCTAATAGTTTCCGTTTTTTGAATGTAAAAC
GTTCAATTAAGCTATTACATCAACAAAACTATCTTGTGTATAATACAAGAAGTTGTTGTTTTAATTTCATTGGAAAAACTTTTTTTTT
CTGTTTTGATATTTTTGTTTCCACGTGGTGGAAACACTTTTTTGGTTTCAAAGTAACTTTCAAAGAAATAAATCAACAGGTCAATTTT
CAATCAGTCTTTTCTCTATTTCTTTTTTTGGAAGAAGTCTTTATGTTTTCTCTTTCTTTTTCTAATATAAGAATACCCTCTTCGATTCA
TTCAGAGCTGTTAATTAGACGTTTCAAATTTTCGATTCAAGATGTTTCTTTTTTACATTTTTTAAGTTTTATACTTTTTTCAAAGCAAT
TTAAGTTTGTGAATAATTCTATTATTTTTCCAAAAGGAAGTGTGGTTTTCTGTTTCTTATTAGGGAATATTCTTCTTTCTATTTTCGAA
GATTTTTTCACTCTTCGATGGAAAAGTTGTTTTCATGAAAAATCATTGTCTTATGGTCTTTTTTCAGAACAAAAGCATTTTCAACAA
AAATGGAATTTTTTAACCCGAAAACCGAAAAAAAAAGACACGATAATATTGCTAAAGGATTTTTTTTTCACTATATAAGATATGG
GGAAAAATTGATTTGCTGGGAACTACTATTCTAGTCAAAAAATGTGAATTTTTTTTCTTCAATTTTGGCAAACTTATCTTTTTGTT
TTATCGGAACCCTCTAGTTTTTTTTTGAAACAAATTTCCAGTCAAAATATGTTTTTTCTAGCTTATTACTTAGAATATCCAACAAACT
CTTTTTTACTCCGACTAAATATCTTAGATTATTTTCTATCTACTGATTTTGTTAGCAGGGAATTAAATTCAAAACTTAGCGCTGTTTT
TGTTATTCAATTTTTATCAAAAGAAGGATTATGTGATATAATGGGTAACCCGAAGAGTAAATTAGCATGGCTTAGTTTTACCGACA
ATTCTATTCTTGATAAATATGA

> ***Ephedra compacta*** **6 (matK). Mexico, borderline Coah-Zac (FCZ).** Coah_Zac_6

ATTCTTTCTTTTGAAAGTACTATTTGGTTTATTCGGTTCAAGCTAGATTGTACTATGTGTTATTTTATTTCTAAAAAGAAAGGTTTAG
GAGGTTTTTTCTTGAAAAAAAAATGAAAACATACGTTGGCAACAACATTTTTTATATCCCCTCTTATTCCATAACGATTTCTATGTTA
TAGATCCGAATCTGTTATTCAACTCAAGCCCTTCTTTCGAAAAAATAGAAAAATTACCTAATAGTTTCCGTTTTTTGAATGTAAAAC

GTTCAATTAAGCTATTACATCAACAAAACTATCTTGTGTATAATACAAGAAGTTGTTGTTTTAATTTCATTGGAAAAACTTTTTTTT
CTGTTTTGATATTTTTGTTTCCACGTGGTGGAAACACTTTTTTGGTTTCAAAGTAACTTTCAAAGAAATAAATCAACAGGTCAATTTT
CAATCAGTCTTTTCTCTATTTCTTTTTTTGGAAGAAGTCTTTATGTTTTCTCTTTCTTTTTCTAATATAAGAATACCCTCTTCGATTCA
TTCAGAGCTGTTAATTAGACGTTTCAAATTTTCGATTCAAGATGTTTCTTTTTTACATTTTTTAAGTTTTATACTTTTTTCAAAGCAAT
TTAAGTTTGTGAATAATTCTATTATTTTTCCAAAAGGAAGTGTGGTTTTCTGTTTCTTATTAGGGAATATTCTTCTTTCTATTTTCGAA
GATTTTTTCACTCTTCGATGGAAAAGTTGTTTTCATGAAAAATCATTGTCTTATGGTCTTTTTTCAGAACAAAAGCATTTTCAACAA
AAATGGAATTTTTTAACCCGAAAACCGAAAAAAAAAGACACGATAATATTGCTAAAGGATTTTTTTTTCACTATATAAGATATGG
GGAAAAAATTGATTTTGCTGGGAACTACTATTCTAGTCAAAAAAATGTGAATTTTTTTTCTTCAATTTTTGGCAAACTTATCTTTTTGTT
TTATCGGAACCCTCTAGTTTTTTTTTGAAACAAATTTCCAGTCAAAATATGTTTTTTCTAGCTTATTACTTAGAATATCCAACAAACT
CTTTTTTACTCCGACTAAATATCTTAGATTATTTTCTATCTACTGATTTTGTTAGCAGGGAATTAAATTCAAAACTTAGCGCTGTTTT
TGTTATTCAATTTTTATCAAAAGAAGGATTATGTGATATAATGGGTAACCCGAAGAGTAAATTAGCATGGCTTAGTTTTACCGACA
ATTCTATTCTTGATAAATATGA

> *Ephedra compacta* 7 (matK). Mexico, borderline Coah-Zac (FCZ). Coah_Zac_7

ATTCTTTCTTTTGAAAGTACTATTTGGTTTATTCGGTTCAAGCTAGATTGTACTATGTGTTATTTTATTTCTAAAAAGAAAGGTTTAG
GAGGTTTTTTCTTGAAAAAAAAATGAAAACATACGTTGGCAACAACATTTTTTATATCCCCTCTTATTCCATAACGATTTCTATGTTA
TAGATCCGAATCTGTTATTCAACTCAAGCCCTTCTTTCGAAAAAATAGAAAAATTACCTAATAGTTTCCGTTTTTTGAATGTAAAAC
GTTCAATTAAGCTATTACATCAACAAAACTATCTTGTGTATAATACAAGAAGTTGTTGTTTTAATTTCATTGGAAAAACTTTTTTTT
CTGTTTTGATATTTTTGTTTCCACGTGGTGGAAACACTTTTTTGGTTTCAAAGTAACTTTCAAAGAAATAAATCAACAGGTCAATTTT
CAATCAGTCTTTTCTCTATTTCTTTTTTTGGAAGAAGTCTTTATGTTTTCTCTTTCTTTTTCTAATATAAGAATACCCTCTTCGATTCA
TTCAGAGCTGTTAATTAGACGTTTCAAATTTTCGATTCAAGATGTTTCTTTTTTACATTTTTTAAGTTTTATACTTTTTTCAAAGCAAT
TTAAGTTTGTGAATAATTCTATTATTTTTCCAAAAGGAAGTGTGGTTTTCTGTTTCTTATTAGGGAATATTCTTCTTTCTATTTTCGAA
GATTTTTTCACTCTTCGATGGAAAAGTTGTTTTCATGAAAAATCATTGTCTTATGGTCTTTTTTCAGAACAAAAGCATTTTCAACAA
AAATGGAATTTTTTAACCCGAAAACCGAAAAAAAAAGACACGATAATATTGCTAAAGGATTTTTTTTTCACTATATAAGATATGG
GGAAAAAATTGATTTTGCTGGGAACTACTATTCTAGTCAAAAAAATGTGAATTTTTTTTCTTCAATTTTTGGCAAACTTATCTTTTTGTT
TTATCGGAACCCTCTAGTTTTTTTTTGAAACAAATTTCCAGTCAAAATATGTTTTTTCTAGCTTATTACTTAGAATATCCAACAAACT
CTTTTTTACTCCGACTAAATATCTTAGATTATTTTCTATCTACTGATTTTGTTAGCAGGGAATTAAATTCAAAACTTAGCGCTGTTTT
TGTTATTCAATTTTTATCAAAAGAAGGATTATGTGATATAATGGGTAACCCGAAGAGTAAATTAGCATGGCTTAGTTTTACCGACA
ATTCTATTCTTGATAAATATGA

> *Ephedra compacta* 9 (matK). Mexico, borderline Coah-Zac (FCZ). Coah_Zac_9

ATTCTTTCTTTTGAAAGTACTATTTGGTTTATTCGGTTCAAGCTAGATTGTACTATGTGTTATTTTATTTCTAAAAAGAAAGGTTTAG
GAGGTTTTTTCTTGAAAAAAAAATGAAAACATACGTTGGCAACAACATTTTTTATATCCCCTCTTATTCCATAACGATTTCTATGTTA
TAGATCCGAATCTGTTATTCAACTCAAGCCCTTCTTTCGAAAAAATAGAAAAATTACCTAATAGTTTCCGTTTTTTGAATGTAAAAC
GTTCAATTAAGCTATTACATCAACAAAACTATCTTGTGTATAATACAAGAAGTTGTTGTTTTAATTTCATTGGAAAAACTTTTTTTT
CTGTTTTGATATTTTTGTTTCCACGTGGTGGAAACACTTTTTTGGTTTCAAAGTAACTTTCAAAGAAATAAATCAACAGGTCAATTTT
CAATCAGTCTTTTCTCTATTTCTTTTTTTGGAAGAAGTCTTTATGTTTTCTCTTTCTTTTTCTAATATAAGAATACCCTCTTCGATTCA
TTCAGAGCTGTTAATTAGACGTTTCAAATTTTCGATTCAAGATGTTTCTTTTTTACATTTTTTAAGTTTTATACTTTTTTCAAAGCAAT
TTAAGTTTGTGAATAATTCTATTATTTTTCCAAAAGGAAGTGTGGTTTTCTGTTTCTTATTAGGGAATATTCTTCTTTCTATTTTCGAA
GATTTTTTCACTCTTCGATGGAAAAGTTGTTTTCATGAAAAATCATTGTCTTATGGTCTTTTTTCAGAACAAAAGCATTTTCAACAA
AAATGGAATTTTTTAACCCGAAAACCGAAAAAAAAAGACACGATAATATTGCTAAAGGATTTTTTTTTCACTATATAAGATATGG
GGAAAAAATTGATTTTGCTGGGAACTACTATTCTAGTCAAAAAAATGTGAATTTTTTTTCTTCAATTTTTGGCAAACTTATCTTTTTGTT
TTATCGGAACCCTCTAGTTTTTTTTTGAAACAAATTTCCAGTCAAAATATGTTTTTTCTAGCTTATTACTTAGAATATCCAACAAACT
CTTTTTTACTCCGACTAAATATCTTAGATTATTTTCTATCTACTGATTTTGTTAGCAGGGAATTAAATTCAAAACTTAGCGCTGTTTT

TGTTATTCAATTTTTATCAAAAGAAGGATTATGTGATATAATGGGTAACCCGAAGAGTAAATTAGCATGGCTTAGTTTTACCGACA
ATTCTATTCTTGATAAATATGA
> ***Ephedra compacta* 1 (matK). Mexico, Sierra Mojada, Coah (MOJ).** Sierra_Moj_1
ATTCTTTCTTTTGAAAGTACTATTTGGTTTATTCGGTTCAAGCTAGATTGTACTATGTGTTATTTTATTTCTAAAAAGAAAGGTTTAG
GAGGTTTTTTCTTGAAAAAAAAATGAAAACATACGTTGGCAACAACATTTTTTATATCCCCTCTTATTCCATAACGATTTCTATGTTA
TAGATCCGAATCTGTTATTCAACTCAAGCCCTTCTTTCGAAAAAATAGAAAAATTACCTAATAGTTTCCGTTTTTTGAATGTAAAAC
GTTCAATTAAGCTATTACATCAACAAAACTATCTTGTGTATAATACAAGAAGTTCTTGTTTTAATTTCATTGGAAAAACTTTTTTTTT
CTGTTTTGATATTTTTGTTTCCACGTGGTGGAAACACTTTTTTGGTTTCAAAGTAACTTTCAAAGAAATAAATCAACAGGTCAATTTT
CAATCAGTCTTTTCTCTATTTCTTTTTTTGGAAGAAGTCTTTATGTTTTCTCTTTCTTTTTCTAATATAAGAATACCCTCTTCGATTCA
TTCAGAGCTGTTAATTAGACGTTTCAAATTTTCGATTCAAGATGTTTCTTTTTTACATTTTTTAAGTTTTATACTTTTTTCAAAGCAAT
TTAAGTTTGTGAATAATTCTATTATTTTTCCAAAAGGAAGTGTGATTTTCTGTTTCTTATTAGGGAATATTCTTCTTTCTATTTTCGAA
GATTTTTTCACTCTTCGATGGAAAAGTTGTTTTCATGAAAAATCATTGTCTTATGGTCTTTTTTCAGAACAAAAGCATTTTCAACAA
AAATGGAATTTTTTAACCCGAAAACCGAAAAAAAAAGACACGATAAGATTGCTAAAGGATTTTTTTTTCACTATATAAGATATGG
GGAAAAATTGATTTTGCTGGGAACTACTATTCTAGTAAAAAAATGTGAATTTTTTTTCTTAAATTTTGGCAAACTTATCTTTTTGTT
TTATCGGAACCCTCTAGTTTTTTTTTGAAACAAATTTCCAGTCAAAATATATTTTTTCTAGCTTATTACTTAGAATATCCAACAAACT
CTTTTTTACTCCGACTAAATATCTTAGATTATTTTCTATCTACTGATTTTGTTAGCAGGGAATTAAATTCAAAACTTAGCGCTGTTTT
TGTTATTCAATTTTTATCAAAAGAAGGATTATGTGATATAATGGGTAACCCGAAGAGTAAATTAGCATGGCTTAGTTTTACCGACA
ATTCTATTCTTGATAAATATGA
> ***Ephedra compacta* 2 (matK). Mexico, Sierra Mojada, Coah (MOJ).** Sierra_Moj_2
ATTCTTTCTTTTGAAAGTACTATTTGGTTTATTCGGTTCAAGCTAGATTGTACTATGTGTTATTTTATTTCTAAAAAGAAAGGTTTAG
GAGGTTTTTTCTTGAAAAAAAAATGAAAACATACGTTGGCAACAACATTTTTTATATCCCCTCTTATTCCATAACGATTTCTATGTTA
TAGATCCGAATCTGTTATTCAACTCAAGCCCTTCTTTCGAAAAAATAGAAAAATTACCTAATAGTTTCCGTTTTTTGAATGTAAAAC
GTTCAATTAAGCTATTACATCAACAAAACTATCTTGTGTATAATACAAGAAGTTCTTGTTTTAATTTCATTGGAAAAACTTTTTTTTT
CTGTTTTGATATTTTTGTTTCCACGTGGTGGAAACACTTTTTTGGTTTCAAAGTAACTTTCAAAGAAATAAATCAACAGGTCAATTTT
CAATCAGTCTTTTCTCTATTTCTTTTTTTGGAAGAAGTCTTTATGTTTTCTCTTTCTTTTTCTAATATAAGAATACCCTCTTCGATTCA
TTCAGAGCTGTTAATTAGACGTTTCAAATTTTCGATTCAAGATGTTTCTTTTTTACATTTTTTAAGTTTTATACTTTTTTCAAAGCAAT
TTAAGTTTGTGAATAATTCTATTATTTTTCCAAAAGGAAGTGTGATTTTCTGTTTCTTATTAGGGAATATTCTTCTTTCTATTTTCGAA
GATTTTTTCACTCTTCGATGGAAAAGTTGTTTTCATGAAAAATCATTGTCTTATGGTCTTTTTTCAGAACAAAAGCATTTTCAACAA
AAATGGAATTTTTTAACCCGAAAACCGAAAAAAAAAGACACGATAAGATTGCTAAAGGATTTTTTTTTCACTATATAAGATATGG
GGAAAAATTGATTTTGCTGGGAACTACTATTCTAGTAAAAAAATGTGAATTTTTTTTCTTAAATTTTGGCAAACTTATCTTTTTGTT
TTATCGGAACCCTCTAGTTTTTTTTTGAAACAAATTTCCAGTCAAAATATATTTTTTCTAGCTTATTACTTAGAATATCCAACAAACT
CTTTTTTACTCCGACTAAATATCTTAGATTATTTTCTATCTACTGATTTTGTTAGCAGGGAATTAAATTCAAAACTTAGCGCTGTTTT
TGTTATTCAATTTTTATCAAAAGAAGGATTATGTGATATAATGGGTAACCCGAAGAGTAAATTAGCATGGCTTAGTTTTACCGACA
ATTCTATTCTTGATAAATATGA
> ***Ephedra compacta* 3 (matK). Mexico, Sierra Mojada, Coah (MOJ).** Sierra_Moj_3
ATTCTTTCTTTTGAAAGTACTATTTGGTTTATTCGGTTCAAGCTAGATTGTACTATGTGTTATTTTATTTCTAAAAAGAAAGGTTTAG
GAGGTTTTTTCTTGAAAAAAAAATGAAAACATACGTTGGCAACAACATTTTTTATATCCCCTCTTATTCCATAACGATTTCTATGTTA
TAGATCCGAATCTGTTATTCAACTCAAGCCCTTCTTTCGAAAAAATAGAAAAATTACCTAATAGTTTCCGTTTTTTGAATGTAAAAC
GTTCAATTAAGCTATTACATCAACAAAACTATCTTGTGTATAATACAAGAAGTTCTTGTTTTAATTTCATTGGAAAAACTTTTTTTTT
CTGTTTTGATATTTTTGTTTCCACGTGGTGGAAACACTTTTTTGGTTTCAAAGTAACTTTCAAAGAAATAAATCAACAGGTCAATTTT
CAATCAGTCTTTTCTCTATTTCTTTTTTTGGAAGAAGTCTTTATGTTTTCTCTTTCTTTTTCTAATATAAGAATACCCTCTTCGATTCA
TTCAGAGCTGTTAATTAGACGTTTCAAATTTTCGATTCAAGATGTTTCTTTTTTACATTTTTTAAGTTTTATACTTTTTTCAAAGCAAT

TTAAGTTTGTGAATAATTCTATTATTTTTCCAAAAGGAAGTGTGATTTTCTGTTTCTTATTAGGGAATATTCTTCTTTCTATTTTCGAA
GATTTTTTCACTCTTCGATGGAAAAGTTGTTTTCATGAAAAATCATTGTCTTATGGTCTTTTTTCAGAACAAAAGCATTTTCAACAA
AAATGGAATTTTTTAACCCGAAAACCGAAAAAAAAAGACACGATAAGATTGCTAAAGGATTTTTTTTTCACTATATAAGATATGG
GGAAAAAATTGATTTTGCTGGGAACTACTATTCTAGTAAAAAAATGTGAATTTTTTTTCTTAAATTTTTGGCAAACTTATCTTTTTGTT
TTATCGGAACCCTCTAGTTTTTTTTTGAAACAAATTTCCAGTCAAAATATATTTTTTCTAGCTTATTACTTAGAATATCCAACAAACT
CTTTTTTACTCCGACTAAATATCTTAGATTATTTTCTATCTACTGATTTTGTTAGCAGGGAATTAAATTCAAAACTTAGCGCTGTTTT
TGTTATTCAATTTTTATCAAAAGAAGGATTATGTGATATAATGGGTAACCCGAAGAGTAAATTAGCATGGCTTAGTTTTACCGACA
ATTCTATTCTTGATAAATATGA

> *Ephedra compacta* 4 (matK). Mexico, Sierra Mojada, Coah (MOJ). Sierra_Moj_4

ATTCTTTCTTTTGAAAGTACTATTTGGTTTATTCGGTTCAAGCTAGATTGTACTATGTGTTATTTTATTTCTAAAAAGAAAGGTTTAG
GAGGTTTTTTCTTGAAAAAAAAATGAAAACATACGTTGGCAACAACATTTTTTATATCCCCTCTTATTCCATAACGATTTCTATGTTA
TAGATCCGAATCTGTTATTCAACTCAAGCCCTTCTTTCGAAAAAATAGAAAAATTACCTAATAGTTTCCGTTTTTTGAATGTAAAAC
GTTCAATTAAGCTATTACATCAACAAAACTATCTTGTGTATAATACAAGAAGTTCTTGTTTTAATTTCATTGGAAAAACTTTTTTTTT
CTGTTTTGATATTTTTGTTTCCACGTGGTGGAAACACTTTTTTGGTTTCAAAGTAACTTTCAAAGAAATAAATCAACAGGTCAATTTT
CAATCAGTCTTTTCTCTATTTCTTTTTTTGGAAGAAGTCTTTATGTTTTCTCTTTCTTTTTCTAATATAAGAATACCCTCTTCGATTCA
TTCAGAGCTGTTAATTAGACGTTTCAAATTTTCGATTCAAGATGTTTCTTTTTTACATTTTTTAAGTTTTATACTTTTTTCAAAGCAAT
TTAAGTTTGTGAATAATTCTATTATTTTTCCAAAAGGAAGTGTGATTTTCTGTTTCTTATTAGGGAATATTCTTCTTTCTATTTTCGAA
GATTTTTTCACTCTTCGATGGAAAAGTTGTTTTCATGAAAAATCATTGTCTTATGGTCTTTTTTCAGAACAAAAGCATTTTCAACAA
AAATGGAATTTTTTAACCCGAAAACCGAAAAAAAAAGACACGATAAGATTGCTAAAGGATTTTTTTTTCACTATATAAGATATGG
GGAAAAAATTGATTTTGCTGGGAACTACTATTCTAGTAAAAAAATGTGAATTTTTTTTCTTAAATTTTTGGCAAACTTATCTTTTTGTT
TTATCGGAACCCTCTAGTTTTTTTTTGAAACAAATTTCCAGTCAAAATATATTTTTTCTAGCTTATTACTTAGAATATCCAACAAACT
CTTTTTTACTCCGACTAAATATCTTAGATTATTTTCTATCTACTGATTTTGTTAGCAGGGAATTAAATTCAAAACTTAGCGCTGTTTT
TGTTATTCAATTTTTATCAAAAGAAGGATTATGTGATATAATGGGTAACCCGAAGAGTAAATTAGCATGGCTTAGTTTTACCGACA
ATTCTATTCTTGATAAATATGA

> *Ephedra compacta* 5 (matK). Mexico, Sierra Mojada, Coah (MOJ). Sierra_Moj_5

ATTCTTTCTTTTGAAAGTACTATTTGGTTTATTCGGTTCAAGCTAGATTGTACTATGTGTTATTTTATTTCTAAAAAGAAAGGTTTAG
GAGGTTTTTTCTTGAAAAAAAAATGAAAACATACGTTGGCAACAACATTTTTTATATCCCCTCTTATTCCATAACGATTTCTATGTTA
TAGATCCGAATCTGTTATTCAACTCAAGCCCTTCTTTCGAAAAAATAGAAAAATTACCTAATAGTTTCCGTTTTTTGAATGTAAAAC
GTTCAATTAAGCTATTACATCAACAAAACTATCTTGTGTATAATACAAGAAGTTCTTGTTTTAATTTCATTGGAAAAACTTTTTTTTT
CTGTTTTGATATTTTTGTTTCCACGTGGTGGAAACACTTTTTTGGTTTCAAAGTAACTTTCAAAGAAATAAATCAACAGGTCAATTTT
CAATCAGTCTTTTCTCTATTTCTTTTTTTGGAAGAAGTCTTTATGTTTTCTCTTTCTTTTTCTAATATAAGAATACCCTCTTCGATTCA
TTCAGAGCTGTTAATTAGACGTTTCAAATTTTCGATTCAAGATGTTTCTTTTTTACATTTTTTAAGTTTTATACTTTTTTCAAAGCAAT
TTAAGTTTGTGAATAATTCTATTATTTTTCCAAAAGGAAGTGTGATTTTCTGTTTCTTATTAGGGAATATTCTTCTTTCTATTTTCGAA
GATTTTTTCACTCTTCGATGGAAAAGTTGTTTTCATGAAAAATCATTGTCTTATGGTCTTTTTTCAGAACAAAAGCATTTTCAACAA
AAATGGAATTTTTTAACCCGAAAACCGAAAAAAAAAGACACGATAAGATTGCTAAAGGATTTTTTTTTCACTATATAAGATATGG
GGAAAAAATTGATTTTGCTGGGAACTACTATTCTAGTAAAAAAATGTGAATTTTTTTTCTTAAATTTTTGGCAAACTTATCTTTTTGTT
TTATCGGAACCCTCTAGTTTTTTTTTGAAACAAATTTCCAGTCAAAATATATTTTTTCTAGCTTATTACTTAGAATATCCAACAAACT
CTTTTTTACTCCGACTAAATATCTTAGATTATTTTCTATCTACTGATTTTGTTAGCAGGGAATTAAATTCAAAACTTAGCGCTGTTTT
TGTTATTCAATTTTTATCAAAAGAAGGATTATGTGATATAATGGGTAACCCGAAGAGTAAATTAGCATGGCTTAGTTTTACCGACA
ATTCTATTCTTGATAAATATGA

> *Ephedra compacta* 1 (matK). Mexico, Real de Catorce SLP (RC). Real_C_1

ATTCTTTCTTTTGAAAGTACTATTTGGTTTATTCGGTTCAAGCTAGATTGTACTATGTGTTATTTTATTTCTAAAAAGAAAGGTTTAG
GAGGTTTTTTCTTGAAAAAAAATGAAAACATACGTTGGCAACAACATTTTTTATATCCCCTCTTATTCCATAACGATTTCTATGTTA
TAGATCCGAATCTGTTATTCAACTCAAGCCCTTCTTTCGAAAAAATAGAAAAATTACCTAATAGTTTCCGTTTTTTGAATGTAAAAC
GTTCAATTAAGCTATTACATCAACAAAACTATCTTGTGTATAATACAAGAAGTTCTTGTTTTAATTTCATTGGAAAAACTTTTTTTTT
CTGTTTTGATATTTTTGTTTCCACGTGGTGGAAACACTTTTTTGGTTTCAAAGTAACTTTCAAAGAAATAAATCAACAGGTCAATTTT
CAATCAGTCTTTTCTCTATTTCTTTTTTTGGAAGAAGTCTTTATGTTTTCTCTTTCTTTTTCTAATATAAGAATACCCTCTTCGATTCA
TTCAGAGCTGTTAATTAGACGTTTCAAATTTTCGATTCAAGATGTTTCTTTTTTACATTTTTTAAGTTTTATACTTTTTTCAAAGCAAT
TTAAGTTTGTGAATAATTCTATTATTTTTCCAAAAGGAAGTGTGATTTTCTGTTTCTTATTAGGGAATATTCTTCTTTCTATTTTCGAA
GATTTTTTCACTCTTCGATGGAAAAGTTGTTTTCATGAAAAATCATTGTCTTATGGTCTTTTTTCAGAACAAAAGCATTTTCAACAA
AAATGGAATTTTTTAACCCGAAAACCGAAAAAAAAAGACACGATAATATTGCTAAAGGATTTTTTTTTTCACTATATAAGATATGG
GGAAAAAATTGATTTTGCTGGGAACTACTATTCTAGTCAAAAAATGTGAATTTTTTTTCTTAAATTTTGGCAAACTTATCTTTTTGTT
TTATCGGAACCCTCTAGTTTTTTTTTGAAACAAATTTCCAGTCAAAATATGTTTTTTCTAGCTTATTACTTAGAATATCCAACAAACT
CTTTTTTACTCCGACTAAATATCTTAGATTATTTTCTATCTACTGATTTTGTTAGCAGGGAATTAAATTCAAAACTTAGCGCTGTTTT
TGTTATTCAATTTTTATCAAAAGAAGGATTATGTGATATAATGGGTAACCCGAAGAGTAAATTAGCATGGCTTAGTTTTACCGACA
ATTCTATTCTTGATAAATATGA

> ***Ephedra compacta*** **2 (matK). Mexico, Real de Catorce SLP (RC).** Real_C_2

ATTCTTTCTTTTGAAAGTACTATTTGGTTTATTCGGTTCAAGCTAGATTGTACTATGTGTTATTTTATTTCTAAAAAGAAAGGTTTAG
GAGGTTTTTTCTTGAAAAAAAATGAAAACATACGTTGGCAACAACATTTTTTATATCCCCTCTTATTCCATAACGATTTCTATGTTA
TAGATCCGAATCTGTTATTCAACTCAAGCCCTTCTTTCGAAAAAATAGAAAAATTACCTAATAGTTTCCGTTTTTTGAATGTAAAAC
GTTCAATTAAGCTATTACATCAACAAAACTATCTTGTGTATAATACAAGAAGTTCTTGTTTTAATTTCATTGGAAAAACTTTTTTTTT
CTGTTTTGATATTTTTGTTTCCACGTGGTGGAAACACTTTTTTGGTTTCAAAGTAACTTTCAAAGAAATAAATCAACAGGTCAATTTT
CAATCAGTCTTTTCTCTATTTCTTTTTTTGGAAGAAGTCTTTATGTTTTCTCTTTCTTTTTCTAATATAAGAATACCCTCTTCGATTCA
TTCAGAGCTGTTAATTAGACGTTTCAAATTTTCGATTCAAGATGTTTCTTTTTTACATTTTTTAAGTTTTATACTTTTTTCAAAGCAAT
TTAAGTTTGTGAATAATTCTATTATTTTTCCAAAAGGAAGTGTGATTTTCTGTTTCTTATTAGGGAATATTCTTCTTTCTATTTTCGAA
GATTTTTTCACTCTTCGATGGAAAAGTTGTTTTCATGAAAAATCATTGTCTTATGGTCTTTTTTCAGAACAAAAGCATTTTCAACAA
AAATGGAATTTTTTAACCCGAAAACCGAAAAAAAAAGACACGATAATATTGCTAAAGGATTTTTTTTTTCACTATATAAGATATGG
GGAAAAAATTGATTTTGCTGGGAACTACTATTCTAGTCAAAAAATGTGAATTTTTTTTCTTAAATTTTGGCAAACTTATCTTTTTGTT
TTATCGGAACCCTCTAGTTTTTTTTTGAAACAAATTTCCAGTCAAAATATGTTTTTTCTAGCTTATTACTTAGAATATCCAACAAACT
CTTTTTTACTCCGACTAAATATCTTAGATTATTTTCTATCTACTGATTTTGTTAGCAGGGAATTAAATTCAAAACTTAGCGCTGTTTT
TGTTATTCAATTTTTATCAAAAGAAGGATTATGTGATATAATGGGTAACCCGAAGAGTAAATTAGCATGGCTTAGTTTTACCGACA
ATTCTATTCTTGATAAATATGA

> ***Ephedra compacta*** **1 (matK). Mexico, Rocamontes, Dgo (ROCA).** Rocamontes_1

ATTCTTTCTTTTGAAAGTACTATTTGGTTTATTCGGTTCAAGCTAGATTGTACTATGTGTTATTTTATTTCTAAAAAGAAAGGTTTAG
GAGGTTTTTTCTTGAAAAAAAATGAAAACATACGTTGGCAACAACATTTTTTATATCCCCTCTTATTCCATAACGATTTCTATGTTA
TAGATCCGAATCTGTTATTCAACTCAAGCCCTTCTTTCGAAAAAATAGAAAAATTACCTAATAGTTTCCGTTTTTTGAATGTAAAAC
GTTCAATTAAGCTATTACATCAACAAAACTATCTTGTGTATAATACAAGAAGTTGTTGTTTTAATTTCATTGGAAAAACTTTTTTTTT
CTGTTTTGATATTTTTGTTTCCACGTGGTGGAAACACTTTTTTGGTTTCAAAGTAACTTTCAAAGAAATAAATCAACAGGTCAATTTT
CAATCAGTCTTTTCTCTATTTCTTTTTTTGGAAGAAGTCTTTATGTTTTCTCTTTCTTTTTCTAATATAAGAATACCCTCTTCGATTCA
TTCAGAGCTGTTAATTAGACGTTTCAAATTTTCGATTCAAGATGTTTCTTTTTTACATTTTTTAAGTTTTATACTTTTTTCAAAGCAAT
TTAAGTTTGTGAATAATTCTATTATTTTTCCAAAAGGAAGTGTGGTTTTCTGTTTCTTATTAGGGAATATTCTTCTTTCTATTTTCGAA
GATTTTTTCACTCTTCGATGGAAAAGTTGTTTTCATGAAAAATCATTGTCTTATGGTCTTTTTTCAGAACAAAAGCATTTTCAACAA
AAATGGAATTTTTTAACCCGAAAACCGAAAAAAAAAGACACGATAATATTGCTAAAGGATTTTTTTTTTCACTATATAAGATATGG

GGAAAAAATTGATTTTGCTGGGAACTACTATTCTAGTCAAAAAATGTGAATTTTTTTTCTTCAATTTTTGGCAAACTTATCTTTTTGTT
TTATCGGAACCCTCTAGTTTTTTTTTGAAACAAATTTCCAGTCAAAATATGTTTTTTCTAGCTTATTACTTAGAATATCCAACAAACT
CTTTTTTACTCCGACTAAATATCTTAGATTATTTTCTATCTACTGATTTTGTTAGCAGGGAATTAAATTCAAAACTTAGCGCTGTTTT
TGTTATTCAATTTTTATCAAAAGAAGGATTATGTGATATAATGGGTAACCCGAAGAGTAAATTAGCATGGCTTAGTTTTACCGACA
ATTCTATTCTTGATAAATATGA

> *Ephedra compacta* 4 (matK). Mexico, Rocamontes, Dgo (ROCA). Rocamontes_4
ATTCTTTCTTTTGAAAGTACTATTTGGTTTATTCGGTTCAAGCTAGATTGTACTATGTGTTATTTTATTTCTAAAAAGAAAGGTTTAG
GAGGTTTTTTCTTGAAAAAAAAATGAAAACATACGTTGGCAACAACATTTTTTATATCCCCTCTTATTCCATAACGATTTCTATGTTA
TAGATCCGAATCTGTTATTCAACTCAAGCCCTTCTTTCGAAAAAATAGAAAAATTACCTAATAGTTTCCGTTTTTTGAATGTAAAAC
GTTCAATTAAGCTATTACATCAACAAAACTATCTTGTGTATAATACAAGAAGTTGTTGTTTTAATTTCATTGGAAAAACTTTTTTTTT
CTGTTTTGATATTTTTGTTTCCACGTGGTGGAAACACTTTTTTGGTTTCAAAGTAACTTTCAAAGAAATAAATCAACAGGTCAATTTT
CAATCAGTCTTTTCTCTATTTCTTTTTTTGGAAGAAGTCTTTATGTTTTCTCTTTCTTTTTCTAATATAAGAATACCCTCTTCGATTCA
TTCAGAGCTGTTAATTAGACGTTTCAAATTTTCGATTCAAGATGTTTCTTTTTTACATTTTTTAAGTTTTATACTTTTTTCAAAGCAAT
TTAAGTTTGTGAATAATTCTATTATTTTTCCAAAAGGAAGTGTGGTTTTCTGTTTCTTATTAGGGAATATTCTTCTTTCTATTTTCGAA
GATTTTTTCACTCTTCGATGGAAAAGTTGTTTTCATGAAAAATCATTGTCTTATGGTCTTTTTTCAGAACAAAAGCATTTTCAACAA
AAATGGAATTTTTTAACCCGAAAACCGAAAAAAAAAGACACGATAATATTGCTAAAGGATTTTTTTTTTCACTATATAAGATATGG
GGAAAAAATTGATTTTGCTGGGAACTACTATTCTAGTCAAAAAATGTGAATTTTTTTTCTTCAATTTTTGGCAAACTTATCTTTTTGTT
TTATCGGAACCCTCTAGTTTTTTTTTGAAACAAATTTCCAGTCAAAATATGTTTTTTCTAGCTTATTACTTAGAATATCCAACAAACT
CTTTTTTACTCCGACTAAATATCTTAGATTATTTTCTATCTACTGATTTTGTTAGCAGGGAATTAAATTCAAAACTTAGCGCTGTTTT
TGTTATTCAATTTTTATCAAAAGAAGGATTATGTGATATAATGGGTAACCCGAAGAGTAAATTAGCATGGCTTAGTTTTACCGACA
ATTCTATTCTTGATAAATATGA

> *Ephedra compacta* 5 (matK). Mexico, Rocamontes, Dgo (ROCA). Rocamontes_5
ATTCTTTCTTTTGAAAGTACTATTTGGTTTATTCGGTTCAAGCTAGATTGTACTATGTGTTATTTTATTTCTAAAAAGAAAGGTTTAG
GAGGTTTTTTCTTGAAAAAAAAATGAAAACATACGTTGGCAACAACATTTTTTATATCCCCTCTTATTCCATAACGATTTCTATGTTA
TAGATCCGAATCTGTTATTCAACTCAAGCCCTTCTTTCGAAAAAATAGAAAAATTACCTAATAGTTTCCGTTTTTTGAATGTAAAAC
GTTCAATTAAGCTATTACATCAACAAAACTATCTTGTGTATAATACAAGAAGTTGTTGTTTTAATTTCATTGGAAAAACTTTTTTTTT
CTGTTTTGATATTTTTGTTTCCACGTGGTGGAAACACTTTTTTGGTTTCAAAGTAACTTTCAAAGAAATAAATCAACAGGTCAATTTT
CAATCAGTCTTTTCTCTATTTCTTTTTTTGGAAGAAGTCTTTATGTTTTCTCTTTCTTTTTCTAATATAAGAATACCCTCTTCGATTCA
TTCAGAGCTGTTAATTAGACGTTTCAAATTTTCGATTCAAGATGTTTCTTTTTTACATTTTTTAAGTTTTATACTTTTTTCAAAGCAAT
TTAAGTTTGTGAATAATTCTATTATTTTTCCAAAAGGAAGTGTGGTTTTCTGTTTCTTATTAGGGAATATTCTTCTTTCTATTTTCGAA
GATTTTTTCACTCTTCGATGGAAAAGTTGTTTTCATGAAAAATCATTGTCTTATGGTCTTTTTTCAGAACAAAAGCATTTTCAACAA
AAATGGAATTTTTTAACCCGAAAACCGAAAAAAAAAGACACGATAATATTGCTAAAGGATTTTTTTTTTCACTATATAAGATATGG
GGAAAAAATTGATTTTGCTGGGAACTACTATTCTAGTCAAAAAATGTGAATTTTTTTTCTTCAATTTTTGGCAAACTTATCTTTTTGTT
TTATCGGAACCCTCTAGTTTTTTTTTGAAACAAATTTCCAGTCAAAATATGTTTTTTCTAGCTTATTACTTAGAATATCCAACAAACT
CTTTTTTACTCCGACTAAATATCTTAGATTATTTTCTATCTACTGATTTTGTTAGCAGGGAATTAAATTCAAAACTTAGCGCTGTTTT
TGTTATTCAATTTTTATCAAAAGAAGGATTATGTGATATAATGGGTAACCCGAAGAGTAAATTAGCATGGCTTAGTTTTACCGACA
ATTCTATTCTTGATAAATATGA

> *Ephedra compacta* 6 (matK). Mexico, Rocamontes, Dgo (ROCA). Rocamontes_6
ATTCTTTCTTTTGAAAGTACTATTTGGTTTATTCGGTTCAAGCTAGATTGTACTATGTGTTATTTTATTTCTAAAAAGAAAGGTTTAG
GAGGTTTTTTCTTGAAAAAAAAATGAAAACATACGTTGGCAACAACATTTTTTATATCCCCTCTTATTCCATAACGATTTCTATGTTA
TAGATCCGAATCTGTTATTCAACTCAAGCCCTTCTTTCGAAAAAATAGAAAAATTACCTAATAGTTTCCGTTTTTTGAATGTAAAAC
GTTCAATTAAGCTATTACATCAACAAAACTATCTTGTGTATAATACAAGAAGTTGTTGTTTTAATTTCATTGGAAAAACTTTTTTTTT

CTGTTTTGATATTTTTGTTTCCACGTGGTGGAAACACTTTTTTGGTTTCAAAGTAACTTTCAAAGAAATAAATCAACAGGTCAATTTT
CAATCAGTCTTTTCTCTATTTCTTTTTTTGGAAGAAGTCTTTATGTTTTCTCTTTCTTTTTCTAATATAAGAATACCCTCTTCGATTCA
TTCAGAGCTGTTAATTAGACGTTTCAAATTTTCGATTCAAGATGTTTCTTTTTTACATTTTTTAAGTTTTATACTTTTTTCAAAGCAAT
TTAAGTTTGTGAATAATTCTATTATTTTTCCAAAAGGAAGTGTGGTTTTCTGTTTCTTATTAGGGAATATTCTTCTTTCTATTTTCGAA
GATTTTTTCACTCTTCGATGGAAAAGTTGTTTTCATGAAAAATCATTGTCTTATGGTCTTTTTTCAGAACAAAAGCATTTTCAACAA
AAATGGAATTTTTTAACCCGAAAACCGAAAAAAAAAGACACGATAATATTGCTAAAGGATTTTTTTTTCACTATATAAGATATGG
GGAAAAATTGATTTTGCTGGGAACTACTATTCTAGTCAAAAAATGTGAATTTTTTTTCTTCAATTTTTGGCAAACTTATCTTTTTGTT
TTATCGGAACCCTCTAGTTTTTTTTTGAAACAAATTTCCAGTCAAAATATGTTTTTTCTAGCTTATTACTTAGAATATCCAACAAACT
CTTTTTTACTCCGACTAAATATCTTAGATTATTTTCTATCTACTGATTTTGTTAGCAGGGAATTAAATTCAAAACTTAGCGCTGTTTT
TGTTATTCAATTTTTATCAAAAGAAGGATTATGTGATATAATGGGTAACCCGAAGAGTAAATTAGCATGGCTTAGTTTTACCGACA
ATTCTATTCTTGATAAATATGA

> *Ephedra compacta* 7. (matK). Mexico, Rocamontes, Dgo (ROCA). Rocamontes_7

ATTCTTTCTTTTGAAAGTACTATTTGGTTTATTCGGTTCAAGCTAGATTGTACTATGTGTTATTTTATTTCTAAAAAGAAAGGTTTAG
GAGGTTTTTTCTTGAAAAAAAAATGAAAACATACGTTGGCAACAACATTTTTTATATCCCCTCTTATTCCATAACGATTTCTATGTTA
TAGATCCGAATCTGTTATTCAACTCAAGCCCTTCTTTCGAAAAAATAGAAAAATTACCTAATAGTTTCCGTTTTTTGAATGTAAAAC
GTTCAATTAAGCTATTACATCAACAAAACTATCTTGTGTATAATACAAGAAGTTGTTGTTTTAATTTCATTGGAAAAACTTTTTTTTT
CTGTTTTGATATTTTTGTTTCCACGTGGTGGAAACACTTTTTTGGTTTCAAAGTAACTTTCAAAGAAATAAATCAACAGGTCAATTTT
CAATCAGTCTTTTCTCTATTTCTTTTTTTGGAAGAAGTCTTTATGTTTTCTCTTTCTTTTTCTAATATAAGAATACCCTCTTCGATTCA
TTCAGAGCTGTTAATTAGACGTTTCAAATTTTCGATTCAAGATGTTTCTTTTTTACATTTTTTAAGTTTTATACTTTTTTCAAAGCAAT
TTAAGTTTGTGAATAATTCTATTATTTTTCCAAAAGGAAGTGTGGTTTTCTGTTTCTTATTAGGGAATATTCTTCTTTCTATTTTCGAA
GATTTTTTCACTCTTCGATGGAAAAGTTGTTTTCATGAAAAATCATTGTCTTATGGTCTTTTTTCAGAACAAAAGCATTTTCAACAA
AAATGGAATTTTTTAACCCGAAAACCGAAAAAAAAAGACACGATAATATTGCTAAAGGATTTTTTTTTCACTATATAAGATATGG
GGAAAAATTGATTTTGCTGGGAACTACTATTCTAGTCAAAAAATGTGAATTTTTTTTCTTCAATTTTTGGCAAACTTATCTTTTTGTT
TTATCGGAACCCTCTAGTTTTTTTTTGAAACAAATTTCCAGTCAAAATATGTTTTTTCTAGCTTATTACTTAGAATATCCAACAAACT
CTTTTTTACTCCGACTAAATATCTTAGATTATTTTCTATCTACTGATTTTGTTAGCAGGGAATTAAATTCAAAACTTAGCGCTGTTTT
TGTTATTCAATTTTTATCAAAAGAAGGATTATGTGATATAATGGGTAACCCGAAGAGTAAATTAGCATGGCTTAGTTTTACCGACA
ATTCTATTCTTGATAAATATGA

> *Ephedra compacta* 1. (matK). Mexico, Cuatro Cienegas, Coah (CC). Cuatro_Cie_1

ATTCTTTCTTTTGAAAGTACTATTTGGTTTATTCGGTTCAAGCTAGATTGTACTATGTGTTATTTTATTTCTAAAAAGAAAGGTTTAG
GAGGTTTTTTCTTGAAAAAAAAATGAAAACATACGTTGGCAACAACATTTTTTATATCCCCTCTTATTCCATAACGATTTCTATGTTA
TAGATCCGAATCTGTTATTCAACTCAAGCCCTTCTTTCGAAAAAATAGAAAAATTACCTAATAGTTTCCGTTTTTTGAATGTAAAAC
GTTCAATTAAGCTATTACATCAACAAAACTATCTTGTGTATAATACAAGAAGTTCTTGTTTTAATTTCATTGGAAAAACTTTTTTTTT
CTGTTTTGATATTTTTGTTTCCACGTGGTGGAAACACTTTTTTGGTTTCAAAGTAACTTTCAAAGAAATAAATCAACAGGTCAATTTT
CAATCAGTCTTTTCTCTATTTCTTTTTTTGGAAGAAGTCTTTATGTTTTCTCTTTCTTTTTCTAATATAAGAATACCCTCTTCGATTCA
TTCAGAGCTGTTAATTAGACGTTTCAAATTTTCGATTCAAGATGTTTCTTTTTTACATTTTTTAAGTTTTATACTTTTTTCAAAGCAAT
TTAAGTTTGTGAATAATTCTATTATTTTTCCAAAAGGAAGTGTGATTTTCTGTTTCTTATTAGGGAATATTCTTCTTTCTATTTTCGAA
GATTTTTTCACTCTTCGATGGAAAAGTTGTTTTCATGAAAAATCATTGTCTTATGGTCTTTTTTCAGAACAAAAGCATTTTCAACAA
AAATGGAATTTTTTAACCCGAAAACCGAAAAAAAAAGACACGATAAGATTGCTAAAGGATTTTTTTTTCACTATATAAGATATGG
GGAAAAATTGATTTTGCTGGGAACTACTATTCTAGTCAAAAAATGTGAATTTTTTTTCTTAAATTTTTGGCAAACTTATCTTTTTGTT
TTATCGGAACCCTCTAGTTTTTTTTTGAAACAAATTTCCAGTCAAAATATATTTTTTCTAGCTTATTACTTAGAATATCCAACAAACT
CTTTTTTACTCCGACTAAATATCTTAGATTATTTTCTATCTACTGATTTTGTTAGCAGGGAATTAAATTCAAAACTTAGCGCTGTTTT

TGTTATTCAATTTTTATCAAAAGAAGGATTATGTGATATAATGGGTAACCCGAAGAGTAAATTAGCATGGCTTAGTTTTACCGACA
ATTCTATTCTTGATAAATATGA

> *Ephedra compacta* 2. (matK). Mexico, Cuatro Cienegas, Coah (CC). Cuatro_Cie_2

ATTCTTTCTTTTGAAAGTACTATTTGGTTTATTCGGTTCAAGCTAGATTGTACTATGTGTTATTTTATTTCTAAAAAGAAAGGTTTAG
GAGGTTTTTTCTTGAAAAAAAAATGAAAACATACGTTGGCAACAACATTTTTTATATCCCCTCTTATTCCATAACGATTTCTATGTTA
TAGATCCGAATCTGTTATTCAACTCAAGCCCTTCTTTCGAAAAAATAGAAAAATTACCTAATAGTTTCCGTTTTTTGAATGTAAAAC
GTTCAATTAAGCTATTACATCAACAAAACTATCTTGTGTATAATACAAGAAGTTCTTGTTTTAATTTCATTGGAAAAACTTTTTTTTT
CTGTTTTGATATTTTTGTTTCCACGTGGTGGAAACACTTTTTTGGTTTCAAAGTAACTTTCAAAGAAATAAATCAACAGGTCAATTTT
CAATCAGTCTTTTCTCTATTTCTTTTTTTGGAAGAAGTCTTTATGTTTTCTCTTTCTTTTTCTAATATAAGAATACCCTCTTCGATTCA
TTCAGAGCTGTTAATTAGACGTTTCAAATTTTCGATTCAAGATGTTTCTTTTTTACATTTTTTAAGTTTATACTTTTTTCAAAGCAAT
TTAAGTTTGTGAATAATTCTATTATTTTTCCAAAAGGAAGTGTGATTTTCTGTTTCTTATTAGGGAATATTCTTCTTTCTATTTTCGAA
GATTTTTTCACTCTTCGATGGAAAAGTTGTTTTCATGAAAAAATCATTGTCTTATGGTCTTTTTTCAGAACAAAAGCATTTTCAACAA
AAATGGAATTTTTTAACCCGAAAACCGAAAAAAAAAAGACACGATAAGATTGCTAAAGGATTTTTTTTTCACTATATAAGATATGG
GGAAAAATTGATTTGCTGGGAACTACTATTCTAGTCAAAAAATGTGAATTTTTTTTCTTAAATTTTGGCAAACTTATCTTTTTGTT
TTATCGGAACCCTCTAGTTTTTTTTTGAAACAAATTTCCAGTCAAAATATATTTTTTCTAGCTTATTACTTAGAATATCCAACAAACT
CTTTTTTACTCCGACTAAATATCTTAGATTATTTTCTATCTACTGATTTTGTTAGCAGGGAATTAAATTCAAAACTTAGCGCTGTTTT
TGTTATTCAATTTTTATCAAAAGAAGGATTATGTGATATAATGGGTAACCCGAAGAGTAAATTAGCATGGCTTAGTTTTACCGACA
ATTCTATTCTTGATAAATATGA

> *Ephedra compacta* 4. (matK). Mexico, Cuatro Cienegas, Coah (CC). Cuatro_Cie_4

ATTCTTTCTTTTGAAAGTACTATTTGGTTTATTCGGTTCAAGCTAGATTGTACTATGTGTTATTTTATTTCTAAAAAGAAAGGTTTAG
GAGGTTTTTTCTTGAAAAAAAAATGAAAACATACGTTGGCAACAACATTTTTTATATCCCCTCTTATTCCATAACGATTTCTATGTTA
TAGATCCGAATCTGTTATTCAACTCAAGCCCTTCTTTCGAAAAAATAGAAAAATTACCTAATAGTTTCCGTTTTTTGAATGTAAAAC
GTTCAATTAAGCTATTACATCAACAAAACTATCTTGTGTATAATACAAGAAGTTCTTGTTTTAATTTCATTGGAAAAACTTTTTTTTT
CTGTTTTGATATTTTTGTTTCCACGTGGTGGAAACACTTTTTTGGTTTCAAAGTAACTTTCAAAGAAATAAATCAACAGGTCAATTTT
CAATCAGTCTTTTCTCTATTTCTTTTTTTGGAAGAAGTCTTTATGTTTTCTCTTTCTTTTTCTAATATAAGAATACCCTCTTCGATTCA
TTCAGAGCTGTTAATTAGACGTTTCAAATTTTCGATTCAAGATGTTTCTTTTTTACATTTTTTAAGTTTATACTTTTTTCAAAGCAAT
TTAAGTTTGTGAATAATTCTATTATTTTTCCAAAAGGAAGTGTGATTTTCTGTTTCTTATTAGGGAATATTCTTCTTTCTATTTTCGAA
GATTTTTTCACTCTTCGATGGAAAAGTTGTTTTCATGAAAAAATCATTGTCTTATGGTCTTTTTTCAGAACAAAAGCATTTTCAACAA
AAATGGAATTTTTTAACCCGAAAACCGAAAAAAAAAAGACACGATAAGATTGCTAAAGGATTTTTTTTTCACTATATAAGATATGG
GGAAAAATTGATTTGCTGGGAACTACTATTCTAGTCAAAAAATGTGAATTTTTTTTCTTAAATTTTGGCAAACTTATCTTTTTGTT
TTATCGGAACCCTCTAGTTTTTTTTTGAAACAAATTTCCAGTCAAAATATATTTTTTCTAGCTTATTACTTAGAATATCCAACAAACT
CTTTTTTACTCCGACTAAATATCTTAGATTATTTTCTATCTACTGATTTTGTTAGCAGGGAATTAAATTCAAAACTTAGCGCTGTTTT
TGTTATTCAATTTTTATCAAAAGAAGGATTATGTGATATAATGGGTAACCCGAAGAGTAAATTAGCATGGCTTAGTTTTACCGACA
ATTCTATTCTTGATAAATATGA

> *Ephedra compacta* 1. (matK). Mexico, Parras, Coah (PARR). Parras_1

ATTCTTTCTTTTGAAAGTACTATTTGGTTTATTCGGTTCAAGCTAGATTGTACTATGTGTTATTTTATTTCTAAAAAGAAAGGTTTAG
GAGGTTTTTTCTTGAAAAAAAAATGAAAACATACGTTGGCAACAACATTTTTTATATCCCCTCTTATTCCATAACGATTTCTATGTTA
TAGATCCGAATCTGTTATTCAACTCAAGCCCTTCTTTCGAAAAAATAGAAAAATTACCTAATAGTTTCCGTTTTTTGAATGTAAAAC
GTTCAATTAAGCTATTACATCAACAAAACTATCTTGTGTATAATACAAGAAGTTCTTGTTTTAATTTCATTGGAAAAACTTTTTTTTT
CTGTTTTGATATTTTTGTTTCCACGTGGTGGAAACACTTTTTTGGTTTCAAAGTAACTTTCAAAGAAATAAATCAACAGGTCAATTTT
CAATCAGTCTTTTCTCTATTTCTTTTTTTGGAAGAAGTCTTTATGTTTTCTCTTTCTTTTTCTAATATAAGAATACCCTCTTCGATTCA
TTCAGAGCTGTTAATTAGACGTTTCAAATTTTCGATTCAAGATGTTTCTTTTTTACATTTTTTAAGTTTATACTTTTTTCAAAGCAAT

TTAAGTTTGTGAATAATTCTATTATTTTTCCAAAAGGAAGTGTGATTTTCTGTTTCTTATTAGGGAATATTCTTCTTTCTATTTTCGAA
GATTTTTTCACTCTTCGATGGAAAAGTTGTTTTCATGAAAAATCATTGTCTTATGGTCTTTTTTCAGAACAAAAGCATTTTCAACAA
AAATGGAATTTTTTAACCCGAAAACCGAAAAAAAAAGACACGATAAGATTGCTAAAGGATTTTTTTTTCACTATATAAGATATGG
GGAAAAAATTGATTTTGCTGGGAACTACTATTCTAGTCAAAAAATGTGAATTTTTTTTCTTAAATTTTTGGCAAACTTATCTTTTTGTT
TTATCGGAACCCTCTAGTTTTTTTTTGAAACAAATTTCCAGTCAAAATATATTTTTTCTAGCTTATTACTTAGAATATCCAACAAACT
CTTTTTTACTCCGACTAAATATCTTAGATTATTTTCTATCTACTGATTTTGTTAGCAGGGAATTAAATTCAAAACTTAGCGCTGTTTT
TGTTATTCAATTTTTATCAAAAGAAGGATTATGTGATATAATGGGTAACCCGAAGAGTAAATTAGCATGGCTTAGTTTTACCGACA
ATTCTATTCTTGATAAATATGA

> *Ephedra compacta* 3. (matK). Mexico, Parras, Coah (PARR). Parras_3

ATTCTTTCTTTTGAAAGTACTATTTGGTTTATTCGGTTCAAGCTAGATTGTACTATGTGTTATTTTATTTCTAAAAAGAAAGGTTTAG
GAGGTTTTTTCTTGAAAAAAAAATGAAAACATACGTTGGCAACAACATTTTTTATATCCCCTCTTATTCCATAACGATTTCTATGTTA
TAGATCCGAATCTGTTATTCAACTCAAGCCCTTCTTTCGAAAAAATAGAAAAATTACCTAATAGTTTCCGTTTTTTGAATGTAAAAC
GTTCAATTAAGCTATTACATCAACAAAACTATCTTGTGTATAATACAAGAAGTTCTTGTTTTAATTTCATTGGAAAAACTTTTTTTTT
CTGTTTTGATATTTTTGTTTCCACGTGGTGGAAACACTTTTTTGGTTTCAAAGTAACTTTCAAAGAAATAAATCAACAGGTCAATTTT
CAATCAGTCTTTTCTCTATTTCTTTTTTTGGAAGAAGTCTTTATGTTTTCTCTTTCTTTTTCTAATATAAGAATACCCTCTTCGATTCA
TTCAGAGCTGTTAATTAGACGTTTCAAATTTTCGATTCAAGATGTTTCTTTTTTACATTTTTTAAGTTTTATACTTTTTTCAAAGCAAT
TTAAGTTTGTGAATAATTCTATTATTTTTCCAAAAGGAAGTGTGATTTTCTGTTTCTTATTAGGGAATATTCTTCTTTCTATTTTCGAA
GATTTTTTCACTCTTCGATGGAAAAGTTGTTTTCATGAAAAATCATTGTCTTATGGTCTTTTTTCAGAACAAAAGCATTTTCAACAA
AAATGGAATTTTTTAACCCGAAAACCGAAAAAAAAAGACACGATAAGATTGCTAAAGGATTTTTTTTTCACTATATAAGATATGG
GGAAAAAATTGATTTTGCTGGGAACTACTATTCTAGTCAAAAAATGTGAATTTTTTTTCTTAAATTTTTGGCAAACTTATCTTTTTGTT
TTATCGGAACCCTCTAGTTTTTTTTTGAAACAAATTTCCAGTCAAAATATATTTTTTCTAGCTTATTACTTAGAATATCCAACAAACT
CTTTTTTACTCCGACTAAATATCTTAGATTATTTTCTATCTACTGATTTTGTTAGCAGGGAATTAAATTCAAAACTTAGCGCTGTTTT
TGTTATTCAATTTTTATCAAAAGAAGGATTATGTGATATAATGGGTAACCCGAAGAGTAAATTAGCATGGCTTAGTTTTACCGACA
ATTCTATTCTTGATAAATATGA

> *Ephedra compacta* 1. (matK). Mexico, Parral, Chih (PARRAL). Parral_1

ATTCTTTCTTTTGAAAGTACTATTTGGTTTATTCGGTTCAAGCTAGATTGTACTATGTGTTATTTTATTTCTAAAAAGAAAGGTTTAG
GAGGTTTTTTCTTGAAAAAAAAATGAAAACATACGTTGGCAACAACATTTTTTATATCCCCTCTTATTCCATAACGATTTCTATGTTA
TAGATCCGAATCTGTTATTCAACTCAAGCCCTTCTTTCGAAAAAATAGAAAAATTACCTAATAGTTTCCGTTTTTTGAATGTAAAAC
GTTCAATTAAGCTATTACATCAACAAAACTATCTTGTGTATAATACAAGAAGTTCTTGTTTTAATTTCATTGGAAAAACTTTTTTTTT
CTGTTTTGATATTTTTGTTTCCACGTGGTGGAAACACTTTTTTGGTTTCAAAGTAACTTTCAAAGAAATAAATCAACAGGTCAATTTT
CAATCAGTCTTTTCTCTATTTCTTTTTTTGGAAGAAGTCTTTATGTTTTCTCTTTCTTTTTCTAATATAAGAATACCCTCTTCGATTCA
TTCAGAGCTGTTAATTAGACGTTTCAAATTTTCGATTCAAGATGTTTCTTTTTTACATTTTTTAAGTTTTATACTTTTTTCAAAGCAAT
TTAAGTTTGTGAATAATTCTATTATTTTTCCAAAAGGAAGTGTGATTTTCTGTTTCTTATTAGGGAATATTCTTCTTTCTATTTTCGAA
GATTTTTTCACTCTTCGATGGAAAAGTTGTTTTCATGAAAAATCATTGTCTTATGGTCTTTTTTCAGAACAAAAGCATTTTCAACAA
AAATGGAATTTTTTAACCCGAAAACCGAAAAAAAAAGACACGATAATATTGCTAAAGGATTTTTTTTTCACTATATAAGATATGG
GGAAAAAATTGATTTTGCTGGGAACTACTATTCTAGTCAAAAAATGTGAATTTTTTTTCTTCAATTTTTGGCAAACTTATCTTTTTGTT
TTATCGGAACCCTCTAGTTTTTTTTTGAAACAAATTTCCAGTCAAAATATATTTTTTCTAGCTTATTACTTAGAATATCCAACAAACT
CTTTTTTACTCCGACTAAATATCTTAGATTATTTTCTATCTACTGATTTTGTTAGCAGGGAATTAAATTCAAAACTTAGCGCTGTTTT
TGTTATTCAATTTTTATCAAAAGAAGGATTATGTGATATAATGGGTAACCCGAAGAGTAAATTAGCATGGCTTAGTTTTACCGACA
ATTCTATTCTTGATAAATATGA

> *Ephedra compacta* 2. (matK). Mexico, Parral, Chih (PARRAL). Parral_2

ATTCTTTCTTTTGAAAGTACTATTTGGTTTATTCGGTTCAAGCTAGATTGTACTATGTGTTATTTTATTTCTAAAAAGAAAGGTTTAG
GAGGTTTTTTCTTGAAAAAAAATGAAAACATACGTTGGCAACAACATTTTTTATATCCCCTCTTATTCCATAACGATTTCTATGTTA
TAGATCCGAATCTGTTATTCAACTCAAGCCCTTCTTTCGAAAAAATAGAAAAATTACCTAATAGTTTCCGTTTTTTGAATGTAAAAC
GTTCAATTAAGCTATTACATCAACAAAACTATCTTGTGTATAATACAAGAAGTTCTTGTTTTAATTTCATTGGAAAAACTTTTTTTTT
CTGTTTTGATATTTTTGTTTCCACGTGGTGGAAACACTTTTTTGGTTTCAAAGTAACTTTCAAAGAAATAAATCAACAGGTCAATTTT
CAATCAGTCTTTTCTCTATTTCTTTTTTTGGAAGAAGTCTTTATGTTTTCTCTTTCTTTTTCTAATATAAGAATACCCTCTTCGATTCA
TTCAGAGCTGTTAATTAGACGTTTCAAATTTTCGATTCAAGATGTTTCTTTTTTACATTTTTTAAGTTTTATACTTTTTTCAAAGCAAT
TTAAGTTTGTGAATAATTCTATTATTTTTCCAAAAGGAAGTGTGATTTTCTGTTTCTTATTAGGGAATATTCTTCTTTCTATTTTCGAA
GATTTTTTCACTCTTCGATGGAAAAGTTGTTTTCATGAAAAATCATTGTCTTATGGTCTTTTTTCAGAACAAAAGCATTTTCAACAA
AAATGGAATTTTTTAACCCGAAAACCGAAAAAAAAAGACACGATAATATTGCTAAAGGATTTTTTTTTTCACTATATAAGATATGG
GGAAAAAATTGATTTTGCTGGGAACTACTATTCTAGTCAAAAAATGTGAATTTTTTTTCTTCAATTTTTGGCAAACTTATCTTTTTGTT
TTATCGGAACCCTCTAGTTTTTTTTTGAAACAAATTTCCAGTCAAAATATATTTTTTCTAGCTTATTACTTAGAATATCCAACAAACT
CTTTTTTACTCCGACTAAATATCTTAGATTATTTTCTATCTACTGATTTTGTTAGCAGGGAATTAAATTCAAAACTTAGCGCTGTTTT
TGTTATTCAATTTTTATCAAAAGAAGGATTATGTGATATAATGGGTAACCCGAAGAGTAAATTAGCATGGCTTAGTTTTACCGACA
ATTCTATTCTTGATAAATATGA
> *Ephedra compacta* 1. (matK). México, Galeana, NL (GAL). Galeana_1
ATTCTTTCTTTTGAAAGTACTATTTGGTTTATTCGGTTCAAGCTAGATTGTACTATGTGTTATTTTATTTCTAAAAAGAAAGGTTTAG
GAGGTTTTTTCTTGAAAAAAAATGAAAACATACGTTGGCAACAACATTTTTTATATCCCCTCTTATTCCATAACGATTTCTATGTTA
TAGATCCGAATCTGTTATTCAACTCAAGCCCTTCTTTCGAAAAAATAGAAAAATTACCTAATAGTTTCCGTTTTTTGAATGTAAAAC
GTTCAATTAAGCTATTACATCAACAAAACTATCTTGTGTATAATACAAGAAGTTCTTGTTTTAATTTCATTGGAAAAACTTTTTTTTT
CTGTTTTGATATTTTTGTTTCCACGTGGTGGAAACACTTTTTTGGTTTCAAAGTAACTTTCAAAGAAATAAATCAACAGGTCAATTTT
CAATCAGTCTTTTCTCTATTTCTTTTTTTGGAAGAAGTCTTTATGTTTTCTCTTTCTTTTTCTAATATAAGAATACCCTCTTCGATTCA
TTCAGAGCTGTTAATTAGACGTTTCAAATTTTCGATTCAAGATGTTTCTTTTTTACATTTTTTAAGTTTTATACTTTTTTCAAAGCAAT
TTAAGTTTGTGAATAATTCTATTATTTTTCCAAAAGGAAGTGTGATTTTCTGTTTCTTATTAGGGAATATTCTTCTTTCTATTTTCGAA
GATTTTTTCACTCTTCGATGGAAAAGTTGTTTTCATGAAAAATCATTGTCTTATGGTCTTTTTTCAGAACAAAAGCATTTTCAACAA
AAATGGAATTTTTTAACCCGAAAACCGAAAAAAAAAGACACGATAATATTGCTAAAGGATTTTTTTTTTCACTATATAAGATATGG
GGAAAAAATTGATTTTGCTGGGAACTACTATTCTAGTCAAAAAATGTGAATTTTTTTTCTTAAATTTTTGGCAAACTTATCTTTTTGTT
TTATCGGAACCCTCTAGTTTTTTTTTGAAACAAATTTCCAGTCAAAATATGTTTTTTCTAGCTTATTACTTAGAATATCCAACAAACT
CTTTTTTACTCCGACTAAATATCTTAGATTATTTTCTATCTACTGATTTTGTTAGCAGGGAATTAAATTCAAAACTTAGCGCTGTTTT
TGTTATTCAATTTTTATCAAAAGAAGGATTATGTGATATAATGGGTAACCCGAAGAGTAAATTAGCATGGCTTAGTTTTACCGACA
ATTCTATTCTTGATAAATATGA
> *Ephedra compacta* 3. (matK). México, Galeana, NL (GAL). Galeana_3
ATTCTTTCTTTTGAAAGTACTATTTGGTTTATTCGGTTCAAGCTAGATTGTACTATGTGTTATTTTATTTCTAAAAAGAAAGGTTTAG
GAGGTTTTTTCTTGAAAAAAAATGAAAACATACGTTGGCAACAACATTTTTTATATCCCCTCTTATTCCATAACGATTTCTATGTTA
TAGATCCGAATCTGTTATTCAACTCAAGCCCTTCTTTCGAAAAAATAGAAAAATTACCTAATAGTTTCCGTTTTTTGAATGTAAAAC
GTTCAATTAAGCTATTACATCAACAAAACTATCTTGTGTATAATACAAGAAGTTCTTGTTTTAATTTCATTGGAAAAACTTTTTTTTT
CTGTTTTGATATTTTTGTTTCCACGTGGTGGAAACACTTTTTTGGTTTCAAAGTAACTTTCAAAGAAATAAATCAACAGGTCAATTTT
CAATCAGTCTTTTCTCTATTTCTTTTTTTGGAAGAAGTCTTTATGTTTTCTCTTTCTTTTTCTAATATAAGAATACCCTCTTCGATTCA
TTCAGAGCTGTTAATTAGACGTTTCAAATTTTCGATTCAAGATGTTTCTTTTTTACATTTTTTAAGTTTTATACTTTTTTCAAAGCAAT
TTAAGTTTGTGAATAATTCTATTATTTTTCCAAAAGGAAGTGTGATTTTCTGTTTCTTATTAGGGAATATTCTTCTTTCTATTTTCGAA
GATTTTTTCACTCTTCGATGGAAAAGTTGTTTTCATGAAAAATCATTGTCTTATGGTCTTTTTTCAGAACAAAAGCATTTTCAACAA
AAATGGAATTTTTTAACCCGAAAACCGAAAAAAAAAGACACGATAATATTGCTAAAGGATTTTTTTTTTCACTATATAAGATATGG

GGAAAAATTGATTTTGCTGGGAACTACTATTCTAGTCAAAAAATGTGAATTTTTTTTCTTAAATTTTTGGCAAACTTATCTTTTTGTT
TTATCGGAACCCTCTAGTTTTTTTTTGAAACAAATTTCCAGTCAAAATATGTTTTTTCTAGCTTATTACTTAGAATATCCAACAAACT
CTTTTTTACTCCGACTAAATATCTTAGATTATTTTCTATCTACTGATTTTGTTAGCAGGGAATTAAATTCAAAACTTAGCGCTGTTTT
TGTTATTCAATTTTTATCAAAAGAAGGATTATGTGATATAATGGGTAACCCGAAGAGTAAATTAGCATGGCTTAGTTTTACCGACA
ATTCTATTCTTGATAAATATGA

> ***Ephedra compacta*** **4. (matK). México, Galeana, NL (GAL).** Galeana_4
ATTCTTTCTTTTGAAAGTACTATTTGGTTTATTCGGTTCAAGCTAGATTGTACTATGTGTTATTTTATTTCTAAAAAGAAAGGTTTAG
GAGGTTTTTTCTTGAAAAAAAAATGAAAACATACGTTGGCAACAACATTTTTTATATCCCCTCTTATTCCATAACGATTTCTATGTTA
TAGATCCGAATCTGTTATTCAACTCAAGCCCTTCTTTCGAAAAAATAGAAAAATTACCTAATAGTTTCCGTTTTTTGAATGTAAAAC
GTTCAATTAAGCTATTACATCAACAAAACTATCTTGTGTATAATACAAGAAGTTCTTGTTTTAATTTCATTGGAAAAACTTTTTTTTT
CTGTTTTGATATTTTTGTTTCCACGTGGTGGAAACACTTTTTTGGTTTCAAAGTAACTTTCAAAGAAATAAATCAACAGGTCAATTTT
CAATCAGTCTTTTCTCTATTTCTTTTTTTGGAAGAAGTCTTTATGTTTTCTCTTTCTTTTTCTAATATAAGAATACCCTCTTCGATTCA
TTCAGAGCTGTTAATTAGACGTTTCAAATTTTCGATTCAAGATGTTTCTTTTTTACATTTTTTAAGTTTTATACTTTTTTCAAAGCAAT
TTAAGTTTGTGAATAATTCTATTATTTTTCCAAAAGGAAGTGTGATTTTCTGTTTCTTATTAGGGAATATTCTTCTTTCTATTTTCGAA
GATTTTTTCACTCTTCGATGGAAAAGTTGTTTTCATGAAAAATCATTGTCTTATGGTCTTTTTTCAGAACAAAAGCATTTTCAACAA
AAATGGAATTTTTTAACCCGAAAACCGAAAAAAAAAGACACGATAATATTGCTAAAGGATTTTTTTTTTCACTATATAAGATATGG
GGAAAAATTGATTTTGCTGGGAACTACTATTCTAGTCAAAAAATGTGAATTTTTTTTCTTAAATTTTTGGCAAACTTATCTTTTTGTT
TTATCGGAACCCTCTAGTTTTTTTTTGAAACAAATTTCCAGTCAAAATATGTTTTTTCTAGCTTATTACTTAGAATATCCAACAAACT
CTTTTTTACTCCGACTAAATATCTTAGATTATTTTCTATCTACTGATTTTGTTAGCAGGGAATTAAATTCAAAACTTAGCGCTGTTTT
TGTTATTCAATTTTTATCAAAAGAAGGATTATGTGATATAATGGGTAACCCGAAGAGTAAATTAGCATGGCTTAGTTTTACCGACA
ATTCTATTCTTGATAAATATGA

> ***Ephedra compacta*** **5. (matK). México, Galeana, NL (GAL).** Galeana_5
ATTCTTTCTTTTGAAAGTACTATTTGGTTTATTCGGTTCAAGCTAGATTGTACTATGTGTTATTTTATTTCTAAAAAGAAAGGTTTAG
GAGGTTTTTTCTTGAAAAAAAAATGAAAACATACGTTGGCAACAACATTTTTTATATCCCCTCTTATTCCATAACGATTTCTATGTTA
TAGATCCGAATCTGTTATTCAACTCAAGCCCTTCTTTCGAAAAAATAGAAAAATTACCTAATAGTTTCCGTTTTTTGAATGTAAAAC
GTTCAATTAAGCTATTACATCAACAAAACTATCTTGTGTATAATACAAGAAGTTCTTGTTTTAATTTCATTGGAAAAACTTTTTTTTT
CTGTTTTGATATTTTTGTTTCCACGTGGTGGAAACACTTTTTTGGTTTCAAAGTAACTTTCAAAGAAATAAATCAACAGGTCAATTTT
CAATCAGTCTTTTCTCTATTTCTTTTTTTGGAAGAAGTCTTTATGTTTTCTCTTTCTTTTTCTAATATAAGAATACCCTCTTCGATTCA
TTCAGAGCTGTTAATTAGACGTTTCAAATTTTCGATTCAAGATGTTTCTTTTTTACATTTTTTAAGTTTTATACTTTTTTCAAAGCAAT
TTAAGTTTGTGAATAATTCTATTATTTTTCCAAAAGGAAGTGTGATTTTCTGTTTCTTATTAGGGAATATTCTTCTTTCTATTTTCGAA
GATTTTTTCACTCTTCGATGGAAAAGTTGTTTTCATGAAAAATCATTGTCTTATGGTCTTTTTTCAGAACAAAAGCATTTTCAACAA
AAATGGAATTTTTTAACCCGAAAACCGAAAAAAAAAGACACGATAATATTGCTAAAGGATTTTTTTTTTCACTATATAAGATATGG
GGAAAAATTGATTTTGCTGGGAACTACTATTCTAGTCAAAAAATGTGAATTTTTTTTCTTAAATTTTTGGCAAACTTATCTTTTTGTT
TTATCGGAACCCTCTAGTTTTTTTTTGAAACAAATTTCCAGTCAAAATATGTTTTTTCTAGCTTATTACTTAGAATATCCAACAAACT
CTTTTTTACTCCGACTAAATATCTTAGATTATTTTCTATCTACTGATTTTGTTAGCAGGGAATTAAATTCAAAACTTAGCGCTGTTTT
TGTTATTCAATTTTTATCAAAAGAAGGATTATGTGATATAATGGGTAACCCGAAGAGTAAATTAGCATGGCTTAGTTTTACCGACA
ATTCTATTCTTGATAAATATGA

> ***Ephedra compacta*** **6. (matK). México, Galeana, NL (GAL).** Galeana_6
ATTCTTTCTTTTGAAAGTACTATTTGGTTTATTCGGTTCAAGCTAGATTGTACTATGTGTTATTTTATTTCTAAAAAGAAAGGTTTAG
GAGGTTTTTTCTTGAAAAAAAAATGAAAACATACGTTGGCAACAACATTTTTTATATCCCCTCTTATTCCATAACGATTTCTATGTTA
TAGATCCGAATCTGTTATTCAACTCAAGCCCTTCTTTCGAAAAAATAGAAAAATTACCTAATAGTTTCCGTTTTTTGAATGTAAAAC
GTTCAATTAAGCTATTACATCAACAAAACTATCTTGTGTATAATACAAGAATTTCTTGTTTTAATTTCATTGGAAAAACTTTTTTTTT

CTGTTTTGATATTTTTGTTTCCACGTGGTGGAAACACTTTTTTGGTTTCAAAGTAACTTTCAAAGAAATAAATCAACAGGTCAATTTT
CAATCAGTCTTTTCTCTATTTCTTTTTTTGGAAGAAGTCTTTATGTTTTCTCTTTCTTTTTCTAATATAAGAATACCCTCTTCGATTCA
TTCAGAGCTGTTAATTAGACGTTTCAAATTTTCGATTCAAGATGTTTCTTTTTTACATTTTTTAAGTTTTATACTTTTTTCAAAGCAAT
TTAAGTTTGTGAATAATTCTATTATTTTTCCAAAAGGAAGTGTGATTTTCTGTTTCTTATTAGGGAATATTCTTCTTTCTATTTTCGAA
GATTTTTTCACTCTTCGATGGAAAAGTTGTTTTCATGAAAAATCATTGTCTTATGGTCTTTTTTCAGAACAAAAGCATTTTCAACAA
AAATGGAATTTTTTAACCCGAAAACCGAAAAAAAAAGACACGATAATATTGCTAAAGGATTTTTTTTTCACTATATAAGATATGG
GGAAAAAATTGATTTTGCTGGGAACTACTATTCTAGTCAAAAAATGTGAATTTTTTTTCTTAAATTTTGGCAAACTTATCTTTTTGTT
TTATCGGAACCCTCTAGTTTTTTTTTGAAACAAATTTCCAGTCAAAATATGTTTTTTCTAGCTTATTACTTAGAATATCCAACAAACT
CTTTTTTACTCCGACTAAATATCTTAGATTATTTTCTATCTACTGATTTTGTTAGCAGGGAATTAAATTCAAAACTTAGCGCTGTTTT
TGTTATTCAATTTTTATCAAAAGAAGGATTATGTGATATAATGGGTAACCCGAAGAGTAAATTAGCATGGCTTAGTTTTACCGACA
ATTCTATTCTTGATAAATATGA

> *Ephedra compacta* 1 (psbB). Mexico, Arteaga, Coah (ART). Sierra_Art_1

TCATTTCACACTATAAGTCAGCCCAGTCCAAAACTTCTTCTTTTGTTTCTTTTTTCTTTTTATTATCTTTGTTTTCCGGCTCTTTATCCT
CTCCTGTTTCGTTATCTTTTGCCAAAGTCATAATAGGTGCCTCATGCATTATATTATTCTTAGTTTTTTTTGATCCTAGAACCGGAAA
AGTACATGGGGTCTCATTCTTTCGTTTAGGCTTAGGTATTGTTGTCTCTTCAAAAAAGTGATTAAATTGAATCTCTTGAGGAGTTCT
ATCATCTTCTTTTCTTGACGGAGGGTTATCTTCACCAAAAAAACGAACTTTTGGGATACCAAGGGGCATTTTTTTTAGATCCTTTTTT
TTCTCTTTTTATTCTCCTTCTTCTCTTTCTTTTTGTTTTCCAAATTTTGTAAGTATTTTTTGTTTTGAATGGTACCAATCCTTCAATTTT
GTAAATTGAAACATAAAAGATAAAATATTTTTGCTTCTCCTACCGGTGTTTTTATTCAACATAGTTTTATAAAAGGAAGGATGATCC
AACCTAAAATTCAGTGTGTTTTTATAATGTAAGAAAGTTCAATCTTTTTTTTTTGAAAAAGAGGTGTTTAATGGGTTTACCTTGGTA
TCGTGTGCATACTGTTGTCTTGAATGATCCTGGCCGGTTAATTTCTGTGCATATAATGCATACAGCTTTAGTAGCAGGTTGGGCCGG
TTCAAT

> *Ephedra compacta* 2 (psbB). Mexico, Arteaga, Coah (ART). Sierra_Art_2

TCATTTCACACTATAAGTCAGCCCAGTCCAAAACTTCTTCTTTTGTTTCTTTTTTCTTTTTATTATCTTTGTTTTCCGGCTCTTTATCCT
CTCCTGTTTCGTTATCTTTTGCCAAAGTCATAATAGGTGCCTCATGCATTATATTATTCTTAGTTTTTTTTGATCCTAGAACCGGAAA
AGTACATGGGGTCTCATTCTTTCGTTTAGGCTTAGGTATTGTTGTCTCTTCAAAAAAGTGATTAAATTGAATCTCTTGAGGAGTTCT
ATCATCTTCTTTTCTTGACGGAGGGTTATCTTCACCAAAAAAACGAACTTTTGGGATACCAAGGGGCATTTTTTTTAGATCCTTTTTT
TTCTCTTTTTATTCTCCTTCTTCTCTTTCTTTTTGTTTTCCAAATTTTGTAAGTATTTTTTGTTTTGAATGGTACCAATCCTTCAATTTT
GTAAATTGAAACATAAAAGATAAAATATTTTTGCTTCTCCTACCGGTGTTTTTATTCAACATAGTTTTATAAAAGGAAGGATGATCC
AACCTAAAATTCAGTGTGTTTTTATAATGTAAGAAAGTTCAATCTTTTTTTTTTGAAAAAGAGGTGTTTAATGGGTTTACCTTGGTA
TCGTGTGCATACTGTTGTCTTGAATGATCCTGGCCGGTTAATTTCTGTGCATATAATGCATACAGCTTTAGTAGCAGGTTGGGCCGG
TTCAAT

> *Ephedra compacta* 4 (psbB). Mexico, Arteaga, Coah (ART). Sierra_Art_4

TCATTTCACACTATAAGTCAGCCCAGTCCAAAACTTCTTCTTTTGTTTCTTTTTTCTTTTTATTATCTTTGTTTTCCGGCTCTTTATCCT
CTCCTGTTTCGTTATCTTTTGCCAAAGTCATAATAGGTGCCTCATGCATTATATTATTCTTAGTTTTTTTTGATCCTAGAACCGGAAA
AGTACATGGGGTCTCATTCTTTCGTTTAGGCTTAGGTATTGTTGTCTCTTCAAAAAAGTGATTAAATTGAATCTCTTGAGGAGTTCT
ATCATCTTCTTTTCTTGACGGAGGGTTATCTTCACCAAAAAAACGAACTTTTGGGATACCAAGGGGCATTTTTTTTAGATCCTTTTTT
TTCTCTTTTTATTCTCCTTCTTCTCTTTCTTTTTGTTTTCCAAATTTTGTAAGTATTTTTTGTTTTGAATGGTACCAATCCTTCAATTTT
GTAAATTGAAACATAAAAGATAAAATATTTTTGCTTCTCCTACCGGTGTTTTTATTCAACATAGTTTTATAAAAGGAAGGATGATCC
AACCTAAAATTCAGTGTGTTTTTATAATGTAAGAAAGTTCAATCTTTTTTTTTTGAAAAAGAGGTGTTTAATGGGTTTACCTTGGTA
TCGTGTGCATACTGTTGTCTTGAATGATCCTGGCCGGTTAATTTCTGTGCATATAATGCATACAGCTTTAGTAGCAGGTTGGGCCGG
TTCAAT

> *Ephedra compacta* 5 (psbB). Mexico, Arteaga, Coah (ART). Sierra_Art_5

TCATTTCACACTATAAGTCAGCCCAGTCCAAAACTTCTTCTTTTGTTTCTTTTTTCTTTTTATTATCTTTGTTTTCCGGCTCTTTATCCT
CTCCTGTTTCGTTATCTTTTGCCAAAGTCATAATAGGTGCCTCATGCATTATATTATTCTTAGTTTTTTTTGATCCTAGAACCGGAAA
AGTACATGGGGTCTCATTCTTTCGTTTAGGCTTAGGTATTGTTGTCTCTTCAAAAAAGTGATTAAATTGAATCTCTTGAGGAGTTCT
ATCATCTTCTTTTCTTGACGGAGGGTTATCTTCACCAAAAAAACGAACTTTTGGGATACCAAGGGGCATTTTTTTTAGATCCTTTTTT
TTCTCTTTTTATTCTCCTTCTTCTCTTTCTTTTTGTTTTCCAAATTTTGTAAGTATTTTTTGTTTTGAATGGTACCAATCCTTCAATTTT
GTAAATTGAAACATAAAAGATAAAATATTTTTGCTTCTCCTACCGGTGTTTTTATTCAACATAGTTTTATAAAAGGAAGGATGATCC
AACCTAAAATTCAGTGTGTTTTTATAATGTAAGAAAGTTCAATCTTTTTTTTTTGAAAAAGAGGTGTTTAATGGGTTTACCTTGGTA
TCGTGTGCATACTGTTGTCTTGAATGATCCTGGCCGGTTAATTTCTGTGCATATAATGCATACAGCTTTAGTAGCAGGTTGGGCCGG
TTCAAT

> ***Ephedra compacta*** **6 (psbB). Mexico, Arteaga, Coah (ART).** Sierra_Art_6
TCATTTCACACTATAAGTCAGCCCAGTCCAAAACTTCTTCTTTTGTTTCTTTTTTCTTTTTATTATCTTTGTTTTCCGGCTCTTTATCCT
CTCCTGTTTCGTTATCTTTTGCCAAAGTCATAATAGGTGCCTCATGCATTATATTATTCTTAGTTTTTTTTGATCCTAGAACCGGAAA
AGTACATGGGGTCTCATTCTTTCGTTTAGGCTTAGGTATTGTTGTCTCTTCAAAAAAGTGATTAAATTGAATCTCTTGAGGAGTTCT
ATCATCTTCTTTTCTTGACGGAGGGTTATCTTCACCAAAAAAACGAACTTTTGGGATACCAAGGGGCATTTTTTTTAGATCCTTTTTT
TTCTCTTTTTATTCTCCTTCTTCTCTTTCTTTTTGTTTTCCAAATTTTGTAAGTATTTTTTGTTTTGAATGGTACCAATCCTTCAATTTT
GTAAATTGAAACATAAAAGATAAAATATTTTTGCTTCTCCTACCGGTGTTTTTATTCAACATAGTTTTATAAAAGGAAGGATGATCC
AACCTAAAATTCAGTGTGTTTTTATAATGTAAGAAAGTTCAATCTTTTTTTTTTGAAAAAGAGGTGTTTAATGGGTTTACCTTGGTA
TCGTGTGCATACTGTTGTCTTGAATGATCCTGGCCGGTTAATTTCTGTGCATATAATGCATACAGCTTTAGTAGCAGGTTGGGCCGG
TTCAAT

> ***Ephedra compacta*** **2 (psbB). Mexico, borderline Coah-Zac (FCZ).** Coah_Zac_2
TCATTTCACACTATAAGTCAGCCCAGTCCAAAACTTCTTCTTTTGTTTCTTTTTTCTTTTTATTATCTTTGTTTTCCGGCTCTTTATCCT
CTCCTGTTTCGTTATCTTTTGCCAAAGTCATAATAGGTGCCTCATGCATTATATTATTCTTAGTTTTTTTTGATCCTAGAACCGGAAA
AGTACATGGGGTCTCATTCTTTCGTTTAGGCTTAGGTATTGTTGTCTCTTCAAAAAAGTGATTAAATTGAATCTCTTGAGGAGTTCT
ATCATCTTCTTTTCTTGACGGAGGGTTATCTTCACCAAAAAAACGAACTTTTGGGATACCAAGGGGCATTTTTTTTAGATCCTTTTTT
TTCTCTTTTTATTCTCCTTCTTCTCTTTCTTTTTGTTTTCCAAATTTTGTAAGTATTTTTTGTTTTGAATGGTACCAATCCTTCAATTTT
GTAAATTGAAACATAAAAGATAAAATATTTTTGCTTCTCCTACCGGTGTTTTTATTCAACATAGTTTTATAAAAGGAAGGATGATCC
AACCTAAAATTCAGTGTGTTTTTATAATGTAAGAAAGTTCAATCTTTTTTTTTTGAAAAAGAGGTGTTTAATGGGTTTACCTTGGTAT
CGTGTGCATACTGTTGTCTTGAATGATCCTGGCCGGTTAATTTCTGTGCATATAATGCATACAGCTTTAGTAGCAGGTTGGGCCGGT
TCAAC

> ***Ephedra compacta*** **3 (psbB). Mexico, borderline Coah-Zac (FCZ).** Coah_Zac_3
TCATTTCACACTATAAGTCAGCCCAGTCCAAAACTTCTTCTTTTGTTTCTTTTTTCTTTTTATTATCTTTGTTTTCCGGCTCTTTATCCT
CTCCTGTTTCGTTATCTTTTGCCAAAGTCATAATAGGTGCCTCATGCATTATATTATTCTTAGTTTTTTTTGATCCTAGAACCGGAAA
AGTACATGGGGTCTCATTCTTTCGTTTAGGCTTAGGTATTGTTGTCTCTTCAAAAAAGTGATTAAATTGAATCTCTTGAGGAGTTCT
ATCATCTTCTTTTCTTGACGGAGGGTTATCTTCACCAAAAAAACGAACTTTTGGGATACCAAGGGGCATTTTTTTTAGATCCTTTTTT
TTCTCTTTTTATTCTCCTTCTTCTCTTTCTTTTTGTTTTCCAAATTTTGTAAGTATTTTTTGTTTTGAATGGTACCAATCCTTCAATTTT
GTAAATTGAAACATAAAAGATAAAATATTTTTGCTTCTCCTACCGGTGTTTTTATTCAACATAGTTTTATAAAAGGAAGGATGATCC
AACCTAAAATTCAGTGTGTTTTTATAATGTAAGAAAGTTCAATCTTTTTTTTTTGAAAAAGAGGTGTTTAATGGGTTTACCTTGGTAT
CGTGTGCATACTGTTGTCTTGAATGATCCTGGCCGGTTAATTTCTGTGCATATAATGCATACAGCTTTAGTAGCAGGTTGGGCCGGT
TCAAC

> ***Ephedra compacta*** **4 (psbB). Mexico, borderline Coah-Zac (FCZ).** Coah_Zac_4
TCATTTCACACTATAAGTCAGCCCAGTCCAAAACTTCTTCTTTTGTTTCTTTTTTCTTTTTATTATCTTTGTTTTCCGGCTCTTTATCCT
CTCCTGTTTCGTTATCTTTTGCCAAAGTCATAATAGGTGCCTCATGCATTATATTATTCTTAGTTTTTTTTGATCCTAGAACCGGAAA

AGTACATGGGGTCTCATTCTTTCGTTTAGGCTTAGGTATTGTTGTCTCTTCAAAAAAGTGATTAAATTGAATCTCTTGAGGAGTTCT
ATCATCTTCTTTTCTTGACGGAGGGTTATCTTCACCAAAAAAACGAACTTTTGGGATACCAAGGGGCATTTTTTTTAGATCCTTTTTT
TTCTCTTTTTATTCTCCTTCTTCTCTTTCTTTTTGTTTTCCAAATTTTGTAAGTATTTTTTGTTTTGAATGGTACCAATCCTTCAATTTT
GTAAATTGAAACATAAAAGATAAAATATTTTTGCTTCTCCTACCGGTGTTTTTATTCAACATAGTTTTATAAAAGGAAGGATGATCC
AACCTAAAATTCAGTGTGTTTTTATAATGTAAGAAAGTTCAATCTTTTTTTTTTGAAAAAGAGGTGTTTAATGGGTTTACCTTGGTA
TCGTGTGCATACTGTTGTCTTGAATGATCCTGGCCGGTTAATTTCTGTGCATATAATGCATACAGCTTTAGTAGCAGGTTGGGCCGG
TTCAAT

> *Ephedra compacta* 5 (psbB). Mexico, borderline Coah-Zac (FCZ). Coah_Zac_5
TCATTTCACACTATAAGTCAGCCCAGTCCAAAACTTCTTCTTTTGTTTCTTTTTTCTTTTTATTATCTTTGTTTTCCGGCTCTTTATCCT
CTCCTGTTTCGTTATCTTTTGCCAAAGTCATAATAGGTGCCTCATGCATTATATTATTCTTAGTTTTTTTTGATCCTAGAACCGGAAA
AGTACATGGGGTCTCATTCTTTCGTTTAGGCTTAGGTATTGTTGTCTCTTCAAAAAAGTGATTAAATTGAATCTCTTGAGGAGTTCT
ATCATCTTCTTTTCTTGACGGAGGGTTATCTTCACCAAAAAAACGAACTTTTGGGATACCAAGGGGCATTTTTTTTAGATCCTTTTTT
TTCTCTTTTTATTCTCCTTCTTCTCTTTCTTTTTGTTTTCCAAATTTTGTAAGTATTTTTTGTTTTGAATGGTACCAATCCTTCAATTTT
GTAAATTGAAACATAAAAGATAAAATATTTTTGCTTCTCCTACCGGTGTTTTTATTCAACATAGTTTTATAAAAGGAAGGATGATCC
AACCTAAAATTCAGTGTGTTTTTATAATGTAAGAAAGTTCAATCTTTTTTTTTTGAAAAAGAGGTGTTTAATGGGTTTACCTTGGTAT
CGTGTGCATACTGTTGTCTTGAATGATCCTGGCCGGTTAATTTCTGTGCATATAATGCATACAGCTTTAGTAGCAGGTTGGGCCGGT
TCAAC

> *Ephedra compacta* 6 (psbB). Mexico, borderline Coah-Zac (FCZ). Coah_Zac_6
TCATTTCACACTATAAGTCAGCCCAGTCCAAAACTTCTTCTTTTGTTTCTTTTTTCTTTTTATTATCTTTGTTTTCCGGCTCTTTATCCT
CTCCTGTTTCGTTATCTTTTGCCAAAGTCATAATAGGTGCCTCATGCATTATATTATTCTTAGTTTTTTTTGATCCTAGAACCGGAAA
AGTACATGGGGTCTCATTCTTTCGTTTAGGCTTAGGTATTGTTGTCTCTTCAAAAAAGTGATTAAATTGAATCTCTTGAGGAGTTCT
ATCATCTTCTTTTCTTGACGGAGGGTTATCTTCACCAAAAAAACGAACTTTTGGGATACCAAGGGGCATTTTTTTTAGATCCTTTTTT
TTCTCTTTTTATTCTCCTTCTTCTCTTTCTTTTTGTTTTCCAAATTTTGTAAGTATTTTTTGTTTTGAATGGTACCAATCCTTCAATTTT
GTAAATTGAAACATAAAAGATAAAATATTTTTGCTTCTCCTACCGGTGTTTTTATTCAACATAGTTTTATAAAAGGAAGGATGATCC
AACCTAAAATTCAGTGTGTTTTTATAATGTAAGAAAGTTCAATCTTTTTTTTTTGAAAAAGAGGTGTTTAATGGGTTTACCTTGGTAT
CGTGTGCATACTGTTGTCTTGAATGATCCTGGCCGGTTAATTTCTGTGCATATAATGCATACAGCTTTAGTAGCAGGTTGGGCCGGT
TCAAC

> *Ephedra compacta* 7 (psbB). Mexico, borderline Coah-Zac (FCZ). Coah_Zac_7
TCATTTCACACTATAAGTCAGCCCAGTCCAAAACTTCTTCTTTTGTTTCTTTTTTCTTTTTATTATCTTTGTTTTCCGGCTCTTTATCCT
CTCCTGTTTCGTTATCTTTTGCCAAAGTCATAATAGGTGCCTCATGCATTATATTATTCTTAGTTTTTTTTGATCCTAGAACCGGAAA
AGTACATGGGGTCTCATTCTTTCGTTTAGGCTTAGGTATTGTTGTCTCTTCAAAAAAGTGATTAAATTGAATCTCTTGAGGAGTTCT
ATCATCTTCTTTTCTTGACGGAGGGTTATCTTCACCAAAAAAACGAACTTTTGGGATACCAAGGGGCATTTTTTTTAGATCCTTTTTT
TTCTCTTTTTATTCTCCTTCTTCTCTTTCTTTTTGTTTTCCAAATTTTGTAAGTATTTTTTGTTTTGAATGGTACCAATCCTTCAATTTT
GTAAATTGAAACATAAAAGATAAAATATTTTTGCTTCTCCTACCGGTGTTTTTATTCAACATAGTTTTATAAAAGGAAGGATGATCC
AACCTAAAATTCAGTGTGTTTTTATAATGTAAGAAAGTTCAATCTTTTTTTTTTGAAAAAGAGGTGTTTAATGGGTTTACCTTGGTA
TCGTGTGCATACTGTTGTCTTGAATGATCCTGGCCGGTTAATTTCTGTGCATATAATGCATACAGCTTTAGTAGCAGGTTGGGCCGG
TTCAAT

> *Ephedra compacta* 9 (psbB). Mexico, borderline Coah-Zac (FCZ). Coah_Zac_9
TCATTTCACACTATAAGTCAGCCCAGTCCAAAACTTCTTCTTTTGTTTCTTTTTTCTTTTTATTATCTTTGTTTTCCGGCTCTTTATCCT
CTCCTGTTTCGTTATCTTTTGCCAAAGTCATAATAGGTGCCTCATGCATTATATTATTCTTAGTTTTTTTTGATCCTAGAACCGGAAA
AGTACATGGGGTCTCATTCTTTCGTTTAGGCTTAGGTATTGTTGTCTCTTCAAAAAAGTGATTAAATTGAATCTCTTGAGGAGTTCT
ATCATCTTCTTTTCTTGACGGAGGGTTATCTTCACCAAAAAAACGAACTTTTGGGATACCAAGGGGCATTTTTTTTAGATCCTTTTTT

TTCTCTTTTTATTCTCCTTCTTCTCTTTCTTTTTGTTTTCCAAATTTTGTAAGTATTTTTTGTTTTGAATGGTACCAATCCTTCAATTTT
GTAAATTGAAACATAAAAGATAAAATATTTTTGCTTCTCCTACCGGTGTTTTTATTCAACATAGTTTTATAAAAGGAAGGATGATCC
AACCTAAAATTCAGTGTGTTTTTATAATGTAAGAAAGTTCAATCTTTTTTTTTGAAAAAGAGGTGTTTAATGGGTTTACCTTGGTAT
CGTGTGCATACTGTTGTCTTGAATGATCCTGGCCGGTTAATTTCTGTGCATATAATGCATACAGCTTTAGTAGCAGGTTGGGCCGGT
TCAAC
> *Ephedra compacta* 1 (psbB). Mexico, Sierra Mojada, Coah (MOJ). Sierra_Moj_1
TCATTTCACACTATAAGTCAGCCCAGTCCAAAACTTCTTCTTTTGTTTCTTTTTTCTTTTTATTATCTTTGTTTTCCGGCTCTTTATCCT
CTCCTGTTTCGTTATCTTTTGCCAAAGTCATAATAGGTGCCTCATGCATTATATTATTCTTAGTTTTTTTTGATCCTAGAACCGGAAA
AGTACATGGGGTCTCATTCTTTCGTTTAGGCTTAGGTATTGTTGTCTCTTCAAAAAAGTGATTAAATTGAATCTCTTGAGGAGTTCT
ATCATCTTCTTTTCTTGACGGAGGGTTATCTTCACCAAAAAAACGAACTTTTGGGATACCAAGGGGCATTTTTTTTAGATCCTTTTTT
TTCTCTTTTTATTCTCCTTCTTCTCTTTCTTTTTGTTTTCCAAATTTTGTAAGTATTTTTTGTTTTGAATGGTACCAATCCTTAAATTTT
GTAAATTGAAACATAAAAGATAAAATATTTTTGCTTCTCCTACCGGTGTTTTTATTCAACATAGTTTTATAAAAGGAAGGATGATCC
AACCTAAAATTCAGTGTGTTTTTATAATGTAAGAAAGTTCAATCTTTTTTTTTTGAAAAAGAGGTGTTTAATGGGTTTACCTTGGTA
TCGTGTGCATACTGTTGTCTTGAATGATCCTGGCCGGTTAATTTCTGTGCATATAATGCATACAGCTTTAGTAGCAGGTTGGGCCGG
TTCAAT
> *Ephedra compacta* 2 (psbB). Mexico, Sierra Mojada, Coah (MOJ). Sierra_Moj_2
TCATTTCACACTATAAGTCAGCCCAGTCCAAAACTTCTTCTTTTGTTTCTTTTTTCTTTTTATTATCTTTGTTTTCCGGCTCTTTATCCT
CTCCTGTTTCGTTATCTTTTGCCAAAGTCATAATAGGTGCCTCATGCATTATATTATTCTTAGTTTTTTTTGATCCTAGAACCGGAAA
AGTACATGGGGTCTCATTCTTTCGTTTAGGCTTAGGTATTGTTGTCTCTTCAAAAAAGTGATTAAATTGAATCTCTTGAGGAGTTCT
ATCATCTTCTTTTCTTGACGGAGGGTTATCTTCACCAAAAAAACGAACTTTTGGGATACCAAGGGGCATTTTTTTTAGATCCTTTTTT
TTCTCTTTTTATTCTCCTTCTTCTCTTTCTTTTTGTTTTCCAAATTTTGTAAGTATTTTTTGTTTTGAATGGTACCAATCCTTAAATTTT
GTAAATTGAAACATAAAAGATAAAATATTTTTGCTTCTCCTACCGGTGTTTTTATTCAACATAGTTTTATAAAAGGAAGGATGATCC
AACCTAAAATTCAGTGTGTTTTTATAATGTAAGAAAGTTCAATCTTTTTTTTTTGAAAAAGAGGTGTTTAATGGGTTTACCTTGGTA
TCGTGTGCATACTGTTGTCTTGAATGATCCTGGCCGGTTAATTTCTGTGCATATAATGCATACAGCTTTAGTAGCAGGTTGGGCCGG
TTCAAT
> *Ephedra compacta* 3 (psbB). Mexico, Sierra Mojada, Coah (MOJ). Sierra_Moj_3
TCATTTCACACTATAAGTCAGCCCAGTCCAAAACTTCTTCTTTTGTTTCTTTTTTCTTTTTATTATCTTTGTTTTCCGGCTCTTTATCCT
CTCCTGTTTCGTTATCTTTTGCCAAAGTCATAATAGGTGCCTCATGCATTATATTATTCTTAGTTTTTTTTGATCCTAGAACCGGAAA
AGTACATGGGGTCTCATTCTTTCGTTTAGGCTTAGGTATTGTTGTCTCTTCAAAAAAGTGATTAAATTGAATCTCTTGAGGAGTTCT
ATCATCTTCTTTTCTTGACGGAGGGTTATCTTCACCAAAAAAACGAACTTTTGGGATACCAAGGGGCATTTTTTTTAGATCCTTTTTT
TTCTCTTTTTATTCTCCTTCTTCTCTTTCTTTTTGTTTTCCAAATTTTGTAAGTATTTTTTGTTTTGAATGGTACCAATCCTTAAATTTT
GTAAATTGAAACATAAAAGATAAAATATTTTTGCTTCTCCTACCGGTGTTTTTATTCAACATAGTTTTATAAAAGGAAGGATGATCC
AACCTAAAATTCAGTGTGTTTTTATAATGTAAGAAAGTTCAATCTTTTTTTTTTGAAAAAGAGGTGTTTAATGGGTTTACCTTGGTA
TCGTGTGCATACTGTTGTCTTGAATGATCCTGGCCGGTTAATTTCTGTGCATATAATGCATACAGCTTTAGTAGCAGGTTGGGCCGG
TTCAAT
> *Ephedra compacta* 4 (psbB). Mexico, Sierra Mojada, Coah (MOJ). Sierra_Moj_4
TCATTTCACACTATAAGTCAGCCCAGTCCAAAACTTCTTCTTTTGTTTCTTTTTTCTTTTTATTATCTTTGTTTTCCGGCTCTTTATCCT
CTCCTGTTTCGTTATCTTTTGCCAAAGTCATAATAGGTGCCTCATGCATTATATTATTCTTAGTTTTTTTTGATCCTAGAACCGGAAA
AGTACATGGGGTCTCATTCTTTCGTTTAGGCTTAGGTATTGTTGTCTCTTCAAAAAAGTGATTAAATTGAATCTCTTGAGGAGTTCT
ATCATCTTCTTTTCTTGACGGAGGGTTATCTTCACCAAAAAAACGAACTTTTGGGATACCAAGGGGCATTTTTTTTAGATCCTTTTTT
TTCTCTTTTTATTCTCCTTCTTCTCTTTCTTTTTGTTTTCCAAATTTTGTAAGTATTTTTTGTTTTGAATGGTACCAATCCTTAAATTTT
GTAAATTGAAACATAAAAGATAAAATATTTTTGCTTCTCCTACCGGTGTTTTTATTCAACATAGTTTTATAAAAGGAAGGATGATCC

AACCTAAAATTCAGTGTGTTTTTATAATGTAAGAAAGTTCAATCTTTTTTTTTTGAAAAAGAGGTGTTTAATGGGTTTACCTTGGTA
TCGTGTGCATACTGTTGTCTTGAATGATCCTGGCCGGTTAATTTCTGTGCATATAATGCATACAGCTTTAGTAGCAGGTTGGGCCGG
TTCAAT
> *Ephedra compacta* 5 (psbB). Mexico, Sierra Mojada, Coah (MOJ). Sierra_Moj_5
TCATTTCACACTATAAGTCAGCCCAGTCCAAAACTTCTTCTTTTGTTTCTTTTTTCTTTTTATTATCTTTGTTTTCCGGCTCTTTATCCT
CTCCTGTTTCGTTATCTTTTGCCAAAGTCATAATAGGTGCCTCATGCATTATATTATTCTTAGTTTTTTTTGATCCTAGAACCGGAAA
AGTACATGGGGTCTCATTCTTTCGTTTAGGCTTAGGTATTGTTGTCTCTTCAAAAAAGTGATTAAATTGAATCTCTTGAGGAGTTCT
ATCATCTTCTTTTCTTGACGGAGGGTTATCTTCACCAAAAAAACGAACTTTTGGGATACCAAGGGGCATTTTTTTTAGATCCTTTTTT
TTCTCTTTTTATTCTCCTTCTTCTCTTTCTTTTTGTTTTCCAAATTTTGTAAGTATTTTTTGTTTTGAATGGTACCAATCCTTAAATTTT
GTAAATTGAAACATAAAAGATAAAATATTTTTGCTTCTCCTACCGGTGTTTTTATTCAACATAGTTTTATAAAAGGAAGGATGATCC
AACCTAAAATTCAGTGTGTTTTTATAATGTAAGAAAGTTCAATCTTTTTTTTTTGAAAAAGAGGTGTTTAATGGGTTTACCTTGGTA
TCGTGTGCATACTGTTGTCTTGAATGATCCTGGCCGGTTAATTTCTGTGCATATAATGCATACAGCTTTAGTAGCAGGTTGGGCCGG
TTCAAT
> *Ephedra compacta* 1 (psbB). Mexico, Real de Catorce SLP (RC). Real_C_1
TCATTTCACACTATAAGTCAGCCCAGTCCAAAACTTCTTCTTTTGTTTCTTTTTTCTTTTTATTATCTTTGTTTTCCGGCTCTTTATCCT
CTCCTGTTTCGTTATCTTTTGCCAAAGTCATAATAGGTGCCTCATGCATTATATTATTCTTAGTTTTTTTTGATCCTAGAACCGGAAA
AGTACATGGGGTCTCATTCTTTCGTTTAGGCTTAGGTATTGTTGTCTCTTCAAAAAAGTGATTAAATTGAATCTCTTGAGGAGTTCT
ATCATCTTCTTTTCTTGACGGAGGGTTATCTTCACCAAAAAAACGAACTTTTGGGATACCAAGGGGCATTTTTTTTAGATCCTTTTTT
TTCTCTTTTTATTCTCCTTCTTCTCTTTCTTTTTGTTTTCCAAATTTTGTAAGTATTTTTTGTCTTGAATGGTACCAATCCTTCAATTTT
GTAAATTGAAACATAAAAGATAAAATATTTTTGCTTCTCCTACCGGTGTTTTTATTCAACATAGTTTTATAAAAGGAAGGATGATCC
AACCTAAAATTCAGTGTGTTTTTATAATGTAAGAAAGTTCAATCTTTTTTTTTTGAAAAAGAGGTGTTTAATGGGTTTACCTTGGTA
TCGTGTGCATACTGTTGTCTTGAATGATCCTGGCCGGTTAATTTCTGTGCATATAATGCATACAGCTTTAGTAGCAGGTTGGGCCGG
TTCAAT
> *Ephedra compacta* 2 (psbB). Mexico, Real de Catorce SLP (RC). Real_C_2
TCATTTCACACTATAAGTCAGCCCAGTCCAAAACTTCTTCTTTTGTTTCTTTTTTCTTTTTATTATCTTTGTTTTCCGGCTCTTTATCCT
CTCCTGTTTCGTTATCTTTTGCCAAAGTCATAATAGGTGCCTCATGCATTATATTATTCTTAGTTTTTTTTGATCCTAGAACCGGAAA
AGTACATGGGGTCTCATTCTTTCGTTTAGGCTTAGGTATTGTTGTCTCTTCAAAAAAGTGATTAAATTGAATCTCTTGAGGAGTTCT
ATCATCTTCTTTTCTTGACGGAGGGTTATCTTCACCAAAAAAACGAACTTTTGGGATACCAAGGGGCATTTTTTTTAGATCCTTTTTT
TTCTCTTTTTATTCTCCTTCTTCTCTTTCTTTTTGTTTTCCAAATTTTGTAAGTATTTTTTGTCTTGAATGGTACCAATCCTTCAATTTT
GTAAATTGAAACATAAAAGATAAAATATTTTTGCTTCTCCTACCGGTGTTTTTATTCAACATAGTTTTATAAAAGGAAGGATGATCC
AACCTAAAATTCAGTGTGTTTTTATAATGTAAGAAAGTTCAATCTTTTTTTTTTGAAAAAGAGGTGTTTAATGGGTTTACCTTGGTA
TCGTGTGCATACTGTTGTCTTGAATGATCCTGGCCGGTTAATTTCTGTGCATATAATGCATACAGCTTTAGTAGCAGGTTGGGCCGG
TTCAAT
> *Ephedra compacta* 1 (psbB). Mexico, Rocamontes, Dgo (ROCA). Rocamontes_1
TCATTTCACACTATAAGTCAGCCCAGTCCAAAACTTCTTCTTTTGTTTCTTTTTTCTTTTTATTATCTTTGTTTTCCGGCTCTTTATCCT
CTCCTGTTTCGTTATCTTTTGCCAAAGTCATAATAGGTGCCTCATGCATTATATTATTCTTAGTTTTTTTTGATCCTAGAACCGGAAA
AGTACATGGGGTCTCATTCTTTCGTTTAGGCTTAGGTATTGTTGTCTCTTCAAAAAAGTGATTAAATTGAATCTCTTGAGGAGTTCT
ATCATCTTCTTTTCTTGACGGAGGGTTATCTTCACCAAAAAAACGAACTTTTGGGATACCAAGGGGCATTTTTTTTAGATCCTTTTTT
TTCTCTTTTTATTCTCCTTCTTCTCTTTCTTTTTGTTTTCCAAATTTTGTAAGTATTTTTTGTTTTGAATGGTACCAATCCTTCAATTTT
GTAAATTGAAACATAAAAGATAAAATATTTTTGCTTCTCCTACCGGTGTTTTTATTCAACATAGTTTTATAAAAGGAAGGATGATCC
AACCTAAAATTCAGTGTGTTTTTATAATGTAAGAAAGTTCAATCTTTTTTTTTTGAAAAAGAGGTGTTTAATGGGTTTACCTTGGTA

TCGTGTGCATACTGTTGTCTTGAATGATCCTGGCCGGTTAATTTCTGTGCATATAATGCATACAGCTTTAGTAGCAGGTTGGGCCGG
TTCAAT

> *Ephedra compacta* 4 (psbB). Mexico, Rocamontes, Dgo (ROCA). Rocamontes_4
TCATTTCACACTATAAGTCAGCCCAGTCCAAAACTTCTTCTTTTGTTTCTTTTTTCTTTTTATTATCTTTGTTTTCCGGCTCTTTATCCT
CTCCTGTTTCGTTATCTTTTGCCAAAGTCATAATAGGTGCCTCATGCATTATATTATTCTTAGTTTTTTTTGATCCTAGAACCGGAAA
AGTACATGGGGTCTCATTCTTTCGTTTAGGCTTAGGTATTGTTGTCTCTTCAAAAAAGTGATTAAATTGAATCTCTTGAGGAGTTCT
ATCATCTTCTTTTCTTGACGGAGGGTTATCTTCACCAAAAAAACGAACTTTTGGGATACCAAGGGGCATTTTTTTTAGATCCTTTTTT
TTCTCTTTTTATTCTCCTTCTTCTCTTTCTTTTTGTTTTCCAAATTTTGTAAGTATTTTTTGTTTTGAATGGTACCAATCCTTCAATTTT
GTAAATTGAAACATAAAAGATAAAATATTTTTGCTTCTCCTACCGGTGTTTTTATTCAACATAGTTTTATAAAAGGAAGGATGATCC
AACCTAAAATTCAGTGTGTTTTTATAATGTAAGAAAGTTCAATCTTTTTTTTTTGAAAAAGAGGTGTTTAATGGGTTTACCTTGGTA
TCGTGTGCATACTGTTGTCTTGAATGATCCTGGCCGGTTAATTTCTGTGCATATAATGCATACAGCTTTAGTAGCAGGTTGGGCCGG
TTCAAT

> *Ephedra compacta* 5 (psbB). Mexico, Rocamontes, Dgo (ROCA). Rocamontes_5
TCATTTCACACTATAAGTCAGCCCAGTCCAAAACTTCTTCTTTTGTTTCTTTTTTCTTTTTATTATCTTTGTTTTCCGGCTCTTTATCCT
CTCCTGTTTCGTTATCTTTTGCCAAAGTCATAATAGGTGCCTCATGCATTATATTATTCTTAGTTTTTTTTGATCCTAGAACCGGAAA
AGTACATGGGGTCTCATTCTTTCGTTTAGGCTTAGGTATTGTTGTCTCTTCAAAAAAGTGATTAAATTGAATCTCTTGAGGAGTTCT
ATCATCTTCTTTTCTTGACGGAGGGTTATCTTCACCAAAAAAACGAACTTTTGGGATACCAAGGGGCATTTTTTTTAGATCCTTTTTT
TTCTCTTTTTATTCTCCTTCTTCTCTTTCTTTTTGTTTTCCAAATTTTGTAAGTATTTTTTGTTTTGAATGGTACCAATCCTTCAATTTT
GTAAATTGAAACATAAAAGATAAAATATTTTTGCTTCTCCTACCGGTGTTTTTATTCAACATAGTTTTATAAAAGGAAGGATGATCC
AACCTAAAATTCAGTGTGTTTTTATAATGTAAGAAAGTTCAATCTTTTTTTTTTGAAAAAGAGGTGTTTAATGGGTTTACCTTGGTA
TCGTGTGCATACTGTTGTCTTGAATGATCCTGGCCGGTTAATTTCTGTGCATATAATGCATACAGCTTTAGTAGCAGGTTGGGCCGG
TTCAAT

> *Ephedra compacta* 6 (psbB). Mexico, Rocamontes, Dgo (ROCA). Rocamontes_6
TCATTTCACACTATAAGTCAGCCCAGTCCAAAACTTCTTCTTTTGTTTCTTTTTTCTTTTTATTATCTTTGTTTTCCGGCTCTTTATCCT
CTCCTGTTTCGTTATCTTTTGCCAAAGTCATAATAGGTGCCTCATGCATTATATTATTCTTAGTTTTTTTTGATCCTAGAACCGGAAA
AGTACATGGGGTCTCATTCTTTCGTTTAGGCTTAGGTATTGTTGTCTCTTCAAAAAAGTGATTAAATTGAATCTCTTGAGGAGTTCT
ATCATCTTCTTTTCTTGACGGAGGGTTATCTTCACCAAAAAAACGAACTTTTGGGATACCAAGGGGCATTTTTTTTAGATCCTTTTTT
TTCTCTTTTTATTCTCCTTCTTCTCTTTCTTTTTGTTTTCCAAATTTTGTAAGTATTTTTTGTTTTGAATGGTACCAATCCTTCAATTTT
GTAAATTGAAACATAAAAGATAAAATATTTTTGCTTCTCCTACCGGTGTTTTTATTCAACATAGTTTTATAAAAGGAAGGATGATCC
AACCTAAAATTCAGTGTGTTTTTATAATGTAAGAAAGTTCAATCTTTTTTTTTTGAAAAAGAGGTGTTTAATGGGTTTACCTTGGTA
TCGTGTGCATACTGTTGTCTTGAATGATCCTGGCCGGTTAATTTCTGTGCATATAATGCATACAGCTTTAGTAGCAGGTTGGGCCGG
TTCAAT

> *Ephedra compacta* 7 (psbB). Mexico, Rocamontes, Dgo (ROCA). Rocamontes_7
TCATTTCACACTATAAGTCAGCCCAGTCCAAAACTTCTTCTTTTGTTTCTTTTTTCTTTTTATTATCTTTGTTTTCCGGCTCTTTATCCT
CTCCTGTTTCGTTATCTTTTGCCAAAGTCATAATAGGTGCCTCATGCATTATATTATTCTTAGTTTTTTTTGATCCTAGAACCGGAAA
AGTACATGGGGTCTCATTCTTTCGTTTAGGCTTAGGTATTGTTGTCTCTTCAAAAAAGTGATTAAATTGAATCTCTTGAGGAGTTCT
ATCATCTTCTTTTCTTGACGGAGGGTTATCTTCACCAAAAAAACGAACTTTTGGGATACCAAGGGGCATTTTTTTTAGATCCTTTTTT
TTCTCTTTTTATTCTCCTTCTTCTCTTTCTTTTTGTTTTCCAAATTTTGTAAGTATTTTTTGTTTTGAATGGTACCAATCCTTCAATTTT
GTAAATTGAAACATAAAAGATAAAATATTTTTGCTTCTCCTACCGGTGTTTTTATTCAACATAGTTTTATAAAAGGAAGGATGATCC
AACCTAAAATTCAGTGTGTTTTTATAATGTAAGAAAGTTCAATCTTTTTTTTTTGAAAAAGAGGTGTTTAATGGGTTTACCTTGGTA
TCGTGTGCATACTGTTGTCTTGAATGATCCTGGCCGGTTAATTTCTGTGCATATAATGCATACAGCTTTAGTAGCAGGTTGGGCCGG
TTCAAT

> ***Ephedra compacta* 1. (psbB). Mexico, Cuatro Cienegas, Coah (CC).** Cuatro_Cie_1
TCATTTCACACTATAAGTCAGCCCAGTCCAAAACTTCTTCTTTTGTTTCTTTTTTCTTTTTATTATCTTTGTTTTCCGGCTCTTTATCCT
CTCCTGTTTCGTTATCTTTTGCCAAAGTCATAATAGGTGCCTCATGCATTATATTATTCTTAGTTTTTTTTGATCCTAGAACCGGAAA
AGTACATGGGGTCTCATTCTTTCGTTTAGGCTTAGGTATTGTTGTCTCTTCAAAAAAGTGATTAAATTGAATCTCTTGAGGAGTTCT
ATCATCTTCTTTTCTTGACGGAGGGTTATCTTCACCAAAAAAACGAACTTTTGGGATACCAAGGGGCATTTTTTTTAGATCCTTTTTT
TTCTCTTTTTATTCTCCTTCTTCTCTTTCTTTTTGTTTTCCAAATTTTGTAAGTATTTTTTGTTTTGAATGGTACCAATCCTTAAATTTT
GTAAATTGAAACATAAAAGATAAAATATTTTTGCTTCTCCTACCGGTGTTTTTATTCAACATAGTTTTATAAAAGGAAGGATGATCC
AACCTAAAATTCAGTGTGTTTTTATAATGTAAGAAAGTTCAATCTTTTTTTTTGAAAAAGAGGTGTTTAATGGGTTTACCTTGGTAT
CGTGTGCATACTGTTGTCTTGAATGATCCTGGCCGGTTAATTTCTGTGCATATAATGCATACAGCTTTAGTAGCAGGTTGGGCCGGT
TCAAC

> ***Ephedra compacta* 2. (psbB). Mexico, Cuatro Cienegas, Coah (CC).** Cuatro_Cie_2
TCATTTCACACTATAAGTCAGCCCAGTCCAAAACTTCTTCTTTTGTTTCTTTTTTCTTTTTATTATCTTTGTTTTCCGGCTCTTTATCCT
CTCCTGTTTCGTTATCTTTTGCCAAAGTCATAATAGGTGCCTCATGCATTATATTATTCTTAGTTTTTTTTGATCCTAGAACCGGAAA
AGTACATGGGGTCTCATTCTTTCGTTTAGGCTTAGGTATTGTTGTCTCTTCAAAAAAGTGATTAAATTGAATCTCTTGAGGAGTTCT
ATCATCTTCTTTTCTTGACGGAGGGTTATCTTCACCAAAAAAACGAACTTTTGGGATACCAAGGGGCATTTTTTTTAGATCCTTTTTT
TTCTCTTTTTATTCTCCTTCTTCTCTTTCTTTTTGTTTTCCAAATTTTGTAAGTATTTTTTGTTTTGAATGGTACCAATCCTTAAATTTTT
TAAATTGAAACATAAAAGATAAAATATTTTTGCTTCTCCTACCGGTGTTTTTATTCAACATAGTTTTATAAAAGGAAGGATGATCCA
ACCTAAAATTCAGTGTGTTTTTATAATGTAAGAAAGTTCAATCTTTTTTTTTGAAAAAGAGGTGTTTAATGGGTTTACCTTGGTATC
GTGTGCATACTGTTGTCTTGAATGATCCTGGCCGGTTAATTTCTGTGCATATAATGCATACAGCTTTAGTAGCAGGTTGGGCCGGTT
CAAC

> ***Ephedra compacta* 4. (psbB). Mexico, Cuatro Cienegas, Coah (CC).** Cuatro_Cie_4
TCATTTCACACTATAAGTCAGCCCAGTCCAAAACTTCTTCTTTTGTTTCTTTTTTCTTTTTATTATCTTTGTTTTCCGGCTCTTTATCCT
CTCCTGTTTCGTTATCTTTTGCCAAAGTCATAATAGGTGCCTCATGCATTATATTATTCTTAGTTTTTTTTGATCCTAGAACCGGAAA
AGTACATGGGGTCTCATTCTTTCGTTTAGGCTTAGGTATTGTTGTCTCTTCAAAAAAGTGATTAAATTGAATCTCTTGAGGAGTTCT
ATCATCTTCTTTTCTTGACGGAGGGTTATCTTCACCAAAAAAACGAACTTTTGGGATACCAAGGGGCATTTTTTTTAGATCCTTTTTT
TTCTCTTTTTATTCTCCTTCTTCTCTTTCTTTTTGTTTTCCAAATTTTGTAAGTATTTTTTGTTTTGAATGGTACCAATCCTTAAATTTT
GTAAATTGAAACATAAAAGATAAAATATTTTTGCTTCTCCTACCGGTGTTTTTATTCAACATAGTTTTATAAAAGGAAGGATGATCC
AACCTAAAATTCAGTGTGTTTTTATAATGTAAGAAAGTTCAATCTTTTTTTTTGAAAAAGAGGTGTTTAATGGGTTTACCTTGGTAT
CGTGTGCATACTGTTGTCTTGAATGATCCTGGCCGGTTAATTTCTGTGCATATAATGCATACAGCTTTAGTAGCAGGTTGGGCCGGT
TCAAC

> ***Ephedra compacta* 1. (psbB). Mexico, Parras, Coah (PARR).** Parras_1
TCATTTCACACTATAAGTCAGCCCAGTCCAAAACTTCTTCTTTTGTTTCTTTTTTCTTTTTATTATCTTTGTTTTCCGGCTCTTTATCCT
CTCCTGTTTCGTTATCTTTTGCCAAAGTCATAATAGGTGCCTCATGCATTATATTATTCTTAGTTTTTTTTGATCCTAGAACCGGAAA
AGTACATGGGGTCTCATTCTTTCGTTTAGGCTTAGGTATTGTTGTCTCTTCAAAAAAGTGATTAAATTGAATCTCTTGAGGAGTTCT
ATCATCTTCTTTTCTTGACGGAGGGTTATCTTCACCAAAAAAACGAACTTTTGGGATACCAAGGGGCATTTTTTTTAGATCCTTTTTT
TTCTCTTTTTATTCTCCTTCTTCTCTTTCTTTTTGTTTTCCAAATTTTGTAAGTATTTTTTGTTTTGAATGGTACCAATCCTTAAATTTTT
TAAATTGAAACATAAAAGATAAAATATTTTTGCTTCTCCTACCGGTGTTTTTATTCAACATAGTTTTATAAAAGGAAGGATGATCCA
ACCTAAAATTCAGTGTGTTTTTATAATGTAAGAAAGTTCAATCTTTTTTTTTGAAAAAGAGGTGTTTAATGGGTTTACCTTGGTATC
GTGTGCATACTGTTGTCTTGAATGATCCTGGCCGGTTAATTTCTGTGCATATAATGCATACAGCTTTAGTAGCAGGTTGGGCCGGTT
CAAC

> ***Ephedra compacta* 3. (psbB). Mexico, Parras, Coah (PARR).** Parras_3

TCATTTCACACTATAAGTCAGCCCAGTCCAAAACTTCTTCTTTTGTTTCTTTTTTCTTTTTATTATCTTTGTTTTCCGGCTCTTTATCCT
CTCCTGTTTCGTTATCTTTTGCCAAAGTCATAATAGGTGCCTCATGCATTATATTATTCTTAGTTTTTTTTGATCCTAGAACCGGAAA
AGTACATGGGGTCTCATTCTTTCGTTTAGGCTTAGGTATTGTTGTCTCTTCAAAAAAGTGATTAAATTGAATCTCTTGAGGAGTTCT
ATCATCTTCTTTTCTTGACGGAGGGTTATCTTCACCAAAAAAACGAACTTTTGGGATACCAAGGGGCATTTTTTTTAGATCCTTTTTT
TTCTCTTTTTATTCTCCTTCTTCTCTTTCTTTTTGTTTTCCAAATTTTGTAAGTATTTTTTGTTTTGAATGGTACCAATCCTTAAATTTTT
TAAATTGAAACATAAAAGATAAAATATTTTTGCTTCTCCTACCGGTGTTTTTATTCAACATAGTTTTATAAAAGGAAGGATGATCCA
ACCTAAAATTCAGTGTGTTTTTATAATGTAAGAAAGTTCAATCTTTTTTTTTGAAAAAGAGGTGTTTAATGGGTTTACCTTGGTATC
GTGTGCATACTGTTGTCTTGAATGATCCTGGCCGGTTAATTTCTGTGCATATAATGCATACAGCTTTAGTAGCAGGTTGGGCCGGTT
CAAC

> *Ephedra compacta* 1. (psbB). Mexico, Parral, Chih (PARRAL). Parral_1
TCATTTCACACTATAAGTCAGCCCAGTCCAAAACTTCTTCTTTTGTTTCTTTTTTCTTTTTATTATCTTTGTTTTCCGGCTCTTTATCCT
CTCCTGTTTCGTTATCTTTTGCCAAAGTCATAATAGGTGCCTCATGCATTATATTATTCTTAGTTTTTTTTGATCCTAGAACCGGAAA
AGTACATGGGGTCTCATTCTTTCGTTTAGGCTTAGGTATTGTTGTCTCTTCAAAAAAGTGATTAAATTGAATCTCTTGAGGAGTTCT
ATCATCTTCTTTTCTTGACGGAGGGTTATCTTCACCAAAAAAACGAACTTTTGGGATACCAAGGGGCATTTTTTTTAGATCCTTTTTT
TTCTCTTTTTATTCTCCTTCTTCTCTTTCTTTTTGTTTTCCAAATTTTGTAAGTATTTTTTGTTTTGAATGGTACCAATCCTTCAATTTT
GTAAATTGAAACATAAAAGATAAAATATTTTTGCTTCTCCTACCGGTGTTTTTATTCAACATAGTTTTATAAAAGGAAGGATGATCC
AACCTAAAATTCAGTGTGTTTTTATAATGTAAGAAAGTTCAATCTTTTTTTTTGAAAAAGAGGTGTTTAATGGGTTTACCTTGGTAT
CGTGTGCATACTGTTGTCTTGAATGATCCTGGCCGGTTAATTTCTGTGCATATAATGCATACAGCTTTAGTAGCAGGTTGGGCCGGT
TCAAC

> *Ephedra compacta* 2. (psbB). Mexico, Parral, Chih (PARRAL). Parral_2
TCATTTCACACTATAAGTCAGCCCAGTCCAAAACTTCTTCTTTTGTTTCTTTTTTCTTTTTATTATCTTTGTTTTCCGGCTCTTTATCCT
CTCCTGTTTCGTTATCTTTTGCCAAAGTCATAATAGGTGCCTCATGCATTATATTATTCTTAGTTTTTTTTGATCCTAGAACCGGAAA
AGTACATGGGGTCTCATTCTTTCGTTTAGGCTTAGGTATTGTTGTCTCTTCAAAAAAGTGATTAAATTGAATCTCTTGAGGAGTTCT
ATCATCTTCTTTTCTTGACGGAGGGTTATCTTCACCAAAAAAACGAACTTTTGGGATACCAAGGGGCATTTTTTTTAGATCCTTTTTT
TTCTCTTTTTATTCTCCTTCTTCTCTTTCTTTTTGTTTTCCAAATTTTGTAAGTATTTTTTGTTTTGAATGGTACCAATCCTTCAATTTT
GTAAATTGAAACATAAAAGATAAAATATTTTTGCTTCTCCTACCGGTGTTTTTATTCAACATAGTTTTATAAAAGGAAGGATGATCC
AACCTAAAATTCAGTGTGTTTTTATAATGTAAGAAAGTTCAATCTTTTTTTTTGAAAAAGAGGTGTTTAATGGGTTTACCTTGGTAT
CGTGTGCATACTGTTGTCTTGAATGATCCTGGCCGGTTAATTTCTGTGCATATAATGCATACAGCTTTAGTAGCAGGTTGGGCCGGT
TCAAC

> *Ephedra compacta* 1. (psbB). México, Galeana, NL (GAL). Galeana_1
TCATTTCACACTATAAGTCAGCCCAGTCCAAAACTTCTTCTTTTGTTTCTTTTTTCTTTTTATTATCTTTGTTTTCCGGCTCTTTATCCT
CTCCTGTTTCGTTATCTTTTGCCAAAGTCATAATAGGTGCCTCATGCATTATATTATTCTTAGTTTTTTTTGATCCTAGAACCGGAAA
AGTACATGGGGTCTCATTCTTTCGTTTAGGCTTAGGTATTGTTGTCTCTTCAAAAAAGTGATTAAATTGAATCTCTTGAGGAGTTCT
ATCATCTTCTTTTCTTGACGGAGGGTTATCTTCACCAAAAAAACGAACTTTTGGGATACCAAGGGGCATTTTTTTTAGATCCTTTTTT
TTCTCTTTTTATTCTCCTTCTTCTCTTTCTTTTTGTTTTCCAAATTTTGTAAGTATTTTTTGTTTTGAATGGTACCAATCCTTCAATTTT
GTAAATTGAAACATAAAAGATAAAATATTTTTGCTTCTCCTACCGGTGTTTTTATTCAACATAGTTTTATAAAAGGAAGGATGATCC
AACCTAAAATTCAGTGTGTTTTTATAATGTAAGAAAGTTCAATCTTTTTTTTTTGAAAAAGAGGTGTTTAATGGGTTTACCTTGGTA
TCGTGTGCATACTGTTGTCTTGAATGATCCTGGCCGGTTAATTTCTGTGCATATAATGCATACAGCTTTAGTAGCAGGTTGGGCCGG
TTCAAT

> *Ephedra compacta* 3. (psbB). México, Galeana, NL (GAL). Galeana_3
TCATTTCACACTATAAGTCAGCCCAGTCCAAAACTTCTTCTTTTGTTTCTTTTTTCTTTTTATTATCTTTGTTTTCCGGCTCTTTATCCT
CTCCTGTTTCGTTATCTTTTGCCAAAGTCATAATAGGTGCCTCATGCATTATATTATTCTTAGTTTTTTTTGATCCTAGAACCGGAAA

AGTACATGGGGTCTCATTCTTTCGTTTAGGCTTAGGTATTGTTGTCTCTTCAAAAAAGTGATTAAATTGAATCTCTTGAGGAGTTCT
ATCATCTTCTTTTCTTGACGGAGGGTTATCTTCACCAAAAAAACGAACTTTTGGGATACCAAGGGGCATTTTTTTTAGATCCTTTTTT
TTCTCTTTTTATTCTCCTTCTTCTCTTTCTTTTTGTTTTCCAAATTTTGTAAGTATTTTTTGTTTTGAATGGTACCAATCCTTCAATTTT
GTAAATTGAAACATAAAAGATAAAATATTTTTGCTTCTCCTACCGGTGTTTTTATTCAACATAGTTTTATAAAAGGAAGGATGATCC
AACCTAAAATTCAGTGTGTTTTTATAATGTAAGAAAGTTCAATCTTTTTTTTTTGAAAAAGAGGTGTTTAATGGGTTTACCTTGGTA
TCGTGTGCATACTGTTGTCTTGAATGATCCTGGCCGGTTAATTTCTGTGCATATAATGCATACAGCTTTAGTAGCAGGTTGGGCCGG
TTCAAT

> *Ephedra compacta* 4. (psbB). México, Galeana, NL (GAL). Galeana_4
TCATTTCACACTATAAGTCAGCCCAGTCCAAAACTTCTTCTTTTGTTTCTTTTTTCTTTTTATTATCTTTGTTTTCCGGCTCTTTATCCT
CTCCTGTTTCGTTATCTTTTGCCAAAGTCATAATAGGTGCCTCATGCATTATATTATTCTTAGTTTTTTTTGATCCTAGAACCGGAAA
AGTACATGGGGTCTCATTCTTTCGTTTAGGCTTAGGTATTGTTGTCTCTTCAAAAAAGTGATTAAATTGAATCTCTTGAGGAGTTCT
ATCATCTTCTTTTCTTGACGGAGGGTTATCTTCACCAAAAAAACGAACTTTTGGGATACCAAGGGGCATTTTTTTTAGATCCTTTTTT
TTCTCTTTTTATTCTCCTTCTTCTCTTTCTTTTTGTTTTCCAAATTTTGTAAGTATTTTTTGTTTTGAATGGTACCAATCCTTCAATTTT
GTAAATTGAAACATAAAAGATAAAATATTTTTGCTTCTCCTACCGGTGTTTTTATTCAACATAGTTTTATAAAAGGAAGGATGATCC
AACCTAAAATTCAGTGTGTTTTTATAATGTAAGAAAGTTCAATCTTTTTTTTTTGAAAAAGAGGTGTTTAATGGGTTTACCTTGGTA
TCGTGTGCATACTGTTGTCTTGAATGATCCTGGCCGGTTAATTTCTGTGCATATAATGCATACAGCTTTAGTAGCAGGTTGGGCCGG
TTCAAT

> *Ephedra compacta* 5. (psbB). México, Galeana, NL (GAL). Galeana_5
TCATTTCACACTATAAGTCAGCCCAGTCCAAAACTTCTTCTTTTGTTTCTTTTTTCTTTTTATTATCTTTGTTTTCCGGCTCTTTATCCT
CTCCTGTTTCGTTATCTTTTGCCAAAGTCATAATAGGTGCCTCATGCATTATATTATTCTTAGTTTTTTTTGATCCTAGAACCGGAAA
AGTACATGGGGTCTCATTCTTTCGTTTAGGCTTAGGTATTGTTGTCTCTTCAAAAAAGTGATTAAATTGAATCTCTTGAGGAGTTCT
ATCATCTTCTTTTCTTGACGGAGGGTTATCTTCACCAAAAAAACGAACTTTTGGGATACCAAGGGGCATTTTTTTTAGATCCTTTTTT
TTCTCTTTTTATTCTCCTTCTTCTCTTTCTTTTTGTTTTCCAAATTTTGTAAGTATTTTTTGTTTTGAATGGTACCAATCCTTCAATTTT
GTAAATTGAAACATAAAAGATAAAATATTTTTGCTTCTCCTACCGGTGTTTTTATTCAACATAGTTTTATAAAAGGAAGGATGATCC
AACCTAAAATTCAGTGTGTTTTTATAATGTAAGAAAGTTCAATCTTTTTTTTTTGAAAAAGAGGTGTTTAATGGGTTTACCTTGGTA
TCGTGTGCATACTGTTGTCTTGAATGATCCTGGCCGGTTAATTTCTGTGCATATAATGCATACAGCTTTAGTAGCAGGTTGGGCCGG
TTCAAT

> *Ephedra compacta* 6. (psbB). México, Galeana, NL (GAL). Galeana_6
TCATTTCACACTATAAGTCAGCCCAGTCCAAAACTTCTTCTTTTGTTTCTTTTTTCTTTTTATTATCTTTGTTTTCCGGCTCTTTATCCT
CTCCTGTTTCGTTATCTTTTGCCAAAGTCATAATAGGTGCCTCATGCATTATATTATTCTTAGTTTTTTTTGATCCTAGAACCGGAAA
AGTACATGGGGTCTCATTCTTTCGTTTAGGCTTAGGTATTGTTGTCTCTTCAAAAAAGTGATTAAATTGAATCTCTTGAGGAGTTCT
ATCATCTTCTTTTCTTGACGGAGGGTTATCTTCACCAAAAAAACGAACTTTTGGGATACCAAGGGGCATTTTTTTTAGATCCTTTTTT
TTCTCTTTTTATTCTCCTTCTTCTCTTTCTTTTTGTTTTCCAAATTTTGTAAGTATTTTTTGTTTTGAATGGTACCAATCCTTCAATTTT
GTAAATTGAAACATAAAAGATAAAATATTTTTGCTTCTCCTACCGGTGTTTTTATTCAACATAGTTTTATAAAAGGAAGGATGATCC
AACCTAAAATTCAGTGTGTTTTTATAATGTAAGAAAGTTCAATCTTTTTTTTTTGAAAAAGAGGTGTTTAATGGGTTTACCTTGGTA
TCGTGTGCATACTGTTGTCTTGAATGATCCTGGCCGGTTAATTTCTGTGCATATAATGCATACAGCTTTAGTAGCAGGTTGGGCCGG
TTCAAT

>*Leucophyllum laevigatum* 1. (trnH-psbA) Mexico, San Juan del Rio, Dgo (ATO). L_laevigatumAto1
CGTAATGCTCATAACTTCCCTCTAGATCTAGCTGCTATTGAAGCTCCAACAAATGGATAAGACTTGGTCTTAGTGTATAGGGGTTTT
TGAAAATAGAATATATAAATATAAGGAGCAATAAACTCTTTCTTGTTCTATCAAGAGGGGTTATTGCTCCTTTATTTTCTTTTCAAT
GAGTAGTATTTTGTTAAAAGGGTTTTAAGGGATTGATTAATGATGGAGTATTTTTCTTTCGTTCTGTATGAATTTTTTTCTTAATCTT
TTTATTTTAAAGATAAGAATATTTCATAAAAAAGAAAGAAGATAACTGAAATGATCTAAAGTCAACCTTTTGTCTTACAATCAATT

TGTAAAAAATCAAAATTGAAACATCGTATAATTTTAAATAATATAGGGGCGGATGTAGCCAAGTGGATCAAGGCAGTGGATTGTG
AATC
>*Leucophyllum laevigatum* 5. (trnH-psbA) Mexico, San Juan del Rio, Dgo (ATO). L_laevigatumAto5
CGTAATGCTCATAACTTCCCTCTAGATCTAGCTGCTATTGAAGCTCCAACAAATGGATAAGACTTGGTCTTAGTGTATAGGGGTTTT
TGAAAATAGAATATATAAATATAAGGAGCAATAAACTCTTTCTTGTTCTATCAAGAGGGGTTATTGCTCCTTTATTTTCTTTTCAAT
GAGTAGTATTTTGTTAAAAGGGTTTTAAGGGATTGATTAATGATGGAGTATTTTTCTTTCGTTCTGTATGAATTTTTTTCTTAATCTT
TTTATTTTAAAGATAAGAATATTTCATAAAAAAGAAAGAAGATAACTGAAATGATCTAAAGTCAACCTTTTGTCTTACAATCAATT
TGTAAAAAATCAAAATTGAAACATCGTATAATTTTAAATAATATAGGGGCGGATGTAGCCAAGTGGATCAAGGCAGTGGATTGTG
AATC
>*Leucophyllum laevigatum* 9. (trnH-psbA) Mexico, San Juan del Rio, Dgo (ATO). L_laevigatumAto9
CGTAATGCTCATAACTTCCCTCTAGATCTAGCTGCTATTGAAGCTCCAACAAATGGATAAGACTTGGTCTTAGTGTATAGGGGTTTT
TGAAAATAGAATATATAAATATAAGGAGCAATAAACTCTTTCTTGTTCTATCAAGAGGGGTTATTGCTCCTTTATTTTCTTTTCAAT
GAGTAGTATTTTGTTAAAAGGGTTTTAAGGGATTGATTAATGATGGAGTATTTTTCTTTCGTTCTGTATGAATTTTTTTCTTAATCTT
TTTATTTTAAAGATAAGAATATTTCATAAAAAAGAAAGAAGATAACTGAAATGATCTAAAGTCAACCTTTTGTCTTACAATCAATT
TGTAAAAAATCAAAATTGAAACATCGTATAATTTTAAATAATATAGGGGCGGATGTAGCCAAGTGGATCAAGGCAGTGGATTGTG
AATC
>*Leucophyllum laevigatum* 13. (trnH-psbA) Mexico, San Juan del Rio, Dgo (ATO). L_laevigatumAto13
CGTAATGCTCATAACTTCCCTCTAGATCTAGCTGCTATTGAAGCTCCAACAAATGGATAAGACTTGGTCTTAGTGTATAGGGGTTTT
TGAAAATAGAATATATAAATATAAGGAGCAATAAACTCTTTCTTGTTCTATCAAGAGGGGTTATTGCTCCTTTATTTTCTTTTCAAT
GAGTAGTATTTTGTTAAAAGGGTTTTAAGGGATTGATTAATGATGGAGTATTTTTCTTTCGTTCTGTATGAATTTTTTTCTTAATCTT
TTTATTTTAAAGATAAGAATATTTCATAAAAAAGAAAGAAGATAACTGAAATGATCTAAAGTCAACCTTTTGTCTTACAATCAATT
TGTAAAAAATCAAAATTGAAACATCGTATAATTTTAAATAATATAGGGGCGGATGTAGCCAAGTGGATCAAGGCAGTGGATTGTG
AATC
>*Leucophyllum laevigatum* 19. (trnH-psbA) Mexico, San Juan del Rio, Dgo (ATO). L_laevigatumAto19
CGTAATGCTCATAACTTCCCTCTAGATCTAGCTGCTATTGAAGCTCCAACAAATGGATAAGACTTGGTCTTAGTGTATAGGGGTTTT
TGAAAATAGAATATATAAATATAAGGAGCAATAAACTCTTTCTTGTTCTATCAAGAGGGGTTATTGCTCCTTTATTTTCTTTTCAAT
GAGTAGTATTTTGTTAAAAGGGTTTTAAGGGATTGATTAATGATGGAGTATTTTTCTTTCGTTCTGTATGAATTTTTTTCTTAATCTT
TTTATTTTAAAGATAAGAATATTTCATAAAAAAGAAAGAAGATAACTGAAATGATCTAAAGTCAACCTTTTGTCTTACAATCAATT
TGTAAAAAATCAAAATTGAAACATCGTATAATTTTAAATAATATAGGGGCGGATGTAGCCAAGTGGATCAAGGCAGTGGATTGTG
AATC
>*Leucophyllum laevigatum* 1. (trnH-psbA) Mexico, Rodeo, Dgo (ROD). L_laevigatumRod1
CGTAATGCTCATAACTTCCCTCTAGATCTAGCTGCTATTGAAGCTCCAACAAATGGATAAGACTTGGTCTTAGTGTATAGGGGTTTT
TGAAAATAGAATATATAAATATAAGGAGCAATAAACTCTTTCTTGTTCTATCAAGAGGGGTTATTGCTCCTTTATTTTCTTTTCAAT
GAGTAGTATTTTATTAAAAGGGTTTTAAGGGATTGATTAATGATGGAGTATTTTTCTTTCGTTCTGTATGAATTTTTTTCTTAATCTT
TTTATTTTAAAGATAAGAATATTTCATAAAAAAGAAAGAAGATAACTGAAATGATCTAAAGTCAACCTTTTGTCTTACAATCAATT
TGTAAAAAATCAAAATTGAAACATCGTATAATTTTAAATAATATAGGGGCGGATGTAGCCAAGTGGATCAAGGCAGTGGATTGTG
AATC
>*Leucophyllum laevigatum* 4. (trnH-psbA) Mexico, Rodeo, Dgo (ROD). L_laevigatumRod4
CGTAATGCTCATAACTTCCCTCTAGATCTAGCTGCTATTGAAGCTCCAACAAATGGATAAGACTTGGTCTTAGTGTATAGGGGTTTT
TGAAAATAGAATATATAAATATAAGGAGCAATAAACTCTTTCTTGTTCTATCAAGAGGGGTTATTGCTCCTTTATTTTCTTTTCAAT
GAGTAGTATTTTATTAAAAGGGTTTTAAGGGATTGATTAATGATGGAGTATTTTTCTTTCGTTCTGTATGAATTTTTTTCTTAATCTT
TTTATTTTAAAGATAAGAATATTTCATAAAAAAGAAAGAAGATAACTGAAATGATCTAAAGTCAACCTTTTGTCTTACAATCAATT

TGTAAAAAATCAAAATTGAAACATCGTATAATTTTAAATAATATAGGGGCGGATGTAGCCAAGTGGATCAAGGCAGTGGATTGTG
AATC
>*Leucophyllum laevigatum* 9. (trnH-psbA) Mexico, Rodeo, Dgo (ROD). L_laevigatumRod9
CGTAATGCTCATAACTTCCCTCTAGATCTAGCTGCTATTGAAGCTCCAACAAATGGATAAGACTTGGTCTTAGTGTATAGGGGTTTT
TGAAAATAGAATATATAAATATAAGGAGCAATAAACTCTTTCTTGTTCTATCAAGAGGGGTTATTGCTCCTTTATTTTCTTTTCAAT
GAGTAGTATTTTATTAAAAGGGTTTTAAGGGATTGATTAATGATGGAGTATTTTTCTTTCGTTCTGTATGAATTTTTTTCTTAATCTT
TTTATTTTAAAGATAAGAATATTTCATAAAAAAGAAAGAAGATAACTGAAATGATCTAAAGTCAACCTTTTGTCTTACAATCAATT
TGTAAAAAATCAAAATTGAAACATCGTATAATTTTAAATAATATAGGGGCGGATGTAGCCAAGTGGATCAAGGCAGTGGATTGTG
AATC
>*Leucophyllum laevigatum* 13. (trnH-psbA) Mexico, Rodeo, Dgo (ROD). L_laevigatumRod13
CGTAATGCTCATAACTTCCCTCTAGATCTAGCTGCTATTGAAGCTCCAACAAATGGATAAGACTTGGTCTTAGTGTATAGGGGTTTT
TGAAAATAGAATATATAAATATAAGGAGCAATAAACTCTTTCTTGTTCTATCAAGAGGGGTTATTGCTCCTTTATTTTCTTTTCAAT
GAGTAGTATTTTATTAAAAGGGTTTTAAGGGATTGATTAATGATGGAGTATTTTTCTTTCGTTCTGTATGAATTTTTTTCTTAATCTT
TTTATTTTAAAGATAAGAATATTTCATAAAAAAGAAAGAAGATAACTGAAATGATCTAAAGTCAACCTTTTGTCTTACAATCAATT
TGTAAAAAATCAAAATTGAAACATCGTATAATTTTAAATAATATAGGGGCGGATGTAGCCAAGTGGATCAAGGCAGTGGATTGTG
AATC
>*Leucophyllum laevigatum* 17. (trnH-psbA) Mexico, Rodeo, Dgo (ROD). L_laevigatumRod17
CGTAATGCTCATAACTTCCCTCTAGATCTAGCTGCTATTGAAGCTCCAACAAATGGATAAGACTTGGTCTTAGTGTATAGGGGTTTT
TGAAAATAGAATATATAAATATAAGGAGCAATAAACTCTTTCTTGTTCTATCAAGAGGGGTTATTGCTCCTTTATTTTCTTTTCAAT
GAGTAGTATTTTGTTAAAAGGGTTTTAAGGGATTGATTAATGATGGAGTATTTTTCTTTCGTTCTGTATGAATTTTTTTCTTAATCTT
TTTATTTTAAAGATAAGAATATTTCATAAAAAAGAAAGAAGATAACTGAAATGATCTAAAGTCAACCTTTTGTCTTACAATCAATT
TGTAAAAAATCAAAATTGAAACATCGTATAATTTTAAATAATATAGGGGCGGATGTAGCCAAGTGGATCAAGGCAGTGGATTGTG
AATC
>*Leucophyllum laevigatum* 1. (trnH-psbA) Mexico, La Zarca, Dgo (ZAR). L_laevigatumZar1
CGTAATGCTCATAACTTCCCTCTAGATCTAGCTGCTATTGAAGCTCCAACAAATGGATAAGACTTGGTCTTAGTGTATAGGGGTTTT
TGAAAATAGAATATATAAATATAAGGAGCAATAAACTCTTTCTTGTTCTATCAAGAGGGGTTATTGCTCCTTTATTTTCTTTTCAAT
GAGTAGTATTTTGTTAAAAGGGTTTTAAGGGATTGATTAATGATGGAGTATTTTTCTTTCGTTCTGTATGAATTTTTTTCTTAATCTT
TTTATTTTAAAGATAAGAATATTTCATAAAAAAGAAAGAAGATAACTGAAATGATCTAAAGTCAACCTTTTGTCTTACAATCAATT
TGTAAAAAATCAAAATTGAAACATCGTATAATTTTAAATAATATAGGGGCGGATGTAGCCAAGTGGATCAAGGCAGTGGATTGTG
AATC
>*Leucophyllum laevigatum* 2. (trnH-psbA) Mexico, La Zarca, Dgo (ZAR). L_laevigatumZar2
CGTAATGCTCATAACTTCCCTCTAGATCTAGCTGCTATTGAAGCTCCAACAAATGGATAAGACTTGGTCTTAGTGTATAGGGGTTTT
TGAAAATAGAATATATAAATATAAGGAGCAATAAACTCTTTCTTGTTCTATCAAGAGGGGTTATTGCTCCTTTATTTTCTTTTCAAT
GAGTAGTATTTTGTTAAAAGGGTTTTAAGGGATTGATTAATGATGGAGTATTTTTCTTTCGTTCTGTATGAATTTTTTTCTTAATCTT
TTTATTTTAAAGATAAGAATATTTCATAAAAAAGAAAGAAGATAACTGAAATGATCTAAAGTCAACCTTTTGTCTTACAATCAATT
TGTAAAAAATCAAAATTGAAACATCGTATAATTTTAAATAATATAGGGGCGGATGTAGCCAAGTGGATCAAGGCAGTGGATTGTG
AATC
>*Leucophyllum laevigatum* 3. (trnH-psbA) Mexico, La Zarca, Dgo (ZAR). L_laevigatumZar3
CGTAATGCTCATAACTTCCCTCTAGATCTAGCTGCTATTGAAGCTCCAACAAATGGATAAGACTTGGTCTTAGTGTATAGGGGTTTT
TGAAAATAGAATATATAAATATAAGGAGCAATAAACTCTTTCTTGTTCTATCAAGAGGGGTTATTGCTCCTTTATTTTCTTTTCAAT
GAGTAGTATTTTGTTAAAAGGGTTTTAAGGGATTGATTAATGATGGAGTATTTTTCTTTCGTTCTGTATGAATTTTTTTCTTAATCTT
TTTATTTTAAAGATAAGAATATTTCATAAAAAAGAAAGAAGATAACTGAAATGATCTAAAGTCAACCTTTTGTCTTACAATCAATT

TGTAAAAAATCAAAATTGAAACATCGTATAATTTTAAATAATATAGGGGCGGATGTAGCCAAGTGGATCAAGGCAGTGGATTGTG
AATC
>*Leucophyllum laevigatum* 4. (trnH-psbA) Mexico, La Zarca, Dgo (ZAR). L_laevigatumZar4
CGTAATGCTCATAACTTCCCTCTAGATCTAGCTGCTATTGAAGCTCCAACAAATGGATAAGACTTGGTCTTAGTGTATAGGGGTTTT
TGAAAATAGAATATATAAATATAAGGAGCAATAAACTCTTTCTTGTTCTATCAAGAGGGGTTATTGCTCCTTTATTTTCTTTTCAAT
GAGTAGTATTTTGTTAAAAGGGTTTTAAGGGATTGATTAATGATGGAGTATTTTTCTTTCGTTCTGTATGAATTTTTTTCTTAATCTT
TTTATTTTAAAGATAAGAATATTTCATAAAAAAGAAAGAAGATAACTGAAATGATCTAAAGTCAACCTTTTGTCTTACAATCAATT
TGTAAAAAATCAAAATTGAAACATCGTATAATTTTAAATAATATAGGGGCGGATGTAGCCAAGTGGATCAAGGCAGTGGATTGTG
AATC
>*Leucophyllum laevigatum* 5. (trnH-psbA) Mexico, La Zarca, Dgo (ZAR). L_laevigatumZar5
ATCTAGCTGCTATTGAAGCTCCAACAAATGGATAAGACTTGGTCTTAGTGTATAGGGGTTTTTGAAAATAGAATATATAAATATAA
GGAGCAATAAACTCTTTCTTGTTCTATCAAGAGGGGTTATTGCTCCTTTATTTTCTTTTCAATGAGTAGTATTTTGTTAAAAGGGTTT
TAAGGGATTGATTAATGATGGAGTATTTTTCTTTCGTTCTGTATGAATTTTTTTCTTAATCTTTTTATTTTAAAGATAAGAATATTTC
ATAAAAAAGAAAGAAGATAACTGAAATGATCTAAAGTCAACCTTTTGTCTTACAATCAATTTGTAAAAAATCAAAATTGAAACAT
CGTATAATTTTAAATAATATAGGGGCGGATGTAGCCAAGTGGATCAAGGCAGTGGATTGTGAATC
>*Leucophyllum laevigatum* 1. (trnH-psbA) Mexico, Santa María del Oro, Dgo (StaMO). L_laevigatumMao1
CGTAATGCTCATAACTTCCCTCTAGATCTAGCTGCTATTGAAGCTCCAACAAATGGATAAGACTTGGTCTTAGTGTATAGGGGTTTT
TGAAAATAGAATATATAAATATAAGGAGCAATAAACTCTTTCTTGTTCTATCAAGAGGGGTTATTGCTCCTTTATTTTCTTTTCAAT
GAGTAGTATTTTGTTAAAAGGGTTTTAAGGGATTGATTAATGATGGAGTATTTTTCTTTCGTTCTGTATGAATTTTTTTCTTAATCTT
TTTATTTTAAAGATAAGAATATTTCATAAAAAAGAAAGAAGATAACTGAAATGATCTAAAGTCAACCTTTTGTCTTACAATCAATT
TGTAAAAAATCAAAATTGAAACATCGTATAATTTTAAATAATATAGGGGCGGATGTAGCCAAGTGGATCAAGGCAGTGGATTGTG
AATC
>*Leucophyllum laevigatum* 3. (trnH-psbA) Mexico, Santa María del Oro, Dgo (StaMO). L_laevigatumMao3
CGTAATGCTCATAACTTCCCTCTAGATCTAGCTGCTATTGAAGCTCCAACAAATGGATAAGACTTGGTCTTAGTGTATAGGGGTTTT
TGAAAATAGAATATATAAATATAAGGAGCAATAAACTCTTTCTTGTTCTATCAAGAGGGGTTATTGCTCCTTTATTTTCTTTTCAAT
GAGTAGTATTTTGTTAAAAGGGTTTTAAGGGATTGATTAATGATGGAGTATTTTTCTTTCGTTCTGTATGAATTTTTTTCTTAATCTT
TTTATTTTAAAGATAAGAATATTTCATAAAAAAGAAAGAAGATAACTGAAATGATCTAAAGTCAACCTTTTGTCTTACAATCAATT
TGTAAAAAATCAAAATTGAAACATCGTATAATTTTAAATAATATAGGGGCGGATGTAGCCAAGTGGATCAAGGCAGTGGATTGTG
AATC
>*Leucophyllum laevigatum* 5. (trnH-psbA) Mexico, Santa María del Oro, Dgo (StaMO).L_laevigatumMao5
CGTAATGCTCATAACTTCCCTCTAGATCTAGCTGCTATTGAAGCTCCAACAAATGGATAAGACTTGGTCTTAGTGTATAGGGGTTTT
TGAAAATAGAATATATAAATATAAGGAGCAATAAACTCTTTCTTGTTCTATCAAGAGGGGTTATTGCTCCTTTATTTTCTTTTCAAT
GAGTAGTATTTTGTTAAAAGGGTTTTAAGGGATTGATTAATGATGGAGTATTTTTCTTTCGTTCTGTATGAATTTTTTTCTTAATCTT
TTTATTTTAAAGATAAGAATATTTCATAAAAAAGAAAGAAGATAACTGAAATGATCTAAAGTCAACCTTTTGTCTTACAATCAATT
TGTAAAAAATCAAAATTGAAACATCGTATAATTTTAAATAATATAGGGGCGGATGTAGCCAAGTGGATCAAGGCAGTGGATTGTG
AATC
>*Leucophyllum laevigatum* 7. (trnH-psbA) Mexico, Santa María del Oro, Dgo (StaMO). L_laevigatumMao7
CGTAATGCTCATAACTTCCCTCTAGATCTAGCTGCTATTGAAGCTCCAACAAATGGATAAGACTTGGTCTTAGTGTATAGGGGTTTT
TGAAAATAGAATATATAAATATAAGGAGCAATAAACTCTTTCTTGTTCTATCAAGAGGGGTTATTGCTCCTTTATTTTCTTTTCAAT
GAGTAGTATTTTGTTAAAAGGGTTTTAAGGGATTGATTAATGATGGAGTATTTTTCTTTCGTTCTGTATGAATTTTTTTCTTAATCTT
TTTATTTTAAAGATAAGAATATTTCATAAAAAAGAAAGAAGATAACTGAAATGATCTAAAGTCAACCTTTTGTCTTACAATCAATT

TGTAAAAAATCAAAATTGAAACATCGTATAATTTTAAATAATATAGGGGCGGATGTAGCCAAGTGGATCAAGGCAGTGGATTGTG
AATC
>*Leucophyllum laevigatum* 10. (trnH-psbA) Mexico, Santa María del Oro, Dgo (StaMO). L_laevigatumMao10
CGTAATGCTCATAACTTCCCTCTAGATCTAGCTGCTATTGAAGCTCCAACAAATGGATAAGACTTGGTCTTAGTGTATAGGGGTTTT
TGAAAATAGAATATATAAATATAAGGAGCAATAAACTCTTTCTTGTTCTATCAAGAGGGGTTATTGCTCCTTTATTTTCTTTTCAAT
GAGTAGTATTTTGTTAAAAGGGTTTTAAGGGATTGATTAATGATGGAGTATTTTTCTTTCGTTCTGTATGAATTTTTTTCTTAATCTT
TTTATTTTAAAGATAAGAATATTTCATAAAAAAGAAAGAAGATAACTGAAATGATCTAAAGTCAACCTTTTGTCTTACAATCAATT
TGTAAAAAATCAAAATTGAAACATCGTATAATTTTAAATAATATAGGGGCGGATGTAGCCAAGTGGATCAAGGCAGTGGATTGTG
AATC
>*Leucophyllum laevigatum* 1. (trnH-psbA) L_laevigatumHpa1
CGTAATGCTCATAACTTCCCTCTAGATCTAGCTGCTATTGAAGCTCCAACAAATGGATAAGACTTGGTCTTAGTGTATAGGGGTTTT
TGAAAATAGAATATATAAATATAAGGAGCAATAAACTCTTTCTTGTTCTATCAAGAGGGGTTATTGCTCCTTTATTTTCTTTTCAAT
GAGTAGTATTTTGTTAAAAGGGTTTTAAGGGATTGATTAATGATGGAGTATTTTTCTTTCGTTCTGTATGAATTTTTTTCTTAATCTT
TTTATTTTAAAGATAAGAATATTTCATAAAAAAGAAAGAAGATAACTGAAATGATCTAAAGTCAACCTTTTGTCTTACAATCAATT
TGTAAAAAATCAAAATTGAAACATCGTATAATTTTAAATAATATAGGGGCGGATGTAGCCAAGTGGATCAAGGCAGTGGATTGTG
AATC
>*Leucophyllum laevigatum* 5. (trnH-psbA) L_laevigatumHpa5
CGTAATGCTCATAACTTCCCTCTAGATCTAGCTGCTATTGAAGCTCCAACAAATGGATAAGACTTGGTCTTAGTGTATAGGGGTTTT
TGAAAATAGAATATATAAATATAAGGAGCAATAAACTCTTTCTTGTTCTATCAAGAGGGGTTATTGCTCCTTTATTTTCTTTTCAAT
GAGTAGTATTTTGTTAAAAGGGTTTTAAGGGATTGATTAATGATGGAGTATTTTTCTTTCGTTCTGTATGAATTTTTTTCTTAATCTT
TTTATTTTAAAGATAAGAATATTTCATAAAAAAGAAAGAAGATAACTGAAATGATCTAAAGTCAACCTTTTGTCTTACAATCAATT
TGTAAAAAATCAAAATTGAAACATCGTATAATTTTAAATAATATAGGGGCGGATGTAGCCAAGTGGATCAAGGCAGTGGATTGTG
AATC
>*Leucophyllum laevigatum* 11. (trnH-psbA) L_laevigatumHpa11
CGTAATGCTCATAACTTCCCTCTAGATCTAGCTGCTATTGAAGCTCCAACAAATGGATAAGACTTGGTCTTAGTGTATAGGGGTTTT
TGAAAATAGAATATATAAATATAAGGAGCAATAAACTCTTTCTTGTTCTATCAAGAGGGGTTATTGCTCCTTTATTTTCTTTTCAAT
GAGTAGTATTTTGTTAAAAGGGTTTTAAGGGATTGATTAATGATGGAGTATTTTTCTTTCGTTCTGTATGAATTTTTTTCTTAATCTT
TTTATTTTAAAGATAAGAATATTTCATAAAAAAGAAAGAAGATAACTGAAATGATCTAAAGTCAACCTTTTGTCTTACAATCAATT
TGTAAAAAATCAAAATTGAAACATCGTATAATTTTAAATAATATAGGGGCGGATGTAGCCAAGTGGATCAAGGCAGTGGATTGTG
AATC
>*Leucophyllum laevigatum* 17. (trnH-psbA) L_laevigatumHpa17
CGTAATGCTCATAACTTCCCTCTAGATCTAGCTGCTATTGAAGCTCCAACAAATGGATAAGACTTGGTCTTAGTGTATAGGGGTTTT
TGAAAATAGAATATATAAATATAAGGAGCAATAAACTCTTTCTTGTTCTATCAAGAGGGGTTATTGCTCCTTTATTTTCTTTTCAAT
GAGTAGTATTTTGTTAAAAGGGTTTTAAGGGATTGATTAATGATGGAGTATTTTTCTTTCGTTCTGTATGAATTTTTTTCTTAATCTT
TTTATTTTAAAGATAAGAATATTTCATAAAAAAGAAAGAAGATAACTGAAATGATCTAAAGTCAACCTTTTGTCTTACAATCAATT
TGTAAAAAATCAAAATTGAAACATCGTATAATTTTAAATAATATAGGGGCGGATGTAGCCAAGTGGATCAAGGCAGTGGATTGTG
AATC
>*Leucophyllum laevigatum* 22. (trnH-psbA) L_laevigatumHpa22
CGTAATGCTCATAACTTCCCTCTAGATCTAGCTGCTATTGAAGCTCCAACAAATGGATAAGACTTGGTCTTAGTGTATAGGGGTTTT
TGAAAATAGAATATATAAATATAAGGAGCAATAAACTCTTTCTTGTTCTATCAAGAGGGGTTATTGCTCCTTTATTTTCTTTTCAAT
GAGTAGTATTTTGTTAAAAGGGTTTTAAGGGATTGATTAATGATGGAGTATTTTTCTTTCGTTCTGTATGAATTTTTTTCTTAATCTT
TTTATTTTAAAGATAAGAATATTTCATAAAAAAGAAAGAAGATAACTGAAATGATCTAAAGTCAACCTTTTGTCTTACAATCAATT

TGTAAAAAATCAAAATTGAAACATCGTATAATTTTAAATAATATAGGGGCGGATGTAGCCAAGTGGATCAAGGCAGTGGATTGTG
AATC
>*Leucophyllum laevigatum* 1. (trnH-psbA) Mexico, Trópico de Cáncer, Zac (TCA). L_laevigatumTca1
CGTAATGCTCATAACTTCCCTCTAGATCTAGCTGCTATTGAAGCTCCAACAAATGGATAAGACTTGGTCTTAGTGTATAGGGGTTTT
TGAAAATAGAATATATAAATATAAGGAGCAATAAACTCTTTCTTGTTCTATCAAGAGGGGTTATTGCTCCTTTATTTTCTTTTCAAT
GAGTAGTATTTTGTTAAAAGGGTTTAAGGGATTGATTAATGATGGAGTATTTTTCTTTCGTTCTGTATGAATTTTTTTCTTAATCTT
TTTATTTTAAAGATAAGAATATTTCATAAAAAAGAAAGAAGATAACTGAAATGATCTAAAGTCAACCTTTTGTCTTACAATCAATT
TGTAAAAAATCAAAATTGAAACATCGTATAATTTTAAATAATATAGGGGCGGATGTAGCCAAGTGGATCAAGGCAGTGGATTGTG
AATC
>*Leucophyllum laevigatum* 3. (trnH-psbA) Mexico, Trópico de Cáncer, Zac (TCA). L_laevigatumTca3
CGTAATGCTCATAACTTCCCTCTAGATCTAGCTGCTATTGAAGCTCCAACAAATGGATAAGACTTGGTCTTAGTGTATAGGGGTTTT
TGAAAATAGAATATATAAATATAAGGAGCAATAAACTCTTTCTTGTTCTATCAAGAGGGGTTATTGCTCCTTTATTTTCTTTTCAAT
GAGTAGTATTTTGTTAAAAGGGTTTAAGGGATTGATTAATGATGGAGTATTTTTCTTTCGTTCTGTATGAATTTTTTTCTTAATCTT
TTTATTTTAAAGATAAGAATATTTCATAAAAAAGAAAGAAGATAACTGAAATGATCTAAAGTCAACCTTTTGTCTTACAATCAATT
TGTAAAAAATCAAAATTGAAACATCGTATAATTTTAAATAATATAGGGGCGGATGTAGCCAAGTGGATCAAGGCAGTGGATTGTG
AATC
>*Leucophyllum laevigatum* 6. (trnH-psbA) Mexico, Trópico de Cáncer, Zac (TCA). L_laevigatumTca6
CGTAATGCTCATAACTTCCCTCTAGATCTAGCTGCTATTGAAGCTCCAACAAATGGATAAGACTTGGTCTTAGTGTATAGGGGTTTT
TGAAAATAGAATATATAAATATAAGGAGCAATAAACTCTTTCTTGTTCTATCAAGAGGGGTTATTGCTCCTTTATTTTCTTTTCAAT
GAGTAGTATTTTGTTAAAAGGGTTTAAGGGATTGATTAATGATGGAGTATTTTTCTTTCGTTCTGTATGAATTTTTTTCTTAATCTT
TTTATTTTAAAGATAAGAATATTTCATAAAAAAGAAAGAAGATAACTGAAATGATCTAAAGTCAACCTTTTGTCTTACAATCAATT
TGTAAAAAATCAAAATTGAAACATCGTATAATTTTAAATAATATAGGGGCGGATGTAGCCAAGTGGATCAAGGCAGTGGATTGTG
AATC
>*Leucophyllum laevigatum* 9. (trnH-psbA) Mexico, Trópico de Cáncer, Zac (TCA). L_laevigatumTca9
CGTAATGCTCATAACTTCCCTCTAGATCTAGCTGCTATTGAAGCTCCAACAAATGGATAAGACTTGGTCTTAGTGTATAGGGGTTTT
TGAAAATAGAATATATAAATATAAGGAGCAATAAACTCTTTCTTGTTCTATCAAGAGGGGTTATTGCTCCTTTATTTTCTTTTCAAT
GAGTAGTATTTTGTTAAAAGGGTTTAAGGGATTGATTAATGATGGAGTATTTTTCTTTCGTTCTGTATGAATTTTTTTCTTAATCTT
TTTATTTTAAAGATAAGAATATTTCATAAAAAAGAAAGAAGATAACTGAAATGATCTAAAGTCAACCTTTTGTCTTACAATCAATT
TGTAAAAAATCAAAATTGAAACATCGTATAATTTTAAATAATATAGGGGCGGATGTAGCCAAGTGGATCAAGGCAGTGGATTGTG
AATC
>*Leucophyllum laevigatum* 12. (trnH-psbA) Mexico, Trópico de Cáncer, Zac (TCA). L_laevigatumTca12
CGTAATGCTCATAACTTCCCTCTAGATCTAGCTGCTATTGAAGCTCCAACAAATGGATAAGACTTGGTCTTAGTGTATAGGGGTTTT
TGAAAATAGAATATATAAATATAAGGAGCAATAAACTCTTTCTTGTTCTATCAAGAGGGGTTATTGCTCCTTTATTTTCTTTTCAAT
GAGTAGTATTTTGTTAAAAGGGTTTAAGGGATTGATTAATGATGGAGTATTTTTCTTTCGTTCTGTATGAATTTTTTTCTTAATCTT
TTTATTTTAAAGATAAGAATATTTCATAAAAAAGAAAGAAGATAACTGAAATGATCTAAAGTCAACCTTTTGTCTTACAATCAATT
TGTAAAAAATCAAAATTGAAACATCGTATAATTTTAAATAATATAGGGGCGGATGTAGCCAAGTGGATCAAGGCAGTGGATTGTG
AATC
>*Leucophyllum laevigatum* 1. (trnH-psbA) L_laevigatumRoc1
CGTAATGCTCATAACTTCCCTCTAGATCTAGCTGCTATTGAAGCTCCAACAAATGGATAAGACTTGGTCTTAGTGTATAGGGGTTTT
TGAAAATAGAATATATAAATATAAGGAGCAATAAACTCTTTCTTGTTCTATCAAGAGGGGTTATTGCTCCTTTATTTTCTTTTCAAT
GAGTAGTATTTTATTAAAAGGGTTTAAGGGATTGATTAATGATGGAGTATTTTTCTTTCGTTCTGTATGAATTTTTTTCTTAATCTT
TTTATTTTAAAGATAAGAATATTTCATAAAAAAGAAAGAAGATAACTGAAATGATCTAAAGTCAACCTTTTGTCTTACAATCAATT

TGTAAAAAATCAAAATTGAAACATCGTATAATTTTAAATAATATAGGGGCGGATGTAGCCAAGTGGATCAAGGCAGTGGATTGTG
AATC
>*Leucophyllum laevigatum* 3. (trnH-psbA) L_laevigatumRoc3
CGTAATGCTCATAACTTCCCTCTAGATCTAGCTGCTATTGAAGCTCCAACAAATGGATAAGACTTGGTCTTAGTGTATAGGGGTTTT
TGAAAATAGAATATATAAATATAAGGAGCAATAAACTCTTTCTTGTTCTATCAAGAGGGGTTATTGCTCCTTTATTTTCTTTTCAAT
GAGTAGTATTTTATTAAAAGGGTTTTAAGGGATTGATTAATGATGGAGTATTTTTCTTTCGTTCTGTATGAATTTTTTTCTTAATCTT
TTTATTTTAAAGATAAGAATATTTCATAAAAAAGAAAGAAGATAACTGAAATGATCTAAAGTCAACCTTTTGTCTTACAATCAATT
TGTAAAAAATCAAAATTGAAACATCGTATAATTTTAAATAATATAGGGGCGGATGTAGCCAAGTGGATCAAGGCAGTGGATTGTG
AATC
>*Leucophyllum laevigatum* 5. (trnH-psbA) L_laevigatumRoc5
CGTAATGCTCATAACTTCCCTCTAGATCTAGCTGCTATTGAAGCTCCAACAAATGGATAAGACTTGGTCTTAGTGTATAGGGGTTTT
TGAAAATAGAATAGAATATATAAATATAAGGAGCAATAAACTCTTTCTTGTTCTATCAAGAGGGGTTATTGCTCCTTTATTTTCTTT
TCAATGAGTAGTATTTTGTTAAAAGGGTTTTAAGGGATTGATTAATGATGGAGTATTTTTCTTTCGTTCTGTATGAATTTTTTTCTTA
ATCTTTTTATTTTAAAGATAAGAATATTTCATAAAAAAGAAAGAAGATAACTGAAATGATCTAAAGTCAACCTTTTGTCTTACAAT
CAATTTGTAAAAAATCAAAATTGAAACATCGTATAATTTTAAATAATATAGGGGCGGATGTAGCCAAGTGGATCAAGGCAGTGGA
TTGTGAATC
>*Leucophyllum laevigatum* 7. (trnH-psbA) L_laevigatumRoc7
CGTAATGCTCATAACTTCCCTCTAGATCTAGCTGCTATTGAAGCTCCAACAAATGGATAAGACTTGGTCTTAGTGTATAGGGGTTTT
TGAAAATAGAATATATAAATATAAGGAGCAATAAACTCTTTCTTGTTCTATCAAGAGGGGTTATTGCTCCTTTATTTTCTTTTCAAT
GAGTAGTATTTTGTTAAAAGGGTTTTAAGGGATTGATTAATGATGGAGTATTTTTCTTTCGTTCTGTATGAATTTTTTTCTTAATCTT
TTTATTTTAAAGATAAGAATATTTCATAAAAAAGAAAGAAGATAACTGAAATGATCTAAAGTCAACCTTTTGTCTTACAATCAATT
TGTAAAAAATCAAAATTGAAACATCGTATAATTTTAAATAATATAGGGGCGGATGTAGCCAAGTGGATCAAGGCAGTGGATTGTG
AATC
>*Leucophyllum laevigatum* 9. (trnH-psbA) L_laevigatumRoc9
CGTAATGCTCATAACTTCCCTCTAGATCTAGCTGCTATTGAAGCTCCAACAAATGGATAAGACTTGGTCTTAGTGTATAGGGGTTTT
TGAAAATAGAATATATAAATATAAGGAGCAATAAACTCTTTCTTGTTCTATCAAGAGGGGTTATTGCTCCTTTATTTTCTTTTCAAT
GAGTAGTATTTTGTTAAAAGGGTTTTAAGGGATTGATTAATGATGGAGTATTTTTCTTTCGTTCTGTATGAATTTTTTTCTTAATCTT
TTTATTTTAAAGATAAGAATATTTCATAAAAAAGAAAGAAGATAACTGAAATGATCTAAAGTCAACCTTTTGTCTTACAATCAATT
TGTAAAAAATCAAAATTGAAACATCGTATAATTTTAAATAATATAGGGGCGGATGTAGCCAAGTGGATCAAGGCAGTGGATTGTG
AATC
>*Leucophyllum laevigatum* 1. (trnH-psbA) L_laevigatumMaz1
CGTAATGCTCATAACTTCCCTCTAGATCTAGCTGCTATTGAAGCTCCAACAAATGGATAAGACTTGGTCTTAGTGTATAGGGGTTTT
TGAAAATAGAATATATAAATATAAGGAGCAATAAACTCTTTCTTGTTCTATCAAGAGGGGTTATTGCTCCTTTATTTTCTTTTCAAT
GAGTAGTATTTTGTTAAAAGGGTTTTAAGGGATTGATTAATGATGGAGTATTTTTCTTTCGTTCTGTATGAATTTTTTTCTTAATCTT
TTTATTTTAAAGATAAGAATATTTCATAAAAAAGAAAGAAGATAACTGAAATGATCTAAAGTCAACCTTTTGTCTTACAATCAATT
TGTAAAAAATCAAAATTGAAACATCGTATAATTTTAAATAATATAGGGGCGGATGTAGCCAAGTGGATCAAGGCAGTGGATTGTG
AATC
>*Leucophyllum laevigatum* 2. (trnH-psbA) L_laevigatumMaz2
CGTAATGCTCATAACTTCCCTCTAGATCTAGCTGCTATTGAAGCTCCAACAAATGGATAAGACTTGGTCTTAGTGTATAGGGGTTTT
TGAAAATAGAATATATAAATATAAGGAGCAATAAACTCTTTCTTGTTCTATCAAGAGGGGTTATTGCTCCTTTATTTTCTTTTCAAT
GAGTAGTATTTTATTAAAAGGGTTTTAAGGGATTGATTAATGATGGAGTATTTTTCTTTCGTTCTGTATGAATTTTTTTCTTAATCTT
TTTATTTTAAAGATAAGAATATTTCATAAAAAAGAAAGAAGATAACTGAAATGATCTAAAGTCAACCTTTTGTCTTACAATCAATT

TGTAAAAAATCAAAATTGAAACATCGTATAATTTTAAATAATATAGGGGCGGATGTAGCCAAGTGGATCAAGGCAGTGGATTGTG
AATC
>*Leucophyllum laevigatum* 3. (trnH-psbA) L_laevigatumMaz3
CGTAATGCTCATAACTTCCCTCTAGATCTAGCTGCTATTGAAGCTCCAACAAATGGATAAGACTTGGTCTTAGTGTATAGGGGTTTT
TGAAAATAGAATATATAAATATAAGGAGCAATAAACTCTTTCTTGTTCTATCAAGAGGGGTTATTGCTCCTTTATTTTCTTTTCAAT
GAGTAGTATTTTATTAAAAGGGTTTTAAGGGATTGATTAATGATGGAGTATTTTTCTTTCGTTCTGTATGAATTTTTTTCTTAATCTT
TTTATTTTAAAGATAAGAATATTTCATAAAAAAGAAAGAAGATAACTGAAATGATCTAAAGTCAACCTTTTGTCTTACAATCAATT
TGTAAAAAATCAAAATTGAAACATCGTATAATTTTAAATAATATAGGGGCGGATGTAGCCAAGTGGATCAAGGCAGTGGATTGTG
AATC
>*Leucophyllum laevigatum* 1. (trnH-psbA) Mexico, Mapimí, Dgo (MAP). L_laevigatumMap1
CGTAATGCTCATAACTTCCCTCTAGATCTAGCTGCTATTGAAGCTCCAACAAATGGATAAGACTTGGTCTTAGTGTATAGGGGTTTT
TGAAAATAGAATATATAAATATAAGGAGCAATAAACTCTTTCTTGTTCTATCAAGAGGGGTTATTGCTCCTTTATTTTCTTTTCAAT
GAGTAGTATTTTGTTAAAAGGGTTTTAAGGGATTGATTAATGATGGAGTATTTTTCTTTCGTTCTGTATGAATTTTTTTCTTAATCTT
TTTATTTTAAAGATAAGAATATTTCATAAAAAAGAAAGAAGATAACTGAAATGATCTAAAGTCAACCTTTTGTCTTACAATCAATT
TGTAAAAAATCAAAATTGAAACATCGTATAATTTTAAATAATATAGGGGCGGATGTAGCCAAGTGGATCAAGGCAGTGGATTGTG
AATC
>*Leucophyllum laevigatum* 4. (trnH-psbA) Mexico, Mapimí, Dgo (MAP). L_laevigatumMap4
CGTAATGCTCATAACTTCCCTCTAGATCTAGCTGCTATTGAAGCTCCAACAAATGGATAAGACTTGGTCTTAGTGTATAGGGGTTTT
TGAAAATAGAATATATAAATATAAGGAGCAATAAACTCTTTCTTGTTCTATCAAGAGGGGTTATTGCTCCTTTATTTTCTTTTCAAT
GAGTAGTATTTTGTTAAAAGGGTTTTAAGGGATTGATTAATGATGGAGTATTTTTCTTTCGTTCTGTATGAATTTTTTTCTTAATCTT
TTTATTTTAAAGATAAGAATATTTCATAAAAAAGAAAGAAGATAACTGAAATGATCTAAAGTCAACCTTTTGTCTTACAATCAATT
TGTAAAAAATCAAAATTGAAACATCGTATAATTTTAAATAATATAGGGGCGGATGTAGCCAAGTGGATCAAGGCAGTGGATTGTG
AATC
>*Leucophyllum laevigatum* 5. (trnH-psbA) Mexico, Mapimí, Dgo (MAP). L_laevigatumMap5
CGTAATGCTCATAACTTCCCTCTAGATCTAGCTGCTATTGAAGCTCCAACAAATGGATAAGACTTGGTCTTAGTGTATAGGGGTTTT
TGAAAATAGAATATATAAATATAAGGAGCAATAAACTCTTTCTTGTTCTATCAAGAGGGGTTATTGCTCCTTTATTTTCTTTTCAAT
GAGTAGTATTTTGTTAAAAGGGTTTTAAGGGATTGATTAATGATGGAGTATTTTTCTTTCGTTCTGTATGAATTTTTTTCTTAATCTT
TTTATTTTAAAGATAAGAATATTTCATAAAAAAGAAAGAAGATAACTGAAATGATCTAAAGTCAACCTTTTGTCTTACAATCAATT
TGTAAAAAATAAAAATTGAAACATCCTATGATTTTAAATAATATAGGGGCGGATGTAGCCAAGTGGATCAAGGCAGTGGATTGTG
AATC
>*Leucophyllum laevigatum* 1. (trnH-psbA) L_laevigatumBon1
CGTAATGCTCATAACTTCCCTCTAGATCTAGCTGCTATTGAAGCTCCAACAAATGGATAAGACTTGGTCTTAGTGTATAGGGGTTTT
TGAAAATAGAATATATAAATATAAGGAGCAATAAACTCTTTCTTGTTCTATCAAGAGGGGTTATTGCTCCTTTATTTTCTTTTCAAT
GAGTAGTATTTTGTTAAAAGGGTTTTAAGGGATTGATTAATGATGGAGTATTTTTTTTTCGTTCTGTATGAATTTTTTTCTTAATCTT
TTTATTTTAAAGATAAGAATATTTCATAAAAAAGAAAGAAGATAACTGAAATGATCTAAAGTCAACCTTTTGTCTTACAATCAATT
TGTAAAAAATAAAAATTGAAACATCATATGATTTTAAATAATATAGGGGCGGATGTAGCCAAGTGGATCAAGGCAGTGGATTGTG
AATC
>*Leucophyllum laevigatum* 4. (trnH-psbA) L_laevigatumBon4
CGTAATGCTCATAACTTCCCTCTAGATCTAGCTGCTATTGAAGCTCCAACAAATGGATAAGACTTGGTCTTAGTGTATAGGGGTTTT
TGAAAATAGAATATATAAATATAAGGAGCAATAAACTCTTTCTTGTTCTATCAAGAGGGGTTATTGCTCCTTTATTTTCTTTTCAAT
GAGTAGTATTTTATTAAAAGGGTTTTAAGGGATTGATTAATGATGGAGTATTTTTCTTTCGTTCTGTATGAATTTTTTTCTTAATCTT
TTTATTTTAAAGATAAGAATATTTCATAAAAAAGAAAGAAGATAACTGAAATGATCTAAAGTCAACCTTTTGTCTTACAATCAATT

TGTAAAAAATCAAAATTGAAACATCGTATAATTTTAAATAATATAGGGGCGGATGTAGCCAAGTGGATCAAGGCAGTGGATTGTG
AATC
>*Leucophyllum laevigatum* 6. (trnH-psbA) L_laevigatumBon6
CGTAATGCTCATAACTTCCCTCTAGATCTAGCTGCTATTGAAGCTCCAACAAATGGATAAGACTTGGTCTTAGTGTATAGGGGTTTT
TGAAAATAGAATATATAAATATAAGGAGCAATAAACTCTTTCTTGTTCTATCAAGAGGGGTTATTGCTCCTTTATTTTCTTTTCAAT
GAGTAGTATTTTGTTAAAAGGGTTTTAAGGGATTGATTAATGATGGAGTATTTTTTTTTCGTTCTGTATGAATTTTTTTCTTAATCTT
TTTATTTTAAAGATAAGAATATTTCATAAAAAAGAAAGAAGATAACTGAAATGATCTAAAGTCAACCTTTTGTCTTACAATCAATT
TGTAAAAAATAAAAATTGAAACATCATATGATTTTAAATAATATAGGGGCGGATGTAGCCAAGTGGATCAAGGCAGTGGATTGTG
AATC
>*Leucophyllum laevigatum* 1. (psbK) Mexico, San Juan del Rio, Dgo (ATO). L_laevigatumAto1
GAGTAAGCATTACACAATCTCCAAGATGATTTTTTGGAAAAAAAAAGAGAATAGATCTTCCATTTTTCTATCACATATCCTATTTTG
ACACCACCACCACGAAATCAGGTTTTTTTTCATGAATTGTCACAAATTCATTTGTTTTTCATTATCAAGAACTTCTTTCATATCCAAA
TTCGATATTGTGGGAGACCCTAATAGGGTTAGGGTCTTTCACTGGAAAGGGGGTCAAAAATGAGGACATGGGGGTAAGCCGGATT
TATGGCGACTATCCAAGAATTTCAGTGTGCTAATCTAGGATTCATACTCTAAAACTTATCTGATTTTCTCAATTGTTAGGATTTTTCT
CGAAGAAATCATGCATTTTCTAAGATATTATTATTAAGGATCTCATCGAAAACTTACAGCAGCTTGCCAAACA
>*Leucophyllum laevigatum* 9. (psbK) Mexico, San Juan del Rio, Dgo (ATO). L_laevigatumAto9
GAGTAAGCATTACACAATCTCCAAGATGATTTTTTGGAAAAAAAAAGAGAATAGATCTTCCATTTTTCTATCACATATCCTATTTTG
ACACCACCACCACGAAATCAGGTTTTTTTTCATGAATTGTCACAAATTCATTTGTTTTTCATTATCAAGAACTTCTTTCATATCCAA
ATTCGATATTGTGGGAGACCCTAATAGGGTTAGGGTCTTTCACTGGAAAGGGGGTCAAAAATGAGGACATGGGGGTAAGCCGGAT
TTATGGCGACTATCCAAGAATTTCAGTGTGCTAATCTAGGATTCATACTCTAAAACTTATCTGATTTTCTCAATTGTTAGGATTTTTC
TCGAAGAAATCATGCATTTTCTAAGATATTATTATTAAGGATCTCATCGAAAACTTACAGCAGCTTGCCAAACA
>*Leucophyllum laevigatum* 13. (psbK) Mexico, San Juan del Rio, Dgo (ATO) .L_laevigatumAto13
GAGTAAGCATTACACAATCTCCAAGATGATTTTTTGGAAAAAAAAAGAGAATAGATCTTCCATTTTTCTATCACATATCCTATTTTG
ACACCACCACCACGAAATCAGGTTTTTTTTCATGAATTGTCACAAATTCATTTGTTTTTCATTATCAAGAACTTCTTTCATATCCAAA
TTCGATATTGTGGGAGACCCTAATAGGGTTAGGGTCTTTCACTGGAAAGGGGGTCAAAAATGAGGACATGGGGGTAAGCCGGATT
TATGGCGACTATCCAAGAATTTCAGTGTGCTAATCTAGGATTCATACTCTAAAACTTATCTGATTTTCTCAATTGTTAGGATTTTTCT
CGAAGAAATCATGCATTTTCTAAGATATTATTATTAAGGATCTCATCGAAAACTTACAGCAGCTTGCCAAACA
>*Leucophyllum laevigatum* 19. (psbK) Mexico, San Juan del Rio, Dgo (ATO). L_laevigatumAto19
GAGTAAGCATTACACAATCTCCAAGATGATTTTTTGGAAAAAAAAAGAGAATAGATCTTCCATTTTTCTATCACATATCCTATTTTG
ACACCACCACCACGAAATCAGGTTTTTTTTCATGAATTGTCACAAATTCATTTGTTTTTCATTATCAAGAACTTCTTTCATATCCAAA
TTCGATATTGTGGGAGACCCTAATAGGGTTAGGGTCTTTCACTGGAAAGGGGGTCAAAAATGAGGACATGGGGGTAAGCCGGATT
TATGGCGACTATCCAAGAATTTCAGTGTGCTAATCTAGGATTCATACTCTAAAACTTATCTGATTTTCTCAATTGTTAGGATTTTTCT
CGAAGAAATCATGCATTTTCTAAGATATTATTATTAAGGATCTCATCGAAAACTTACAGCAGCTTGCCAAACA
>*Leucophyllum laevigatum* 1. (psbK) Mexico, Rodeo, Dgo (ROD). L_laevigatumRod1
GAGTAAGCATTACACAATCTCCAAGATGATTTTTTGGAAAAAAAAAAGAGAATAGATCTTCCATTTTTCTATCACATATCCTATTTT
GACACCACCACCACGAAATCAGGTTTTTTTTCATGAATTGTCACAAATTCATTTGTTTTTCATTATCAAGAACTTCTTTCATATCCAA
ATTCGATATTGTGGGAGACCCTAATAGGGTTAGGGTCTTTCACTGGAAAGGGGGTCAAAAATGAGGACATGGGGGTAAGCCGGAT
TTATGGCGACTATCCAAGAATTTCAGTGTGCTAATCTAGGATTCATACTCTAAAACTTATCTGATTTTATCAATTGTTAGGATTTTTC
TCGAAGAAATCATGCATTTTCTAAGATATTATTATTAAGGATCTCATCGAAAACTTACAGCAGCTTGCCAAACA
>*Leucophyllum laevigatum* 4. (psbK) Mexico, Rodeo, Dgo (ROD). L_laevigatumRod4
GAGTAAGCATTACACAATCTCCAAGATGATTTTTTGGAAAAAAAAAAGAGAATAGATCTTCCATTTTTCTATCACATATCCTATTTT
GACACCACCACCACGAAATCAGGTTTTTTTTCATGAATTGTCACAAATTCATTTGTTTTTCATTATCAAGAACTTCTTTCATATCCAA

ATTCGATATTGTGGGAGACCCTAATAGGGTTAGGGTCTTTCACTGGAAAGGGGGTCAAAAATGAGGACATGGGGGTAAGCCGGAT
TTATGGCGACTATCCAAGAATTTCAGTGTGCTAATCTAGGATTCATACTCTAAAACTTATCTGATTTTATCAATTGTTAGGATTTTTC
TCGAAGAAATCATGCATTTTCTAAGATATTATTATTAAGGATCTCATCGAAAACTTACAGCAGCTTGCCAAACA
>*Leucophyllum laevigatum* 9. (psbK) Mexico, Rodeo, Dgo (ROD). L_laevigatumRod9
GAGTAAGCATTACACAATCTCCAAGATGATTTTTTGGAAAAAAAAAAGAGAATAGATCTTCCATTTTTCTATCACATATCCTATTTT
GACACCACCACCACGAAATCAGGTTTTTTTTCATGAATTGTCACAAATTCATTTGTTTTTCATTATCAAGAACTTCTTTCATATCCAA
ATTCGATATTGTGGGAGACCCTAATAGGGTTAGGGTCTTTCACTGGAAAGGGGGTCAAAAATGAGGACATGGGGGTAAGCCGGAT
TTATGGCGACTATCCAAGAATTTCAGTGTGCTAATCTAGGATTCATACTCTAAAACTTATCTGATTTTATCAATTGTTAGGATTTTTC
TCGAAGAAATCATGCATTTTCTAAGATATTATTATTAAGGATCTCATCGAAAACTTACAGCAGCTTGCCAAACA
>*Leucophyllum laevigatum* 13. (psbK) Mexico, Rodeo, Dgo (ROD). L_laevigatumRod13
GAGTAAGCATTACACAATCTCCAAGATGATTTTTTGGAAAAAAAAAAGAGAATAGATCTTCCATTTTTCTATCACATATCCTATTTT
GACACCACCACCACGAAATCAGGTTTTTTTTCATGAATTGTCACAAATTCATTTGTTTTTCATTATCAAGAACTTCTTTCATATCCAA
ATTCGATATTGTGGGAGACCCTAATAGGGTTAGGGTCTTTCACTGGAAAGGGGGTCAAAAATGAGGACATGGGGGTAAGCCGGAT
TTATGGCGACTATCCAAGAATTTCAGTGTGCTAATCTAGGATTCATACTCTAAAACTTATCTGATTTTATCAATTGTTAGGATTTTTC
TCGAAGAAATCATGCATTTTCTAAGATATTATTATTAAGGATCTCATCGAAAACTTACAGCAGCTTGCCAAACA
>*Leucophyllum laevigatum* 17. (psbK) Mexico, Rodeo, Dgo (ROD). L_laevigatumRod17
GAGTAAGCATTACACAATCTCCAAGATGATTTTTTGGAAAAAAAAAAAGAGAATAGATCTTCCATTTTTCTATCACATATCCTATTT
TGACACCACCACCACGAAATCAGGTTTTTTTTCATGAATTGTCACAAATTCATTTGTTTTTCATTATCAAGAACTTCTTTCATATCCA
AATTCGATATTGTGGGAGACCCTAATAGGGTTAGGGTCTTTCACTGGAAAGGGGGTCAAAAATGAGGACATGGGGGTAAGCCGGA
TTTATGGCGACTATCCAAGAATTTCAGTGTGCTAATCTAGGATTCATACTCTAAAACTTATCTGATTTTCTCAATTGTTAGGATTTTT
CTCGAAGAAATCATGCATTTTCTAAGATATTATTATTAAGGATCTCATCGAAAACTTACAGCAGCTTGCCAAACA
>*Leucophyllum laevigatum* 1. (psbK) Mexico, La Zarca, Dgo (ZAR). L_laevigatumZar1
GAGTAAGCATTACACAATCTCCAAGATGATTTTTTGGAAAAAAAAAAGAGAATAGATCTTCCATTTTTCTATCACATATCCTATTTTG
ACACCACCACCACGAAATCAGGTTTTTTTTCATGAATTGTCACAAATTCATTTGTTTTTCATTATCAAGAACTTCTTTCATATCCAAA
TTCGATATTGTGGGAGACCCTAATAGGGTTAGGGTCTTTCACTGGAAAGGGGGTCAAAAATGAGGACATGGGGGTAAGCCGGATT
TATGGCGACTATCCAAGAATTTCAGTGTGCTAATCTAGGATTCATACTCTAAAACTTATCTGATTTTCTCAATTGTTAGGATTTTTCT
CGAAGAAATCATGCATTTTCTAAGATATTATTATTAAGGATCTCATCGAAAACTTACAGCAGCTTGCCAAACA
>*Leucophyllum laevigatum* 2. (psbK) Mexico, La Zarca, Dgo (ZAR). L_laevigatumZar2
GAGTAAGCATTACACAATCTCCAAGATGATTTTTTGGAAAAAAAAAAGAGAATAGATCTTCCATTTTTCTATCACATATCCTATTTTG
ACACCACCACCACGAAATCAGGTTTTTTTTCATGAATTGTCACAAATTCATTTGTTTTTCATTATCAAGAACTTCTTTCATATCCAAA
TTCGATATTGTGGGAGACCCTAATAGGGTTAGGGTCTTTCACTGGAAAGGGGGTCAAAAATGAGGACATGGGGGTAAGCCGGATT
TATGGCGACTATCCAAGAATTTCAGTGTGCTAATCTAGGATTCATACTCTAAAACTTATCTGATTTTCTCAATTGTTAGGATTTTTCT
CGAAGAAATCATGCATTTTCTAAGATATTATTATTAAGGATCTCATCGAAAACTTACAGCAGCTTGCCAAACA
>*Leucophyllum laevigatum* 3. (psbK) Mexico, La Zarca, Dgo (ZAR). L_laevigatumZar3
GAGTAAGCATTACACAATCTCCAAGATGATTTTTTGGAAAAAAAAAAGAGAATAGATCTTCCATTTTTCTATCACATATCCTATTTTG
ACACCACCACCACGAAATCAGGTTTTTTTTCATGAATTGTCACAAATTCATTTGTTTTTCATTATCAAGAACTTCTTTCATATCCAAA
TTCGATATTGTGGGAGACCCTAATAGGGTTAGGGTCTTTCACTGGAAAGGGGGTCAAAAATGAGGACATGGGGGTAAGCCGGATT
TATGGCGACTATCCAAGAATTTCAGTGTGCTAATCTAGGATTCATACTCTAAAACTTATCTGATTTTCTCAATTGTTAGGATTTTTCT
CGAAGAAATCATGCATTTTCTAAGATATTATTATTAAGGATCTCATCGAAAACTTACAGCAGCTTGCCAAACA
>*Leucophyllum laevigatum* 5. (psbK) Mexico, La Zarca, Dgo (ZAR). L_laevigatumZar5
GAGTAAGCATTACACAATCTCCAAGATGATTTTTTGGAAAAAAAAAAGAGAATAGATCTTCCATTTTTCTATCACATATCCTATTTTG
ACACCACCACCACGAAATCAGGTTTTTTTTCATGAATTGTCACAAATTCATTTGTTTTTCATTATCAAGAACTTCTTTCATATCCAAA

TTCGATATTGTGGGAGACCCTAATAGGGTTAGGGTCTTTCACTGGAAAGGGGGTCAAAAATGAGGACATGGGGGTAAGCCGGATT
TATGGCGACTATCCAAGAATTTCAGTGTGCTAATCTAGGATTCATACTCTAAAACTTATCTGATTTTCTCAATTGTTAGGATTTTTCT
CGAAGAAATCATGCATTTTCTAAGATATTATTATTAAGGATCTCATCGAAAACTTACAGCAGCTTGCCAAACA
>*Leucophyllum laevigatum* 1. (psbK) Mexico, Santa María del Oro, Dgo (StaMO). L_laevigatumMao1
GAGTAAGCATTACACAATCTCCAAGATGATTTTTTGGAAAAAAAAAGAGAATAGATCTTCCATTTTTCTATCACATATCCTATTTTG
ACACCACCACCACGAAATCAGGTTTTTTTTTCATGAATTGTCACAAATTCATTTGTTTTTCATTATCAAGAACTTCTTTCATATCCAA
ATTCGATATTGTGGGAGACCCTAATAGGGTTAGGGTCTTTCACTGGAAAGGGGGTCAAAAATGAGGACATGGGGGTAAGCCGGAT
TTATGGCGACTATCCAAGAATTTCAGTGTGCTAATCTAGGATTCATACTCTAAAACTTATCTGATTTTCTCAATTGTTAGGATTTTTC
TCGAAGAAATCATGCATTTTCTAAGATATTATTATTAAGGATCTCATCGAAAACTTACAGCAGCTTGCCAAACA
>*Leucophyllum laevigatum* 3. (psbK) Mexico, Santa María del Oro, Dgo (StaMO). L_laevigatumMao3
GAGTAAGCATTACACAATCTCCAAGATGATTTTTTGGAAAAAAAAAGAGAATAGATCTTCCATTTTTCTATCACATATCCTATTTTG
ACACCACCACCACGAAATCAGGTTTTTTTTTCATGAATTGTCACAAATTCATTTGTTTTTCATTATCAAGAACTTCTTTCATATCCAA
ATTCGATATTGTGGGAGACCCTAATAGGGTTAGGGTCTTTCACTGGAAAGGGGGTCAAAAATGAGGACATGGGGGTAAGCCGGAT
TTATGGCGACTATCCAAGAATTTCAGTGTGCTAATCTAGGATTCATACTCTAAAACTTATCTGATTTTCTCAATTGTTAGGATTTTTC
TCGAAGAAATCATGCATTTTCTAAGATATTATTATTAAGGATCTCATCGAAAACTTACAGCAGCTTGCCAAACA
>*Leucophyllum laevigatum* 5. (psbK) Mexico, Santa María del Oro, Dgo (StaMO). L_laevigatumMao5
GAGTAAGCATTACACAATCTCCAAGATGATTTTTTGGAAAAAAAAAGAGAATAGATCTTCCATTTTTCTATCACATATCCTATTTTG
ACACCACCACCACGAAATCAGGTTTTTTTTTCATGAATTGTCACAAATTCATTTGTTTTTCATTATCAAGAACTTCTTTCATATCCAAA
TTCGATATTGTGGGAGACCCTAATAGGGTTAGGGTCTTTCACTGGAAAGGGGGTCAAAAATGAGGACATGGGGGTAAGCCGGATT
TATGGCGACTATCCAAGAATTTCAGTGTGCTAATCTAGGATTCATACTCTAAAACTTATCTGATTTTCTCAATTGTTAGGATTTTTCT
CGAAGAAATCATGCATTTTCTAAGATATTATTATTAAGGATCTCATCGAAAACTTACAGCAGCTTGCCAAACA
>*Leucophyllum laevigatum* 7. (psbK) Mexico, Santa María del Oro, Dgo (StaMO). L_laevigatumMao7
GAGTAAGCATTACACAATCTCCAAGATGATTTTTTGGAAAAAAAAAGAGAATAGATCTTCCATTTTTCTATCACATATCCTATTTTG
ACACCACCACCACGAAATCAGGTTTTTTTTTCATGAATTGTCACAAATTCATTTGTTTTTCATTATCAAGAACTTCTTTCATATCCAA
ATTCGATATTGTGGGAGACCCTAATAGGGTTAGGGTCTTTCACTGGAAAGGGGGTCAAAAATGAGGACATGGGGGTAAGCCGGAT
TTATGGCGACTATCCAAGAATTTCAGTGTGCTAATCTAGGATTCATACTCTAAAACTTATCTGATTTTCTCAATTGTTAGGATTTTTC
TCGAAGAAATCATGCATTTTCTAAGATATTATTATTAAGGATCTCATCGAAAACTTACAGCAGCTTGCCAAACA
>*Leucophyllum laevigatum* 10. (psbK) Mexico, Santa María del Oro, Dgo (StaMO). L_laevigatumMao10
GAGTAAGCATTACACAATCTCCAAGATGATTTTTTGGAAAAAAAAAGAGAATAGATCTTCCATTTTTCTATCACATATCCTATTTTG
ACACCACCACCACGAAATCAGGTTTTTTTTTCATGAATTGTCACAAATTCATTTGTTTTTCATTATCAAGAACTTCTTTCATATCCAA
ATTCGATATTGTGGGAGACCCTAATAGGGTTAGGGTCTTTCACTGGAAAGGGGGTCAAAAATGAGGACATGGGGGTAAGCCGGAT
TTATGGCGACTATCCAAGAATTTCAGTGTGCTAATCTAGGATTCATACTCTAAAACTTATCTGATTTTCTCAATTGTTAGGATTTTTC
TCGAAGAAATCATGCATTTTCTAAGATATTATTATTAAGGATCTCATCGAAAACTTACAGCAGCTTGCCAAACA
>*Leucophyllum laevigatum* 1. (psbK) L_laevigatumHpa1
GAGTAAGCATTACACAATCTCCAAGATGATTTTTTGGAAAAAAAAAGAGAATAGATCTTCCATTTTTCTATCACATATCCTATTTTG
ACACCACCACCACGAAATCAGGTTTTTTTTTCATGAATTGTCACAAATTCATTTGTTTTTCATTATCAAGAACTTCTTTCATATCCAAA
TTCGATATTGTGGGAGACCCTAATAGGGTTAGGGTCTTTCACTGGAAAGGGGGTCAAAAATGAGGACATGGGGGTAAGCCGGATT
TATGGCGACTATCCAAGAATTTCAGTGTGCTAATCTAGGATTCATACTCTAAAACTTATCTGATTTTCTCAATTGTTAGGATTTTTCT
CGAAGAAATCATGCATTTTCTAAGATATTATTATTAAGGATCTCATCGAAAACTTACAGCAGCTTGCCAAACA
>*Leucophyllum laevigatum* 5. (psbK) L_laevigatumHpa5
GAGTAAGCATTACACAATCTCCAAGATGATTTTTTGGAAAAAAAAAGAGAATAGATCTTCCATTTTTCTATCACATATCCTATTTTG
ACACCACCACCACGAAATCAGGTTTTTTTTTCATGAATTGTCACAAATTCATTTGTTTTTCATTATCAAGAACTTCTTTCATATCCAAA

TTCGATATTGTGGGAGACCCTAATAGGGTTAGGGTCTTTCACTGGAAAGGGGGTCAAAAATGAGGACATGGGGGTAAGCCGGATT
TATGGCGACTATCCAAGAATTTCAGTGTGCTAATCTAGGATTCATACTCTAAAACTTATCTGATTTTCTCAATTGTTAGGATTTTTCT
CGAAGAAATCATGCATTTTCTAAGATATTATTATTAAGGATCTCATCGAAAACTTACAGCAGCTTGCCAAACA
>*Leucophyllum laevigatum* 11. (psbK) L_laevigatumHpa11
GAGTAAGCATTACACAATCTCCAAGATGATTTTTTGGAAAAAAAAAGAGAATAGATCTTCCATTTTTCTATCACATATCCTATTTTG
ACACCACCACCACGAAATCAGGTTTTTTTCATGAATTGTCACAAATTCATTTGTTTTCATTATCAAGAACTTCTTTCATATCCAAA
TTCGATATTGTGGGAGACCCTAATAGGGTTAGGGTCTTTCACTGGAAAGGGGGTCAAAAATGAGGACATGGGGGTAAGCCGGATT
TATGGCGACTATCCAAGAATTTCAGTGTGCTAATCTAGGATTCATACTCTAAAACTTATCTGATTTTCTCAATTGTTAGGATTTTTCT
CGAAGAAATCATGCATTTTCTAAGATATTATTATTAAGGATCTCATCGAAAACTTACAGCAGCTTGCCAAACA
>*Leucophyllum laevigatum* 17. (psbK) L_laevigatumHpa17
GAGTAAGCATTACACAATCTCCAAGATGATTTTTTGGAAAAAAAAAGAGAATAGATCTTCCATTTTTCTATCACATATCCTATTTTG
ACACCACCACCACGAAATCAGGTTTTTTTCATGAATTGTCACAAATTCATTTGTTTTCATTATCAAGAACTTCTTTCATATCCAAA
TTCGATATTGTGGGAGACCCTAATAGGGTTAGGGTCTTTCACTGGAAAGGGGGTCAAAAATGAGGACATGGGGGTAAGCCGGATT
TATGGCGACTATCCAAGAATTTCAGTGTGCTAATCTAGGATTCATACTCTAAAACTTATCTGATTTTCTCAATTGTTAGGATTTTTCT
CGAAGAAATCATGCATTTTCTAAGATATTATTATTAAGGATCTCATCGAAAACTTACAGCAGCTTGCCAAACA
>*Leucophyllum laevigatum* 1. (psbK) Mexico, Trópico de Cáncer, Zac (TCA). L_laevigatumTca1
GAGTAAGCATTACACAATCTCCAAGATGATTTTTTGGAAAAAAAAAGAGAATAGATCTTCCATTTTTCTATCACATATCCTATTTTG
ACACCACCACCACGAAATCAGGTTTTTTTCATGAATTGTCACAAATTCATTTGTTTTCATTATCAAGAACTTCTTTCATATCCAAA
TTCGATATTGTGGGAGACCCTAATAGGGTTAGGGTCTTTCACTGGAAAGGGGGTCAAAAATGAGGACATGGGGGTAAGCCGGATT
TATGGCGACTATCCAAGAATTTCAGTGTGCTAATCTAGGATTCATACTCTAAAACTTATCTGATTTTCTCAATTGTTAGGATTTTTCT
CGAAGAAATCATGCATTTTCTAAGATATTATTATTAAGGATCTCATCGAAAACTTACAGCAGCTTGCCAAACA
>*Leucophyllum laevigatum* 3. (psbK) Mexico, Trópico de Cáncer, Zac (TCA). L_laevigatumTca3
GAGTAAGCATTACACAATCTCCAAGATGATTTTTTGGAAAAAAAAAGAGAATAGATCTTCCATTTTTCTATCACATATCCTATTTTG
ACACCACCACCACGAAATCAGGTTTTTTTCATGAATTGTCACAAATTCATTTGTTTTCATTATCAAGAACTTCTTTCATATCCAAA
TTCGATATTGTGGGAGACCCTAATAGGGTTAGGGTCTTTCACTGGAAAGGGGGTCAAAAATGAGGACATGGGGGTAAGCCGGATT
TATGGCGACTATCCAAGAATTTCAGTGTGCTAATCTAGGATTCATACTCTAAAACTTATCTGATTTTCTCAATTGTTAGGATTTTTCT
CGAAGAAATCATGCATTTTCTAAGATATTATTATTAAGGATCTCATCGAAAACTTACAGCAGCTTGCCAAACA
>*Leucophyllum laevigatum* 6. (psbK) Mexico, Trópico de Cáncer, Zac (TCA). L_laevigatumTca6
GAGTAAGCATTACACAATCTCCAAGATGATTTTTTGGAAAAAAAAAGAGAATAGATCTTCCATTTTTCTATCACATATCCTATTTTG
ACACCACCACCACGAAATCAGGTTTTTTTCATGAATTGTCACAAATTCATTTGTTTTCATTATCAAGAACTTCTTTCATATCCAAA
TTCGATATTGTGGGAGACCCTAATAGGGTTAGGGTCTTTCACTGGAAAGGGGGTCAAAAATGAGGACATGGGGGTAAGCCGGATT
TATGGCGACTATCCAAGAATTTCAGTGTGCTAATCTAGGATTCATACTCTAAAACTTATCTGATTTTCTCAATTGTTAGGATTTTTCT
CGAAGAAATCATGCATTTTCTAAGATATTATTATTAAGGATCTCATCGAAAACTTACAGCAGCTTGCCAAACA
>*Leucophyllum laevigatum* 9. (psbK) Mexico, Trópico de Cáncer, Zac (TCA). L_laevigatumTca9
GAGTAAGCATTACACAATCTCCAAGATGATTTTTTGGAAAAAAAAAGAGAATAGATCTTCCATTTTTCTATCACATATCCTATTTTG
ACACCACCACCACGAAATCAGGTTTTTTTCATGAATTGTCACAAATTCATTTGTTTTCATTATCAAGAACTTCTTTCATATCCAAA
TTCGATATTGTGGGAGACCCTAATAGGGTTAGGGTCTTTCACTGGAAAGGGGGTCAAAAATGAGGACATGGGGGTAAGCCGGATT
TATGGCGACTATCCAAGAATTTCAGTGTGCTAATCTAGGATTCATACTCTAAAACTTATCTGATTTTCTCAATTGTTAGGATTTTTCT
CGAAGAAATCATGCATTTTCTAAGATATTATTATTAAGGATCTCATCGAAAACTTACAGCAGCTTGCCAAACA
>*Leucophyllum laevigatum* 12. (psbK) Mexico, Trópico de Cáncer, Zac (TCA). L_laevigatumTca12
GAGTAAGCATTACACAATCTCCAAGATGATTTTTTGGAAAAAAAAAGAGAATAGATCTTCCATTTTTCTATCACATATCCTATTTTG
ACACCACCACCACGAAATCAGGTTTTTTTCATGAATTGTCACAAATTCATTTGTTTTCATTATCAAGAACTTCTTTCATATCCAAA

TTCGATATTGTGGGAGACCCTAATAGGGTTAGGGTCTTTCACTGGAAAGGGGGTCAAAAATGAGGACATGGGGGTAAGCCGGATT
TATGGCGACTATCCAAGAATTTCAGTGTGCTAATCTAGGATTCATACTCTAAAACTTATCTGATTTTCTCAATTGTTAGGATTTTTCT
CGAAGAAATCATGCATTTTCTAAGATATTATTATTAAGGATCTCATCGAAAACTTACAGCAGCTTGCCAAACA
>*Leucophyllum laevigatum* 1. (psbK) L_laevigatumRoc1
GAGTAAGCATTACACAATCTCCAAGATGATTTTTTGGAAAAAAAAAGAGAATAGATCTTCCATTTTTCTATCACATATCCTATTTTG
ACACCACCACCACGAAATCAGGTTTTTTTTCATGAATTGTCACAAATTCATTTGTTTTTCATTATCAAGAACTTCTTTCATATCCAAA
TTCGATATTGTGGGAGACCCTAATAGGGTTAGGGTCTTTCACTGGAAAGGGGGTCAAAAATGAGGACATGGGGGTAAGCCGGATT
TATGGCGACTATCCAAGAATTTCAGTGTGCTAATCTAGGATTCATACTCTAAAACTTATCTGATTTTCTCAATTGTTAGGATTTTTCT
CGAAGAAATCATGCATTTTCTAAGATATTATTATTAAGGATCTCATCGAAAACTTACAGCAGCTTGCCAAACA
>*Leucophyllum laevigatum* 3. (psbK) L_laevigatumRoc3
GAGTAAGCATTACACAATCTCCAAGATGATTTTTTGGAAAAAAAAAGAGAATAGATCTTCCATTTTTCTATCACATATCCTATTTTG
ACACCACCACCACGAAATCAGGTTTTTTTTCATGAATTGTCACAAATTCATTTGTTTTTCATTATCAAGAACTTCTTTCATATCCAAA
TTCGATATTGTGGGAGACCCTAATAGGGTTAGGGTCTTTCACTGGAAAGGGGGTCAAAAATGAGGACATGGGGGTAAGCCGGATT
TATGGCGACTATCCAAGAATTTCAGTGTGCTAATCTAGGATTCATACTCTAAAACTTATCTGATTTTCTCAATTGTTAGGATTTTTCT
CGAAGAAATCATGCATTTTCTAAGATATTATTATTAAGGATCTCATCGAAAACTTACAGCAGCTTGCCAAACA
>*Leucophyllum laevigatum* 5. (psbK) L_laevigatumRoc5
GAGTAAGCATTACACAATCTCCAAGATGATTTTTTGGAAAAAAAAAGAGAATAGATCTTCCATTTTTCTATCACATATCCTATTTTG
ACACCACCACCACGAAATCAGGTTTTTTTTCATGAATTGTCACAAATTCATTTGTTTTTCATTATCAAGAACTTCTTTCATATCCAAA
TTCGATATTGTGGGAGACCCTAATAGGGTTAGGGTCTTTCACTGGAAAGGGGGTCAAAAATGAGGACATGGGGGTAAGCCGGATT
TATGGCGACTATCCAAGAATTTCAGTGTGCTAATCTAGGATTCATACTCTAAAACTTATCTGATTTTCTCAATTGTTAGGATTTTTCT
CGAAGAAATCATGCATTTTCTAAGATATTATTATTAAGGATCTCATC
>*Leucophyllum laevigatum* 7. (psbK) L_laevigatumRoc7
GAGTAAGCATTACACAATCTCCAAGATGATTTTTTGGAAAAAAAAAGAGAATAGATCTTCCATTTTTCTATCACATATCCTATTTTG
ACACCACCACCACGAAATCAGGTTTTTTTTTCATGAATTGTCACAAATTCATTTGTTTTTCATTATCAAGAACTTCTTTCATATCCAA
ATTCGATATTGTGGGAGACCCTAATAGGGTTAGGGTCTTTCACTGGAAAGGGGGTCAAAAATGAGGACATGGGGGTAAGCCGGAT
TTATGGCGACTATCCAAGAATTTCAGTGTGCTAATCTAGGATTCATACTCTAAAACTTATCTGATTTTCTCAATTGTTAGGATTTTTC
TCGAAGAAATCATGCATTTTCTAAGATATTATTATTAAGGATCTCATCGAAAACTTACAGCAGCTTGCCAAACA
>*Leucophyllum laevigatum* 9. (psbK) L_laevigatumRoc9
GAGTAAGCATTACACAATCTCCAAGATGATTTTTTGGAAAAAAAAAGAGAATAGATCTTCCATTTTTCTATCACATATCCTATTTTG
ACACCACCACCACGAAATCAGGTTTTTTTTTCATGAATTGTCACAAATTCATTTGTTTTTCATTATCAAGAACTTCTTTCATATCCAA
ATTCGATATTGTGGGAGACCCTAATAGGGTTAGGGTCTTTCACTGGAAAGGGGGTCAAAAATGAGGACATGGGGGTAAGCCGGAT
TTATGGCGACTATCCAAGAATTTCAGTGTGCTAATCTAGGATTCATACTCTAAAACTTATCTGATTTTCTCAATTGTTAGGATTTTTC
TCGAAGAAATCATGCATTTTCTAAGATATTATTATTAAGGATCTCATCGAAAACTTACAGCAGCTTGCCAAACA
>*Leucophyllum laevigatum* 2. (psbK) L_laevigatumMaz2
GAGTAAGCATTACACAATCTCCAAGATGATTTTTTGGAAAAAAAAAAGAGAATAGATCTTCCATTTTTCTATCACATATCCTATTTT
GACACCACCACCACGAAATCAGGTTTTTTTTTCATGAATTGTCACAAATTCATTTGTTTTTCATTATCAAGAACTTCTTTCATATCCAA
ATTCGATATTGTGGGAGACCCTAATAGGGTTAGGGTCTTTCACTGGAAAGGGGGTCAAAAATGAGGACATGGGGGTAAGCCGGAT
TTATGGCGACTATCCAAGAATTTCAGTGTGCTAATCTAGGATTCATACTCTAAAACTTATCTGATTTTCTCAATTGTTAGGATTTTTC
TCGAAGAAATCATGCATTTTCTAAGATATTATTATTAAGGATCTCATCGAAAACTTACAGCAGCTTGCCAAACA
>*Leucophyllum laevigatum* 1. (psbK) Mexico, Mapimí, Dgo (MAP). L_laevigatumMap1
GAGTAAGCATTACACAATCTCCAAGATGATTTTTTGGAAAAAAAAAGAGAATAGATCTTCCATTTTTCTATCACATATCCTATTTTG
ACACCACCACCACGAAATCAGGTTTTTTTTCATGAATTGTCACAAATTCATTTGTTTTTCATTATCAAGAACTTCTTTCATATCCAAA

TTCGATATTGTGGGAGACCCTAATAGGGTTAGGGTCTTTCACTGGAAAGGGGGTCAAAAATGAGGACATGGGGGTAAGCCGGATT
TATGGCGACTATCCAAGAATTTCAGTGTGCTAATCTAGGATTCATACTCTAAAACTTATCTGATTTTCTCAATTGTTAGGATTTTTCT
CGAAGAAATCATGCATTTTCTAAGATATTATTATTAAGGATCTCATCGAAAACTTACAGCAGCTTGCCAAACA
>*Leucophyllum laevigatum* 2. (psbK) Mexico, Mapimí, Dgo (MAP). L_laevigatumMap2
GAGTAAGCATTACACAATCTCCAAGATGATTTTTTGGAAAAAAAAAGAGAATAGATCTTCCATTTTTCTATCACATATCCTATTTTG
ACACCACCACCACGAAATCAGGTTTTTTTTCATGAATTGTCACAAATTCATTTGTTTTTCATTATCAAGAACTTCTTTCATATCCAAA
TTCGATATTGTGGGAGACCCTAATAGGGTTAGGGTCTTTCACTGGAAAGGGGGTCAAAAATGAGGACATGGGGGTAAGCCGGATT
TATGGCGACTATCCAAGAATTTCAGTGTGCTAATCTAGGATTCATACTCTAAAACTTATCTGATTTTCTCAATTGTTAGGATTTTTCT
CGAAGAAATCATGCATTTTCTAAGATATTATTATTAAGGATCTCATCGAAAACTTACAGCAGCTTGCCAAACA
>*Leucophyllum laevigatum* 3. (psbK) Mexico, Mapimí, Dgo (MAP). L_laevigatumMap3
GAGTAAGCATTACACAATCTCCAAGATGATTTTTTGGAAAAAAAAAGAGAATAGATCTTCCATTTTTCTATCACATATCCTATTTTG
ACACCACCACCACGAAATCAGGTTTTTTTTCATGAATTGTCACAAATTCATTTGTTTTTCATTATCAAGAACTTCTTTCATATCCAAA
TTCGATATTGTGGGAGACCCTAATAGGGTTAGGGTCTTTCACTGGAAAGGGGGTCAAAAATGAGGACATGGGGGTAAGCCGGATT
TATGGCGACTATCCAAGAATTTCAGTGTGCTAATCTAGGATTCATACTCTAAAACTTATCTGATTTTCTCAATTGTTAGGATTTTTCT
CGAAGAAATCATGCATTTTCTAAGATATTATTATTAAGGATCTCATCGAAAACTTACAGCAGCTTGCCAAACA
>*Leucophyllum laevigatum* 4. (psbK) Mexico, Mapimí, Dgo (MAP). L_laevigatumMap4
GAGTAAGCATTACACAATCTCCAAGATGATTTTTTGGAAAAAAAAAGAGAATAGATCTTCCATTTTTCTATCACATATCCTATTTTG
ACACCACCACCACGAAATCAGGTTTTTTTTCATGAATTGTCACAAATTCATTTGTTTTTCATTATCAAGAACTTCTTTCATATCCAAA
TTCGATATTGTGGGAGACCCTAATAGGGTTAGGGTCTTTCACTGGAAAGGGGGTCAAAAATGAGGACATGGGGGTAAGCCGGATT
TATGGCGACTATCCAAGAATTTCAGTGTGCTAATCTAGGATTCATACTCTAAAACTTATCTGATTTTCTCAATTGTTAGGATTTTTCT
CGAAGAAATCATGCATTTTCTAAGATATTATTATTAAGGATCTCATCGAAAACTTACAGCAGCTTGCCAAACA
>*Leucophyllum laevigatum* 5. (psbK) Mexico, Mapimí, Dgo (MAP). L_laevigatumMap5
GAGTAAGCATTACACAATCTCCAAGATGATTTTTTGGAAAAAAAAAGAGAATAGATCTTCCATTTTTCTATCACATATCCTATTTTG
ACACCACCACCACGAAATCAGGTTTTTTTTCATGAATTGTCACAAATTCATTTGTTTTTCATTATCAAGAACTTCTTTCATATCCAAA
TTCGATATTGTGGGAGACCCTAATAGGGTTAGGGTCTTTCACTGGAAAGGGGGTCAAAAATGAGGACATGGGGGTAAGCCGGATT
TATGGCGACTATCCAAGAATTTCAGTGTGCTAATCTAGGATTCATACTCTAAAACTTATCTGATTTTCTCAATTGTTAGGATTTTTCT
CGAAGAAATCATGCATTTTCTAAGATATTATTATTAAGGATCTCATCGAAAACTTACAGCAGCTTGCCAAACA
>*Leucophyllum laevigatum* 1. (psbK) L_laevigatumBon1
GAGTAAGCATTACACAATCTCCAAGATGATTTTTTGGAAAAAAAAAA-
GAGAATAGATCTTCCATTTTTCTATCACATATCCTATTTTGACACCACCACCACGAAATCAGGTTTTTTTTCATGAATTGTCACAAA
TTCATTTGTTTTTCATTATCAAGAACTTCTTTCATATCCAAATTCGATATTGTGGGAGACCCTAATAGGGTTAGGGTCTTTCACTGGA
AAGGGGGTCAAAAATGAGGACATGGGGGTAAGCCGGATTTATGGCGACTATACAAGAATTTCAGTGTGCTAATCTAGGATTCATA
CTCTAAAACTTATCTGATTTTCTCAATTGTTAGGATTTTTCTCGAAGAAATCATGCATTTTCTAAGATATTATTATTAAGGATCTCAT
CGAAAACTTACAGCAGCTTGCCAAACA
>*Leucophyllum laevigatum* 4. (psbK) L_laevigatumBon4
GAGTAAGCATTACACAATCTCCAAGATGATTTTTTGGAAAAAAAAAGAGAATAGATCTTCCATTTTTCTATCACATATCCTATTTTG
ACACCACCACCACGAAATCAGGTTTTTTTTTCATGAATTGTCACAAATTCATTTGTTTTTCATTATCAAGAACTTCTTTCATATCCAA
ATTCGATATTGTGGGAGACCCTAATAGGGTTAGGGTCTTTCACTGGAAAGGGGGTCAAAAATGAGGACATGGGGGTAAGCCGGAT
TTATGGCGACTATCCAAGAATTTCAGTGTGCTAATCTAGGATTCATACTCTAAAACTTATCTGATTTTCTCAATTGTTAGGATTTTTC
TCGAAGAAATCATGCATTTTCTAAGATATTATTATTAAGGATCTCATCGAAAACTTACAGCAGCTTGCCAAACA
