# Supplementary material for: Climate change and conservation in a warm North American desert: effect in shrubby plants
Source: PeerJ. 2019 Mar 7;7:e6572. doi: 10.7717/peerj.6572 (PMC6409089; doi:10.7717/peerj.6572)
Supplement: Supplemental Information 2 [file peerj-07-6572-s002.pdf]

Table S2. Georeferences of the five shrubby species considered in this study, used for ecological niche modeling.

**Especie**	**Longitude**	**Latitude**
Berberis_trifoliolata	-99.227902	20.6907301
Berberis_trifoliolata	-100.6679	22.1307301
Berberis_trifoliolata	-100.9879	22.1307301
Berberis_trifoliolata	-100.8279	22.4507301
Berberis_trifoliolata	-100.5079	22.6107301
Berberis_trifoliolata	-101.6279	22.6107301
Berberis_trifoliolata	-100.3479	23.0907301
Berberis_trifoliolata	-100.9879	23.2507301
Berberis_trifoliolata	-99.547902	23.2507301
Berberis_trifoliolata	-99.867902	23.5707301
Berberis_trifoliolata	-100.8279	23.7307301
Berberis_trifoliolata	-100.0279	24.5307301
Berberis_trifoliolata	-105.4679	24.5307301
Berberis_trifoliolata	-100.9879	24.6907301
Berberis_trifoliolata	-99.867902	24.6907301
Berberis_trifoliolata	-100.0279	24.8507301
Berberis_trifoliolata	-100.1879	24.8507301
Berberis_trifoliolata	-100.6679	25.1707301
Berberis_trifoliolata	-100.9879	25.1707301
Berberis_trifoliolata	-100.8279	25.3307301
Berberis_trifoliolata	-100.9879	25.3307301
Berberis_trifoliolata	-101.9479	25.3307301
Berberis_trifoliolata	-102.1079	25.3307301
Berberis_trifoliolata	-101.1479	25.4907301
Berberis_trifoliolata	-100.8279	25.6507301
Berberis_trifoliolata	-103.7079	25.8107301
Berberis_trifoliolata	-104.5079	25.8107301
Berberis_trifoliolata	-105.3079	25.9707301
Berberis_trifoliolata	-100.6679	26.1307301
Berberis_trifoliolata	-101.3079	26.2907301
Berberis_trifoliolata	-103.8679	26.2907301
Berberis_trifoliolata	-102.2679	27.2507301
Berberis_trifoliolata	-102.5879	27.2507301
Berberis_trifoliolata	-105.7879	27.2507301
Berberis_trifoliolata	-98.907902	27.2507301
Berberis_trifoliolata	-102.5879	27.8907301
Berberis_trifoliolata	-102.1079	28.2107301
Berberis_trifoliolata	-96.667902	28.5307301

Berberis_trifoliolata	-104.1879	28.8507301
Berberis_trifoliolata	-105.6279	29.0107301
Berberis_trifoliolata	-105.4679	29.1707301
Berberis_trifoliolata	-101.9479	29.3307301
Berberis_trifoliolata	-103.3879	29.6507301
Berberis_trifoliolata	-101.6279	29.8107301
Berberis_trifoliolata	-97.947902	30.1307301
Berberis_trifoliolata	-98.267902	30.4507301
Berberis_trifoliolata	-110.5879	32.0507301
Berberis_trifoliolata	-109.7879	34.2907301
Ephedra_aspera	-103.8679	20.8507301
Ephedra_aspera	-100.1879	22.2907301
Ephedra_aspera	-100.8279	22.2907301
Ephedra_aspera	-100.5079	22.4507301
Ephedra_aspera	-101.4679	22.4507301
Ephedra_aspera	-102.9079	23.2507301
Ephedra_aspera	-99.707902	23.2507301
Ephedra_aspera	-103.0679	23.5707301
Ephedra_aspera	-100.8279	23.7307301
Ephedra_aspera	-100.0279	23.8907301
Ephedra_aspera	-99.867902	23.8907301
Ephedra_aspera	-104.0279	24.0507301
Ephedra_aspera	-100.1879	24.2107301
Ephedra_aspera	-102.1079	24.3707301
Ephedra_aspera	-111.7079	24.3707301
Ephedra_aspera	-101.1479	24.6907301
Ephedra_aspera	-101.4679	24.6907301
Ephedra_aspera	-112.1879	24.6907301
Ephedra_aspera	-100.0279	24.8507301
Ephedra_aspera	-100.3479	24.8507301
Ephedra_aspera	-101.1479	24.8507301
Ephedra_aspera	-101.6279	24.8507301
Ephedra_aspera	-101.7879	24.8507301
Ephedra_aspera	-100.5079	25.0107301
Ephedra_aspera	-100.9879	25.0107301
Ephedra_aspera	-101.3079	25.0107301
Ephedra_aspera	-103.0679	25.0107301
Ephedra_aspera	-100.5079	25.1707301
Ephedra_aspera	-102.4279	25.1707301
Ephedra_aspera	-102.1079	25.3307301
Ephedra_aspera	-102.2679	25.3307301

Ephedra_aspera	-102.7479	25.3307301
Ephedra_aspera	-100.9879	25.4907301
Ephedra_aspera	-101.1479	25.4907301
Ephedra_aspera	-102.1079	25.4907301
Ephedra_aspera	-102.4279	25.4907301
Ephedra_aspera	-103.2279	25.4907301
Ephedra_aspera	-111.2279	26.1307301
Ephedra_aspera	-99.227902	26.9307301
Ephedra_aspera	-104.8279	27.5707301
Ephedra_aspera	-114.5879	27.5707301
Ephedra_aspera	-114.9079	27.7307301
Ephedra_aspera	-115.2279	28.0507301
Ephedra_aspera	-102.9079	28.2107301
Ephedra_aspera	-113.4679	28.3707301
Ephedra_aspera	-105.9479	28.5307301
Ephedra_aspera	-114.1079	28.5307301
Ephedra_aspera	-112.5079	28.6907301
Ephedra_aspera	-102.5879	29.1707301
Ephedra_aspera	-100.8279	29.3307301
Ephedra_aspera	-103.8679	29.3307301
Ephedra_aspera	-104.6679	29.3307301
Ephedra_aspera	-105.3079	29.3307301
Ephedra_aspera	-114.5879	29.6507301
Ephedra_aspera	-114.7479	29.8107301
Ephedra_aspera	-115.0679	30.1307301
Ephedra_aspera	-115.5479	30.1307301
Ephedra_aspera	-112.3479	30.2907301
Ephedra_aspera	-113.1479	31.0907301
Ephedra_aspera	-108.3479	31.7307301
Ephedra_aspera	-113.4679	31.7307301
Ephedra_aspera	-106.1079	31.8907301
Ephedra_aspera	-106.4279	31.8907301
Ephedra_aspera	-107.0679	31.8907301
Ephedra_aspera	-112.9879	31.8907301
Ephedra_aspera	-113.4679	31.8907301
Ephedra_aspera	-104.5079	32.0507301
Ephedra_aspera	-105.4679	32.0507301
Ephedra_aspera	-113.6279	32.0507301
Ephedra_aspera	-113.7879	32.0507301
Ephedra_aspera	-114.1079	32.0507301
Ephedra_aspera	-114.2679	32.0507301

Ephedra_aspera	-104.3479	32.2107301
Ephedra_aspera	-104.5079	32.2107301
Ephedra_aspera	-104.6679	32.2107301
Ephedra_aspera	-106.5879	32.2107301
Ephedra_aspera	-110.9079	32.2107301
Ephedra_aspera	-112.6679	32.2107301
Ephedra_aspera	-113.7879	32.2107301
Ephedra_aspera	-104.5079	32.3707301
Ephedra_aspera	-105.9479	32.3707301
Ephedra_aspera	-106.7479	32.3707301
Ephedra_aspera	-108.6679	32.3707301
Ephedra_aspera	-110.7479	32.3707301
Ephedra_aspera	-110.9079	32.3707301
Ephedra_aspera	-111.0679	32.3707301
Ephedra_aspera	-111.3879	32.3707301
Ephedra_aspera	-111.5479	32.3707301
Ephedra_aspera	-112.8279	32.3707301
Ephedra_aspera	-112.9879	32.3707301
Ephedra_aspera	-104.3479	32.5307301
Ephedra_aspera	-104.5079	32.5307301
Ephedra_aspera	-106.1079	32.5307301
Ephedra_aspera	-106.4279	32.5307301
Ephedra_aspera	-110.5879	32.5307301
Ephedra_aspera	-111.5479	32.5307301
Ephedra_aspera	-112.6679	32.5307301
Ephedra_aspera	-113.3079	32.5307301
Ephedra_aspera	-116.0279	32.5307301
Ephedra_aspera	-109.7879	32.6907301
Ephedra_aspera	-109.9479	32.6907301
Ephedra_aspera	-111.0679	32.6907301
Ephedra_aspera	-111.2279	32.6907301
Ephedra_aspera	-111.5479	32.6907301
Ephedra_aspera	-112.1879	32.6907301
Ephedra_aspera	-112.3479	32.6907301
Ephedra_aspera	-112.5079	32.6907301
Ephedra_aspera	-112.6679	32.6907301
Ephedra_aspera	-109.4679	32.8507301
Ephedra_aspera	-109.6279	32.8507301
Ephedra_aspera	-112.1879	32.8507301
Ephedra_aspera	-112.3479	32.8507301
Ephedra_aspera	-112.5079	32.8507301

Ephedra_aspera	-114.7479	32.8507301
Ephedra_aspera	-109.6279	33.0107301
Ephedra_aspera	-109.9479	33.0107301
Ephedra_aspera	-111.3879	33.0107301
Ephedra_aspera	-111.7079	33.0107301
Ephedra_aspera	-113.4679	33.0107301
Ephedra_aspera	-114.1079	33.0107301
Ephedra_aspera	-115.0679	33.0107301
Ephedra_aspera	-116.0279	33.0107301
Ephedra_aspera	-116.5079	33.0107301
Ephedra_aspera	-116.9879	33.0107301
Ephedra_aspera	-111.7079	33.1707301
Ephedra_aspera	-112.1879	33.1707301
Ephedra_aspera	-106.5879	33.3307301
Ephedra_aspera	-110.4279	33.3307301
Ephedra_aspera	-111.7079	33.3307301
Ephedra_aspera	-112.3479	33.3307301
Ephedra_aspera	-113.7879	33.3307301
Ephedra_aspera	-113.9479	33.3307301
Ephedra_aspera	-114.1079	33.3307301
Ephedra_aspera	-114.9079	33.3307301
Ephedra_aspera	-111.3879	33.4907301
Ephedra_aspera	-111.5479	33.4907301
Ephedra_aspera	-111.8679	33.4907301
Ephedra_aspera	-113.3079	33.4907301
Ephedra_aspera	-114.1079	33.4907301
Ephedra_aspera	-111.0679	33.6507301
Ephedra_aspera	-111.2279	33.6507301
Ephedra_aspera	-112.9879	33.6507301
Ephedra_aspera	-115.7079	33.6507301
Ephedra_aspera	-105.9479	33.8107301
Ephedra_aspera	-111.8679	33.8107301
Ephedra_aspera	-113.3079	33.8107301
Ephedra_aspera	-113.6279	33.8107301
Ephedra_aspera	-116.5079	33.8107301
Ephedra_aspera	-112.3479	33.9707301
Ephedra_aspera	-112.9879	33.9707301
Ephedra_aspera	-113.4679	33.9707301
Ephedra_aspera	-113.9479	33.9707301
Ephedra_aspera	-114.1079	33.9707301
Ephedra_aspera	-115.0679	33.9707301

Ephedra_aspera	-116.0279	33.9707301
Ephedra_aspera	-111.0679	34.1307301
Ephedra_aspera	-114.1079	34.2907301
Ephedra_aspera	-114.4279	34.2907301
Ephedra_aspera	-114.5879	34.2907301
Ephedra_aspera	-112.3479	34.4507301
Ephedra_aspera	-114.5879	34.6107301
Ephedra_aspera	-114.1079	34.7707301
Ephedra_aspera	-113.9479	34.9307301
Ephedra_aspera	-116.0279	34.9307301
Ephedra_aspera	-117.9479	34.9307301
Ephedra_aspera	-113.9479	35.0907301
Ephedra_aspera	-114.1079	35.0907301
Ephedra_aspera	-114.2679	35.0907301
Ephedra_aspera	-114.4279	35.0907301
Ephedra_aspera	-114.1079	35.2507301
Ephedra_aspera	-114.7479	35.2507301
Ephedra_aspera	-114.4279	35.4107301
Ephedra_aspera	-114.5879	35.4107301
Ephedra_aspera	-113.3079	35.7307301
Ephedra_aspera	-114.5879	35.8907301
Ephedra_aspera	-112.0279	36.0507301
Ephedra_aspera	-112.1879	36.0507301
Ephedra_aspera	-113.9479	36.0507301
Ephedra_aspera	-112.0279	36.2107301
Ephedra_aspera	-112.3479	36.2107301
Ephedra_aspera	-112.6679	36.2107301
Ephedra_aspera	-113.1479	36.2107301
Ephedra_aspera	-113.7879	36.2107301
Ephedra_aspera	-113.9479	36.2107301
Ephedra_aspera	-112.3479	36.3707301
Ephedra_aspera	-114.2679	36.5307301
Ephedra_aspera	-113.7879	37.0107301
Ephedra_aspera	-113.9479	37.0107301
Ephedra_aspera	-113.4679	37.1707301
Ephedra_aspera	-113.6279	37.1707301
Ephedra_aspera	-113.7879	37.1707301
Ephedra_aspera	-117.7879	37.1707301
Ephedra_aspera	-118.1079	37.9707301
Leucophyllum_laevigatum	-102.7479	23.4107301
Leucophyllum_laevigatum	-102.1079	23.5707301

Leucophyllum_laevigatum	-101.7879	23.7307301
Leucophyllum_laevigatum	-101.9479	23.7307301
Leucophyllum_laevigatum	-102.9079	23.7307301
Leucophyllum_laevigatum	-103.2279	23.7307301
Leucophyllum_laevigatum	-103.8679	23.7307301
Leucophyllum_laevigatum	-101.4679	23.8907301
Leucophyllum_laevigatum	-103.0679	23.8907301
Leucophyllum_laevigatum	-103.2279	23.8907301
Leucophyllum_laevigatum	-101.4679	24.0507301
Leucophyllum_laevigatum	-102.7479	24.0507301
Leucophyllum_laevigatum	-104.6679	24.0507301
Leucophyllum_laevigatum	-101.3079	24.2107301
Leucophyllum_laevigatum	-100.1879	24.5307301
Leucophyllum_laevigatum	-101.1479	24.5307301
Leucophyllum_laevigatum	-101.4679	24.5307301
Leucophyllum_laevigatum	-102.2679	24.5307301
Leucophyllum_laevigatum	-102.7479	24.5307301
Leucophyllum_laevigatum	-103.3879	24.5307301
Leucophyllum_laevigatum	-100.8279	24.6907301
Leucophyllum_laevigatum	-101.1479	24.6907301
Leucophyllum_laevigatum	-101.3079	24.6907301
Leucophyllum_laevigatum	-101.4679	24.6907301
Leucophyllum_laevigatum	-101.6279	24.6907301
Leucophyllum_laevigatum	-102.7479	24.6907301
Leucophyllum_laevigatum	-103.7079	24.6907301
Leucophyllum_laevigatum	-100.1879	24.8507301
Leucophyllum_laevigatum	-101.6279	24.8507301
Leucophyllum_laevigatum	-102.2679	24.8507301
Leucophyllum_laevigatum	-103.7079	24.8507301
Leucophyllum_laevigatum	-104.5079	24.8507301
Leucophyllum_laevigatum	-102.5879	25.0107301
Leucophyllum_laevigatum	-103.5479	25.0107301
Leucophyllum_laevigatum	-103.7079	25.0107301
Leucophyllum_laevigatum	-103.8679	25.0107301
Leucophyllum_laevigatum	-104.6679	25.0107301
Leucophyllum_laevigatum	-105.3079	25.0107301
Leucophyllum_laevigatum	-103.2279	25.1707301
Leucophyllum_laevigatum	-103.3879	25.1707301
Leucophyllum_laevigatum	-103.7079	25.1707301
Leucophyllum_laevigatum	-103.8679	25.1707301
Leucophyllum_laevigatum	-104.5079	25.1707301

Leucophyllum_laevigatum	-104.6679	25.1707301
Leucophyllum_laevigatum	-105.6279	25.1707301
Leucophyllum_laevigatum	-102.1079	25.3307301
Leucophyllum_laevigatum	-102.2679	25.3307301
Leucophyllum_laevigatum	-104.5079	25.3307301
Leucophyllum_laevigatum	-104.6679	25.3307301
Leucophyllum_laevigatum	-105.6279	25.3307301
Leucophyllum_laevigatum	-102.4279	25.4907301
Leucophyllum_laevigatum	-104.0279	25.4907301
Leucophyllum_laevigatum	-104.6679	25.4907301
Leucophyllum_laevigatum	-104.0279	25.6507301
Leucophyllum_laevigatum	-103.8679	25.8107301
Leucophyllum_laevigatum	-104.0279	25.8107301
Leucophyllum_laevigatum	-104.5079	25.8107301
Leucophyllum_laevigatum	-104.6679	25.8107301
Leucophyllum_laevigatum	-101.1479	25.9707301
Leucophyllum_laevigatum	-105.4679	25.9707301
Leucophyllum_laevigatum	-102.9079	26.6107301
Leucophyllum_laevigatum	-104.0279	26.6107301
Leucophyllum_laevigatum	-102.1079	26.7707301
Leucophyllum_laevigatum	-104.3479	26.7707301
Leucophyllum_laevigatum	-104.0279	27.0907301
Leucophyllum_laevigatum	-104.6679	27.0907301
Leucophyllum_laevigatum	-104.9879	27.0907301
Leucophyllum_laevigatum	-104.8279	27.2507301
Leucophyllum_laevigatum	-104.9879	27.2507301
Leucophyllum_laevigatum	-105.6279	27.2507301
Leucophyllum_laevigatum	-105.7879	27.2507301
Leucophyllum_laevigatum	-104.9879	27.5707301
Leucophyllum_laevigatum	-102.9079	28.0507301
Leucophyllum_laevigatum	-102.9079	28.2107301
Leucophyllum_laevigatum	-103.2279	28.2107301
Leucophyllum_laevigatum	-103.5479	28.2107301
Lindleya_mespiloides	-97.947902	17.4907301
Lindleya_mespiloides	-97.307902	17.6507301
Lindleya_mespiloides	-97.467902	17.6507301
Lindleya_mespiloides	-97.787902	17.6507301
Lindleya_mespiloides	-97.147902	17.8107301
Lindleya_mespiloides	-97.467902	17.8107301
Lindleya_mespiloides	-97.627902	17.8107301
Lindleya_mespiloides	-97.787902	17.8107301

Lindleya_mespiloides	-97.627902	17.9707301
Lindleya_mespiloides	-97.467902	18.2907301
Lindleya_mespiloides	-97.627902	18.2907301
Lindleya_mespiloides	-97.307902	18.6107301
Lindleya_mespiloides	-98.587902	20.3707301
Lindleya_mespiloides	-99.067902	20.3707301
Lindleya_mespiloides	-98.747902	20.5307301
Lindleya_mespiloides	-99.067902	20.6907301
Lindleya_mespiloides	-99.387902	20.6907301
Lindleya_mespiloides	-99.227902	20.8507301
Lindleya_mespiloides	-99.707902	20.8507301
Lindleya_mespiloides	-99.867902	20.8507301
Lindleya_mespiloides	-99.547902	21.0107301
Lindleya_mespiloides	-99.867902	21.0107301
Lindleya_mespiloides	-99.227902	21.1707301
Lindleya_mespiloides	-99.387902	21.1707301
Lindleya_mespiloides	-99.067902	21.3307301
Lindleya_mespiloides	-99.227902	21.3307301
Lindleya_mespiloides	-100.8279	22.4507301
Lindleya_mespiloides	-101.1479	22.4507301
Lindleya_mespiloides	-100.3479	22.6107301
Lindleya_mespiloides	-100.5079	22.6107301
Lindleya_mespiloides	-99.227902	23.2507301
Lindleya_mespiloides	-99.707902	23.2507301
Lindleya_mespiloides	-99.867902	23.2507301
Lindleya_mespiloides	-99.707902	23.4107301
Lindleya_mespiloides	-100.5079	23.5707301
Lindleya_mespiloides	-99.707902	23.5707301
Lindleya_mespiloides	-99.867902	23.5707301
Lindleya_mespiloides	-100.1879	23.7307301
Lindleya_mespiloides	-100.8279	23.7307301
Lindleya_mespiloides	-100.9879	23.7307301
Lindleya_mespiloides	-100.0279	23.8907301
Lindleya_mespiloides	-100.0279	24.5307301
Lindleya_mespiloides	-100.6679	24.5307301
Lindleya_mespiloides	-100.0279	24.6907301
Lindleya_mespiloides	-100.1879	24.6907301
Lindleya_mespiloides	-100.3479	24.6907301
Lindleya_mespiloides	-101.1479	24.6907301
Lindleya_mespiloides	-101.4679	24.6907301
Lindleya_mespiloides	-99.867902	24.6907301

Lindleya_mespiloides	-100.0279	24.8507301
Lindleya_mespiloides	-100.1879	24.8507301
Lindleya_mespiloides	-100.3479	24.8507301
Lindleya_mespiloides	-100.0279	25.0107301
Lindleya_mespiloides	-100.3479	25.0107301
Lindleya_mespiloides	-100.5079	25.0107301
Lindleya_mespiloides	-101.1479	25.1707301
Lindleya_mespiloides	-100.3479	25.3307301
Lindleya_mespiloides	-100.8279	25.3307301
Lindleya_mespiloides	-101.7879	25.3307301
Lindleya_mespiloides	-100.5079	25.4907301
Lindleya_mespiloides	-100.6679	25.4907301
Lindleya_mespiloides	-100.8279	25.4907301
Lindleya_mespiloides	-100.9879	25.4907301
Lindleya_mespiloides	-101.4679	25.4907301
Lindleya_mespiloides	-100.3479	25.6507301
Lindleya_mespiloides	-102.7479	26.2907301
Lindleya_mespiloides	-102.9079	26.2907301
Lindleya_mespiloides	-101.9479	26.6107301
Lindleya_mespiloides	-102.5879	26.7707301
Lindleya_mespiloides	-103.0679	26.7707301
Lindleya_mespiloides	-102.4279	27.0907301
Lindleya_mespiloides	-102.5879	27.0907301
Lindleya_mespiloides	-103.7079	27.2507301
Lindleya_mespiloides	-104.6679	27.2507301
Lindleya_mespiloides	-104.6679	27.4107301
Lindleya_mespiloides	-103.8679	27.5707301
Lindleya_mespiloides	-102.9079	27.8907301
Lindleya_mespiloides	-103.7079	27.8907301
Setchellanthus caeruleus	-96.987902	17.8107301
Setchellanthus caeruleus	-96.987902	18.1307301
Setchellanthus caeruleus	-97.147902	18.1307301
Setchellanthus caeruleus	-97.467902	18.2907301
Setchellanthus caeruleus	-103.2279	25.1707301
Setchellanthus caeruleus	-103.7079	25.1707301
Setchellanthus caeruleus	-103.3879	25.3307301
Setchellanthus caeruleus	-103.7079	25.4907301
Setchellanthus caeruleus	-103.8679	25.6507301
