# Supplementary material for: Climate change and conservation in a warm North American desert: effect in shrubby plants
Source: PeerJ. 2019 Mar 7;7:e6572. doi: 10.7717/peerj.6572 (PMC6409089; doi:10.7717/peerj.6572)
Supplement: Supplemental Information 3 [file peerj-07-6572-s003.docx]

**Table S3. Fst estimations for every studied species. Population numbers correspond to localities included in Table 1.**

**Population pairwise F**_ST_ **for *Berberis trifoliolata***

| 1 2 3 4 5 6 7 8 9 10 11 12 13 14 15 16 17 18 19 20 | | | | | | | | | |  |  |  |  |  |  |  |
| --- | --- | --- | --- | --- | --- | --- | --- | --- | --- | --- | --- | --- | --- | --- | --- | --- |
| 1 0.00000 |  |  |  |  |  |  |  |  |  |  |  |  |  |  |  |  |
| 2 0.55556 0.00000 | |  |  |  |  |  |  |  |  |  |  |  |  |  |  |  |
| 3 0.63636 1.00000 0.00000 | | |  |  |  |  |  |  |  |  |  |  |  |  |  |  |
| 4 0.71461 0.77778 0.81818 0.00000 | | | |  |  |  |  |  |  |  |  |  |  |  |  |  |
| 5 0.68103 -0.08333 0.40909 0.55224 0.00000 | | | | |  |  |  |  |  |  |  |  |  |  |  |  |
| 6 0.66197 1.00000 1.00000 0.84615 0.51852 0.00000 | | | | |  |  |  |  |  |  |  |  |  |  |  |  |
| 7 0.57895 1.00000 1.00000 0.92593 0.80597 1.00000 0.00000 | | | | |  |  |  |  |  |  |  |  |  |  |  |  |
| 8 0.66197 1.00000 1.00000 0.86667 0.51852 1.00000 1.00000 0.00000 | | | | | |  |  |  |  |  |  |  |  |  |  |  |
| 9 0.60656 1.00000 1.00000 0.81818 0.23529 1.00000 1.00000 1.00000 0.00000 | | | | | | |  |  |  |  |  |  |  |  |  |  |
| 10 0.49625 -0.63636 -0.50000 0.37500 0.21330 -0.20000 0.41935 -0.20000 -0.63636 0.00000 | | | | | | | |  |  |  |  |  |  |  |  |  |
| 11 0.63636 1.00000 1.00000 0.71429 0.40909 1.00000 1.00000 1.00000 1.00000 -0.20000 0.00000 | | | | | | | | |  |  |  |  |  |  |  |  |
| 12 0.65624 0.14286 0.14286 0.66667 0.30426 0.33333 0.79310 0.33333 -0.20000 0.00000 0.45455 0.00000 | | | | | | | | | |  |  |  |  |  |  |  |
| 13 0.65624 0.14286 0.45455 0.50000 0.26616 0.53846 0.76000 0.53846 0.33333 0.07692 0.14286 0.40000 0.00000 | | | | | | | | | |  |  |  |  |  |  |  |
| 14 0.60656 1.00000 1.00000 0.71429 0.23529 1.00000 1.00000 1.00000 1.00000 -0.38462 1.00000 0.33333 -0.20000 0.00000 | | | | | | | | | | |  |  |  |  |  |  |
| 15 0.70066 0.50000 0.71429 0.61628 0.31500 0.76471 0.87879 0.76471 0.63636 0.32258 0.50000 0.53636 0.08929 0.20000 0.00000 | | | | | | | | | | | |  |  |  |  |  |
| 16 0.70631 0.60000 0.84615 0.85714 0.55224 0.86667 0.92593 0.86667 0.81818 0.37500 0.84615 0.66667 0.66667 0.81818 0.72951 0.00000 | | | | | | | | | | | | |  |  |  |  |
| 17 0.60656 1.00000 1.00000 0.81818 0.23529 1.00000 1.00000 1.00000 1.00000 -0.63636 1.00000 -0.20000 0.33333 1.00000 0.63636 0.81818 0.00000 | | | | | | | | | | | | | |  |  |  |
| 18 0.70000 0.30769 0.47059 0.68437 0.26667 0.59091 0.85484 0.59091 0.25000 0.23513 0.60870 0.25980 0.48433 0.50000 0.43454 0.58959 0.25000 0.00000 | | | | | | | | | | | | | | |  |  |
| 19 0.64525 -1.00000 0.33333 0.63636 0.30426 0.53846 0.77778 0.53846 0.33333 0.14286 0.45455 0.40000 0.40000 0.33333 0.49505 0.20000 0.33333 0.39213 0.00000 | | | | | | | | | | | | | | | |  |
| 20 0.67241 0.30000 0.22222 0.57313 0.38636 0.57576 0.80822 0.57576 0.39130 0.27286 0.53333 0.44050 0.48137 0.44000 0.51822 0.63669 0.39130 0.40722 0.36550 0.00000 | | | | | | | | | | | | | | | |  |

**Population pairwise F_ST_ for *Ephedra aspera***

| 1 2 3 4 5 6 7 8 9 | | | | |  |  |  |
| --- | --- | --- | --- | --- | --- | --- | --- |
| 1 0.00000 |  |  |  |  |  |  |  |
| 2 0.92375 0.00000 | |  |  |  |  |  |  |
| 3 1.00000 0.96312 0.00000 | | |  |  |  |  |  |
| 4 1.00000 0.91474 1.00000 0.00000 | | | |  |  |  |  |
| 5 1.00000 0.61749 1.00000 1.00000 0.00000 | | | |  |  |  |  |
| 6 0.94801 0.92054 0.90228 0.91724 0.96945 0.00000 | | | | |  |  |  |
| 7 1.00000 0.94453 1.00000 1.00000 1.00000 0.36842 0.00000 | | | | | |  |  |
| 8 1.00000 0.87847 1.00000 1.00000 1.00000 0.86813 1.00000 0.00000 | | | | | | |  |
| 9 -0.00000 0.88628 0.95238 0.74359 0.93750 0.89159 0.93902 0.90148 0.00000 | | | | | | | |

**Population pairwise F_ST_ for *Leucophyllum laevigatum***

| 1 2 3 4 5 6 7 8 9 | | | | |  |  |  |
| --- | --- | --- | --- | --- | --- | --- | --- |
| 1 0.00000 |  |  |  |  |  |  |  |
| 2 1.00000 0.00000 | |  |  |  |  |  |  |
| 3 0.00000 1.00000 0.00000 | | |  |  |  |  |  |
| 4 0.30000 0.54472 0.46701 0.00000 | | | |  |  |  |  |
| 5 -0.13208 0.64126 0.00000 -0.03636 0.00000 | | | | |  |  |  |
| 6 1.00000 1.00000 1.00000 0.73717 0.75610 0.00000 | | | | |  |  |  |
| 7 0.90369 0.45205 0.92683 0.54919 0.64865 0.25000 0.00000 | | | | | |  |  |
| 8 0.21223 0.27812 0.32636 -0.14773 -0.00359 0.49434 0.32125 0.00000 | | | | | | |  |
| 9 0.89686 0.72028 0.92157 0.63885 0.66825 0.84615 0.68182 0.52269 0.00000 | | | | | | | |

**Population pairwise F_ST_ for *Lindleya mespiloides***

| 1 2 3 4 5 6 7 8 9 10 11 12 13 14 15 16 17 18 19 20 | | | | | | | | | | |  |  |  |  |  |  |  |  |
| --- | --- | --- | --- | --- | --- | --- | --- | --- | --- | --- | --- | --- | --- | --- | --- | --- | --- | --- |
| 1 0.00000 |  |  |  |  |  |  |  |  |  |  |  |  |  |  |  |  |  |  |
| 2 0.50098 0.00000 | |  |  |  |  |  |  |  |  |  |  |  |  |  |  |  |  |  |
| 3 0.56410 0.46504 0.00000 | | |  |  |  |  |  |  |  |  |  |  |  |  |  |  |  |  |
| 4 0.44710 0.03564 0.15196 0.00000 | | | |  |  |  |  |  |  |  |  |  |  |  |  |  |  |  |
| 5 0.72707 0.46643 0.58070 0.30644 0.00000 | | | |  |  |  |  |  |  |  |  |  |  |  |  |  |  |  |
| 6 0.63636 0.66584 0.55236 0.60000 0.79887 0.00000 | | | | |  |  |  |  |  |  |  |  |  |  |  |  |  |  |
| 7 0.59048 0.73827 0.68170 0.67237 0.86473 0.51803 0.00000 | | | | | |  |  |  |  |  |  |  |  |  |  |  |  |  |
| 8 0.75292 0.81886 0.83047 0.76835 0.92072 0.64556 0.21289 0.00000 | | | | | | |  |  |  |  |  |  |  |  |  |  |  |  |
| 9 0.56731 0.73219 0.70379 0.69083 0.84257 0.59297 0.03139 0.44629 0.00000 | | | | | | | |  |  |  |  |  |  |  |  |  |  |  |
| 10 0.53453 0.68955 0.62782 0.64904 0.79244 0.44768 0.10019 0.18824 0.27842 0.00000 | | | | | | | | |  |  |  |  |  |  |  |  |  |  |
| 11 0.75753 0.73837 0.72219 0.66894 0.88952 0.36293 0.75178 0.86767 0.66828 0.60875 0.00000 | | | | | | | | | |  |  |  |  |  |  |  |  |  |
| 12 0.62928 0.64519 0.56547 0.59921 0.78370 0.32584 0.55541 0.70235 0.55059 0.46943 0.15616 0.00000 | | | | | | | | | | |  |  |  |  |  |  |  |  |
| 13 0.66038 0.67812 0.57335 0.60840 0.81990 0.21664 0.50957 0.63517 0.59396 0.31759 0.64315 0.37789 0.00000 | | | | | | | | | | |  |  |  |  |  |  |  |  |
| 14 0.69772 0.69021 0.66748 0.63795 0.83511 0.56965 0.67217 0.79897 0.63209 0.49973 0.61669 0.21522 0.42717 0.00000 | | | | | | | | | | | |  |  |  |  |  |  |  |
| 15 0.57692 0.72956 0.64465 0.66655 0.83713 0.49863 0.26667 0.34762 0.44158 0.24033 0.74589 0.60559 0.47046 0.69542 0.00000 | | | | | | | | | | | | |  |  |  |  |  |  |
| 16 0.64603 0.77217 0.77276 0.70568 0.91212 0.69335 0.45110 0.75478 0.21594 0.34257 0.82353 0.56688 0.73981 0.71408 0.60866 0.00000 | | | | | | | | | | | | | |  |  |  |  |  |
| 17 0.69231 0.68534 0.63245 0.61302 0.85420 0.54000 0.65882 0.81999 0.59245 0.52654 0.63956 0.37622 0.57266 0.53506 0.66757 0.73608 0.00000 | | | | | | | | | | | | | | |  |  |  |  |
| 18 0.53973 0.68863 0.61290 0.60130 0.87127 0.53811 0.34004 0.71914 0.16676 0.24047 0.71429 0.37945 0.58650 0.56596 0.49661 0.05023 0.54766 0.00000 | | | | | | | | | | | | | | | |  |  |  |
| 19 0.48571 -0.01063 0.25587 -0.06472 0.59267 0.62423 0.74081 0.85355 0.72945 0.66393 0.76424 0.62105 0.65385 0.70032 0.70382 0.81411 0.69480 0.70448 0.00000 | | | | | | | | | | | | | | | | |  |  |
| 20 0.62611 0.45655 0.15393 0.20127 0.19142 0.69947 0.76576 0.84970 0.77842 0.72709 0.78775 0.69605 0.71009 0.74604 0.74718 0.81409 0.73773 0.73111 0.42768 0.00000 | | | | | | | | | | | | | | | | |  |  |

Population pairwise **F_ST_** for *Setchellanthus caeruleus*

| 1 2 3 4 | |  |
| --- | --- | --- |
| 1 0.00000 |  |  |
| 2 -0.19048 0.00000 | |  |
| 3 0.86250 0.88462 0.00000 | | |
| 4 0.89639 0.91988 0.41447 0.00000 | | |
