# Supplementary material for: Climate change and conservation in a warm North American desert: effect in shrubby plants
Source: PeerJ. 2019 Mar 7;7:e6572. doi: 10.7717/peerj.6572 (PMC6409089; doi:10.7717/peerj.6572)
Supplement: Supplemental Information 4 [file peerj-07-6572-s004.docx]

Table S4. Loadings of single bioclimatic variables for the first six PC axes.

| Variable | PC1 | PC2 | PC3 | PC4 | PC5 | PC6 |
| --- | --- | --- | --- | --- | --- | --- |
| Bio1 | 0.20201089 | 0.26465031 | -0.28965545 | 0.06503255 | -0.0025092 | 0.10825205 |
| Bio2 | -0.14640637 | -0.28198306 | -0.13260281 | -0.30494417 | 0.62519525 | 0.15988857 |
| Bio3 | 0.2519832 | -0.2869786 | -0.03095461 | 0.11026281 | 0.3265641 | 0.19817823 |
| Bio4 | -0.31808897 | 0.18225536 | -0.01988091 | -0.24472142 | -0.1247523 | -0.0906066 |
| Bio5 | -0.06048744 | 0.29455254 | -0.3248916 | -0.22473598 | 0.20785174 | 0.21959912 |
| Bio6 | 0.32144349 | 0.13442119 | -0.15403329 | 0.21661376 | -0.03049171 | 0.05191954 |
| Bio7 | -0.33293425 | 0.05588971 | -0.0563397 | -0.33658731 | 0.15487457 | 0.08616317 |
| Bio8 | 0.01628761 | 0.28879601 | -0.34880587 | -0.15822313 | -0.13095974 | -0.08603156 |
| Bio9 | 0.27879988 | 0.0763095 | -0.2100663 | 0.04255873 | 0.40089681 | -0.55923927 |
| Bio10 | -0.02692353 | 0.36041176 | -0.27770728 | -0.12354971 | -0.06798889 | 0.07559425 |
| Bio11 | 0.32115757 | 0.08997212 | -0.20324067 | 0.16262406 | 0.0800181 | 0.13335658 |
| Bio12 | 0.26459175 | 0.14807967 | 0.27234083 | -0.17764773 | 0.03347579 | 0.32573683 |
| Bio13 | 0.31277733 | 0.05559131 | 0.17314083 | -0.29631153 | -0.06999435 | 0.1587924 |
| Bio14 | -0.04622536 | 0.32848543 | 0.30066787 | 0.08540388 | 0.22418726 | 0.14242661 |
| Bio15 | 0.20709559 | -0.26289465 | -0.16788619 | -0.31431255 | -0.05438458 | -0.1899794 |
| Bio16 | 0.31544734 | 0.0036613 | 0.1717643 | -0.30686587 | -0.052888 | 0.21792337 |
| Bio17 | -0.0393534 | 0.33331795 | 0.30133842 | 0.10936319 | 0.23358306 | 0.01901623 |
| Bio18 | 0.24150578 | -0.0055594 | 0.17738162 | -0.46866267 | -0.17533626 | -0.32878294 |
| Bio19 | 0.03383038 | 0.29414419 | 0.32554576 | -0.01550902 | 0.28734406 | -0.41181738 |
