# Supplementary figures and images for: Climate change and conservation in a warm North American desert: effect in shrubby plants
Source: PeerJ. 2019 Mar 7;7:e6572. doi: 10.7717/peerj.6572 (PMC6409089; doi:10.7717/peerj.6572)

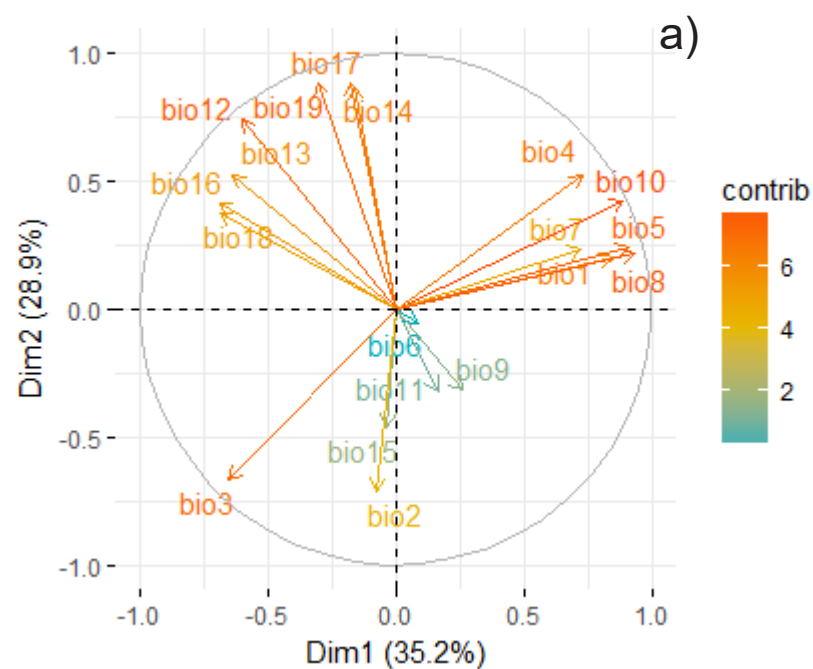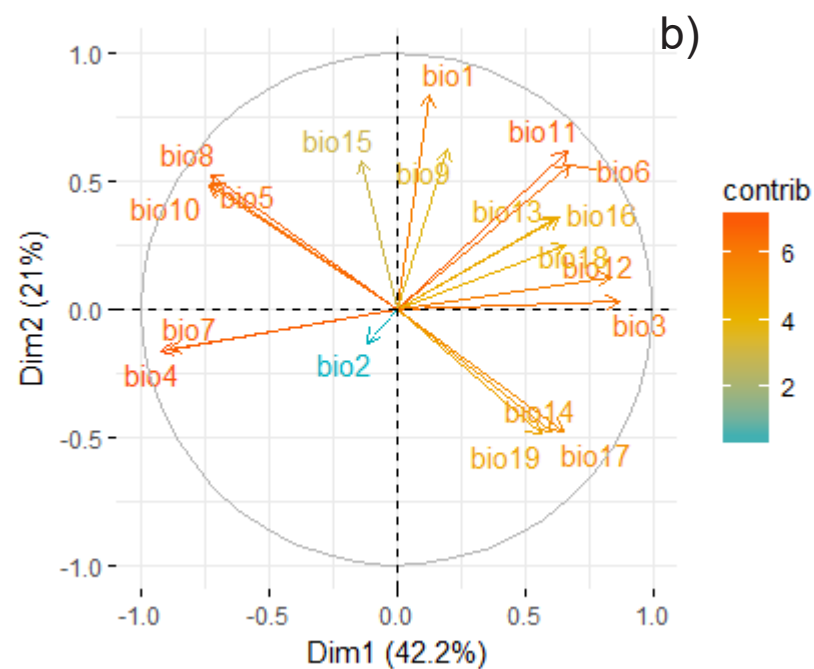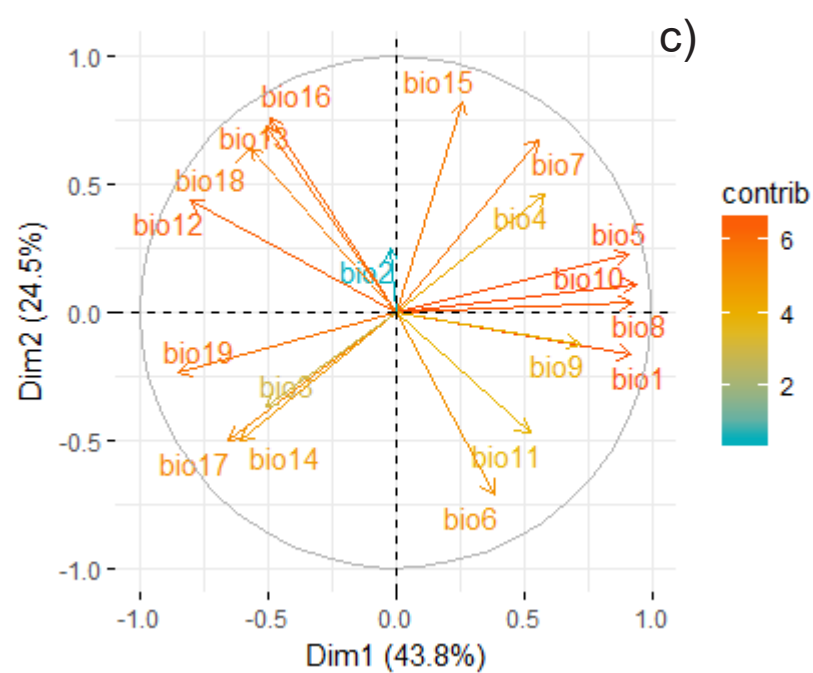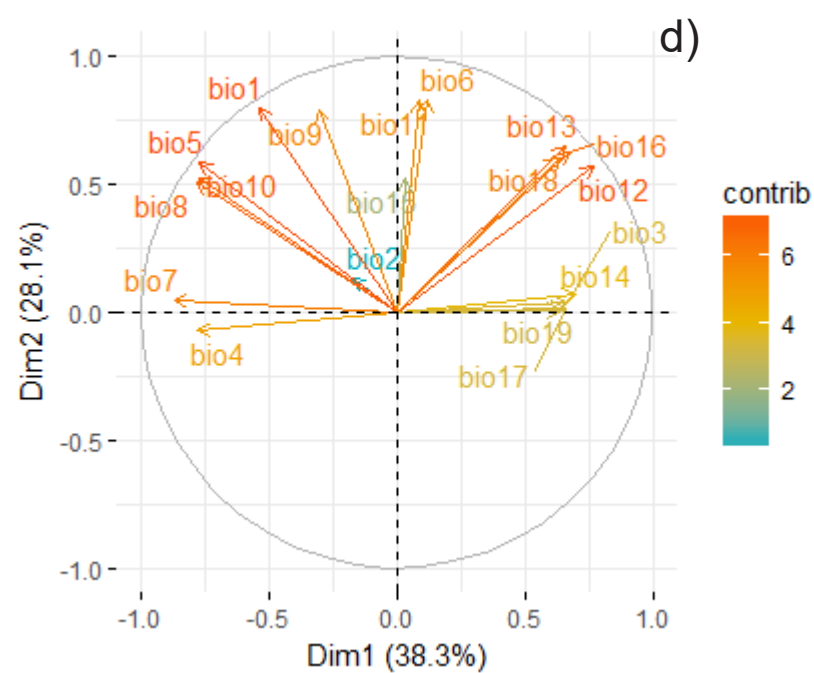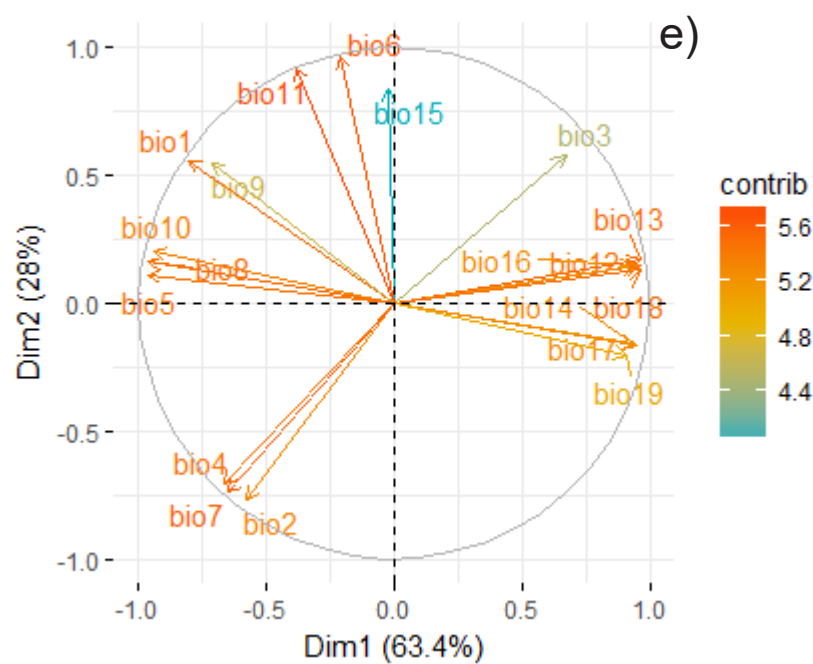

Supplement: Supplemental Information 5 — PCA graphs displaying contribution of climate variables for niche space for every species studied. a) Berberis trifoliolata. b) Ephedra compacta. c) Leucophyllum laevigatum. d) Lindleya mespiloides. d) Setchellanthus caeruleus. [file peerj-07-6572-s005.pdf]

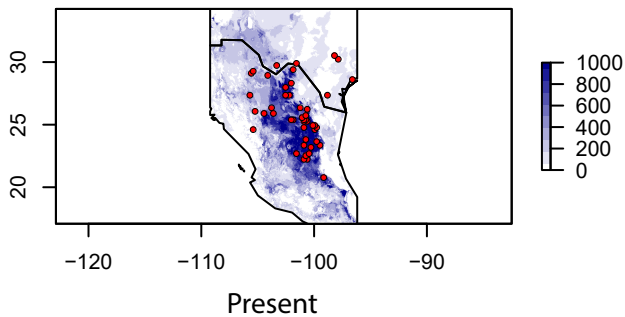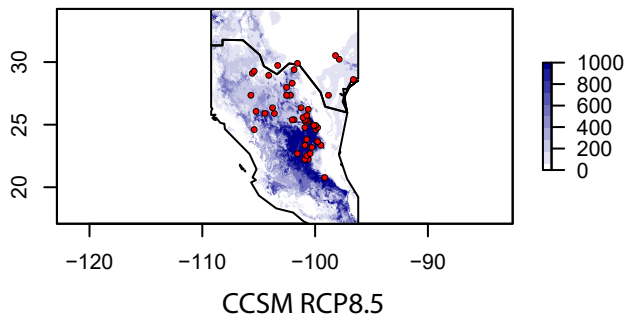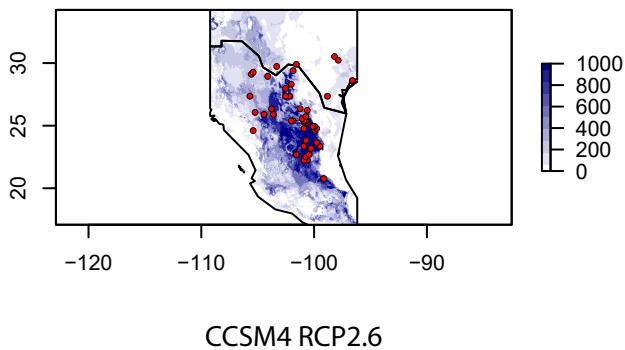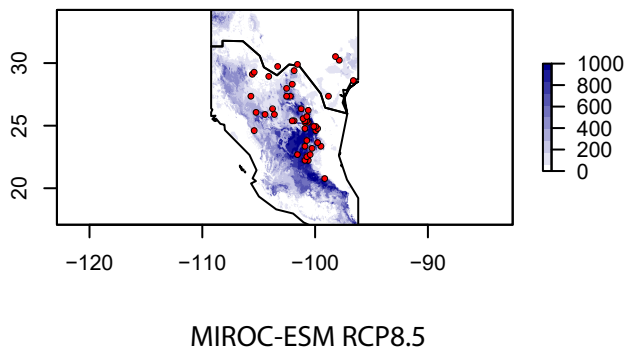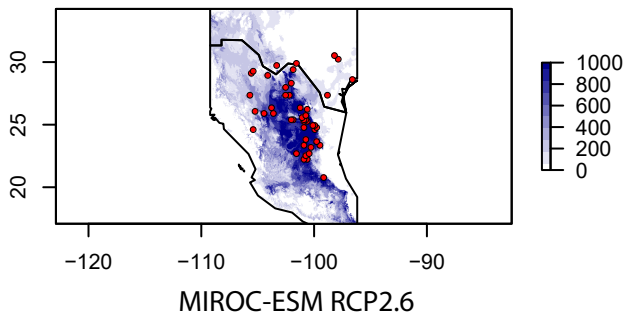

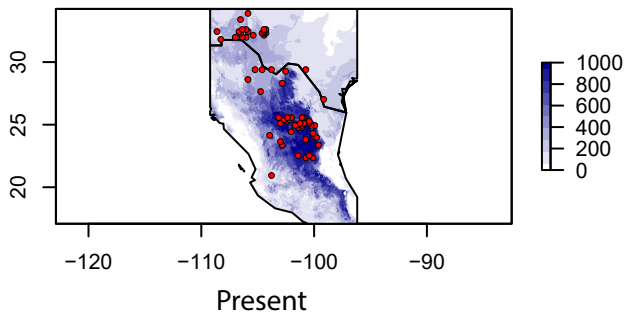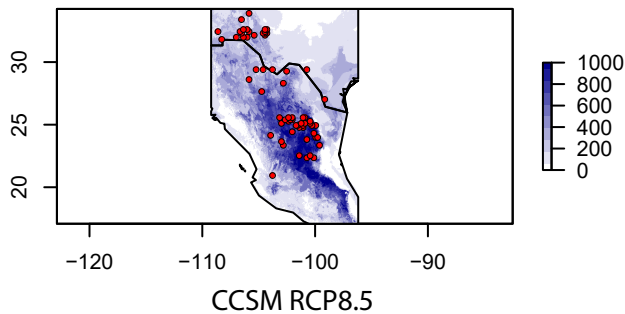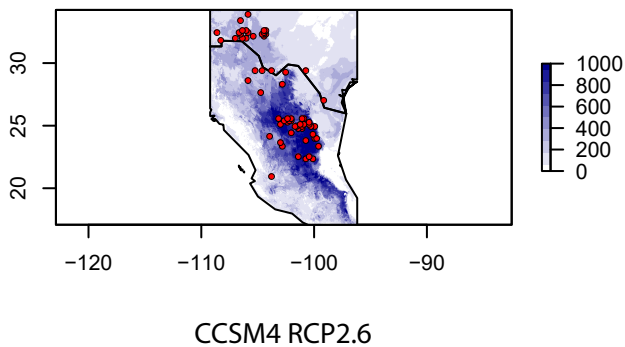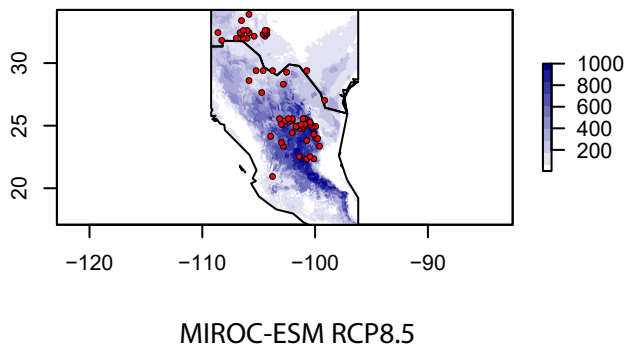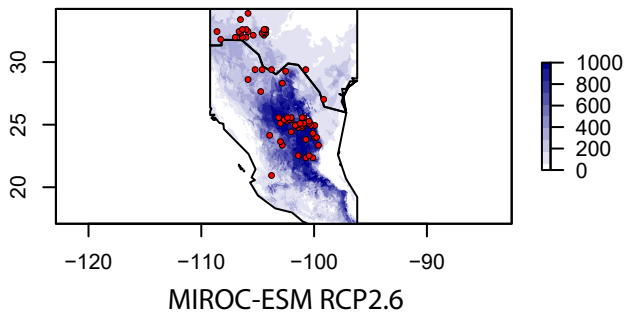

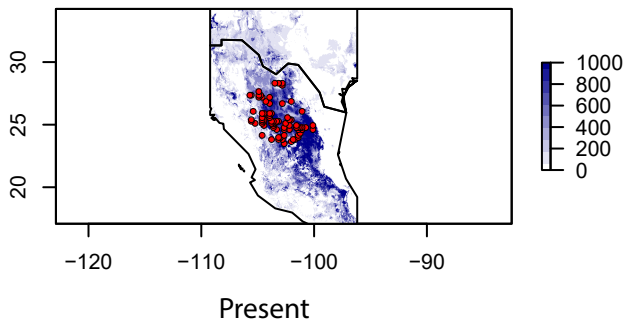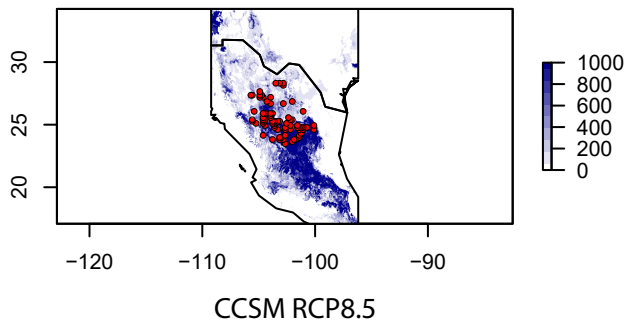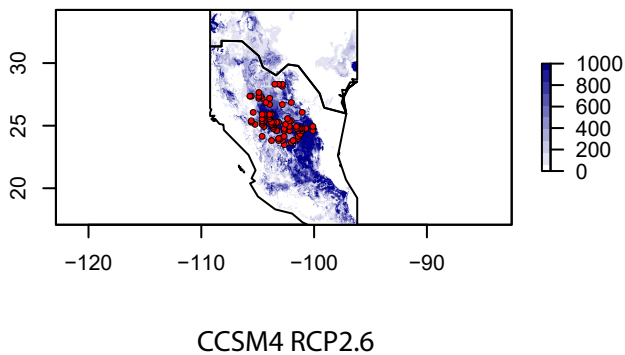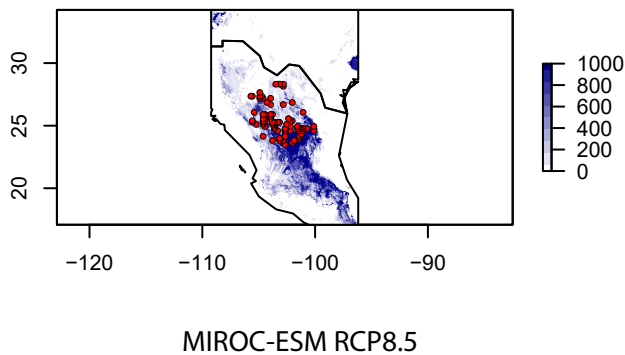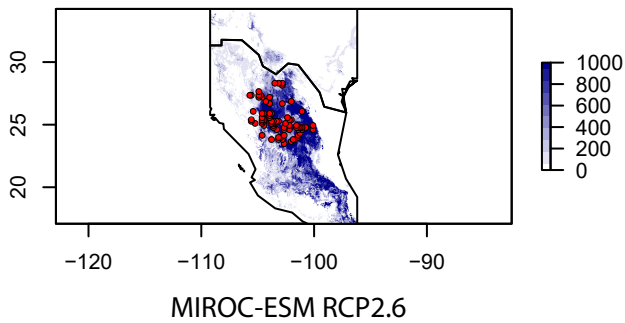

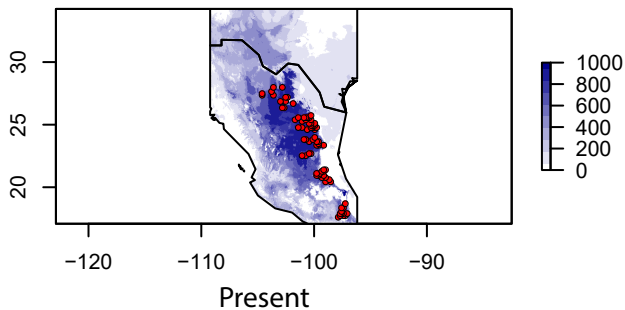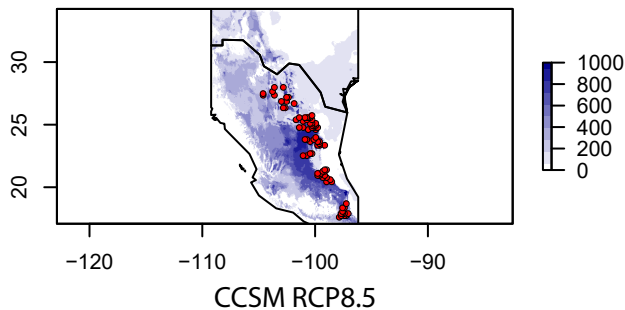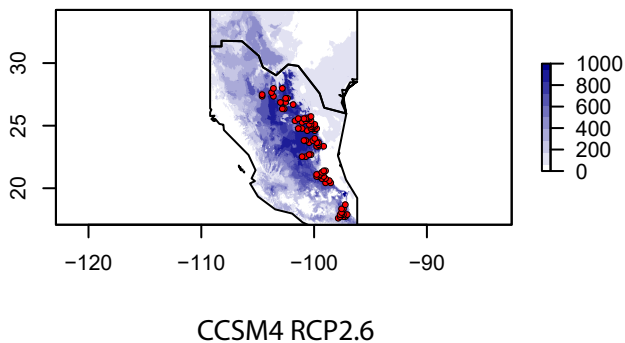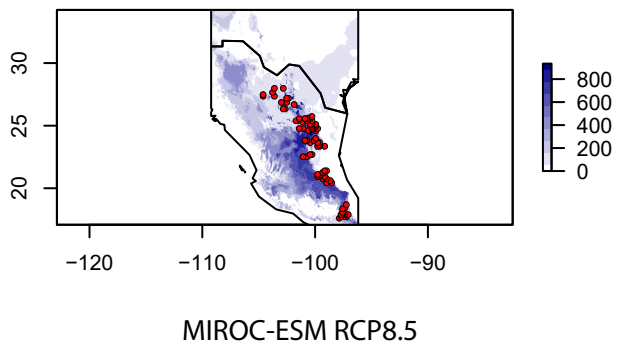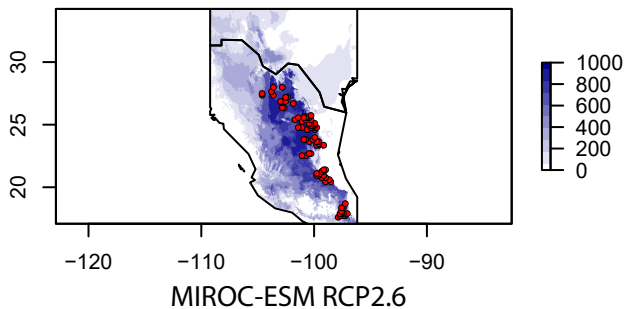

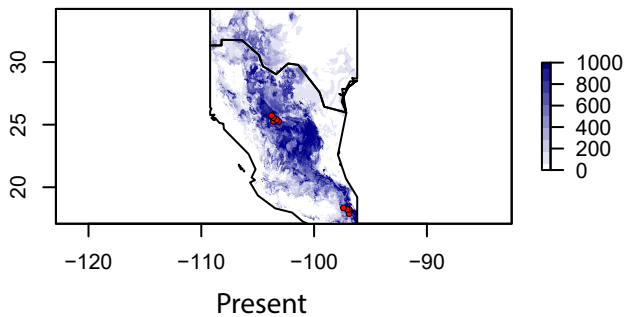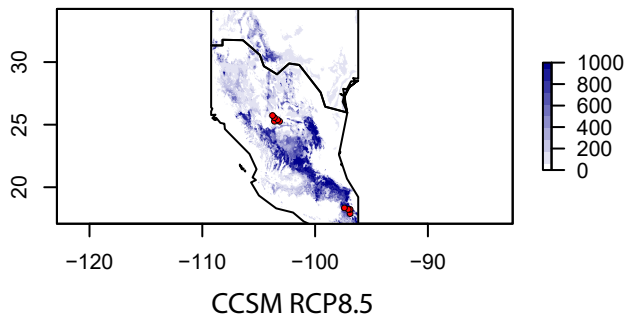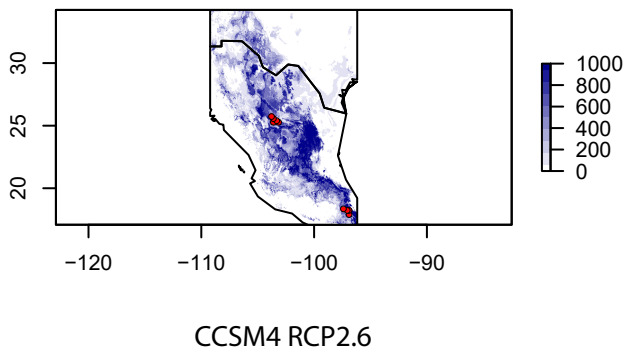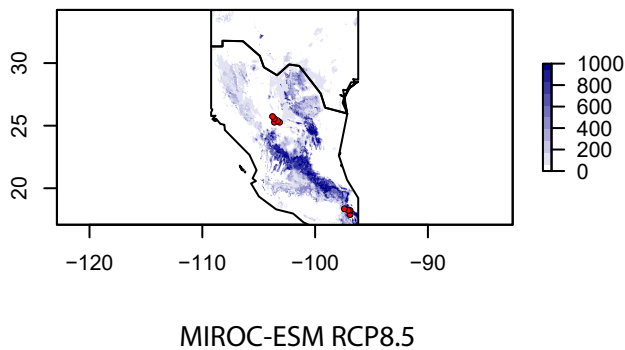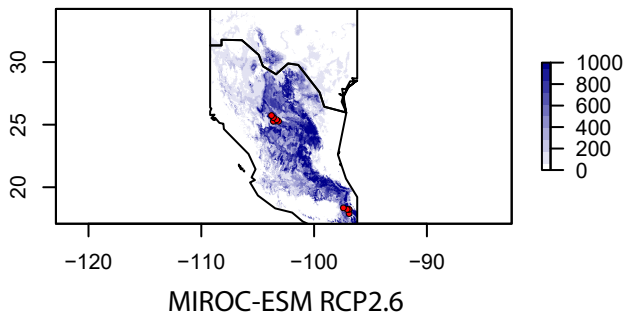

Supplement: Supplemental Information 6 — Geographic projections of inferred ecological niche models from the species studied for present and future GCMs climate scenarios CCSM4 and MIROC-ESM. [file peerj-07-6572-s006.pdf]
